# Reversal of the renal hyperglycemic memory in diabetic kidney disease by targeting sustained tubular p21 expression

**DOI:** 10.1038/s41467-022-32477-9

**Published:** 2022-08-27

**Authors:** Moh’d Mohanad Al-Dabet, Khurrum Shahzad, Ahmed Elwakiel, Alba Sulaj, Stefan Kopf, Fabian Bock, Ihsan Gadi, Silke Zimmermann, Rajiv Rana, Shruthi Krishnan, Dheerendra Gupta, Jayakumar Manoharan, Sameen Fatima, Sumra Nazir, Constantin Schwab, Ronny Baber, Markus Scholz, Robert Geffers, Peter Rene Mertens, Peter P. Nawroth, John H. Griffin, Maria Keller, Chris Dockendorff, Shrey Kohli, Berend Isermann

**Affiliations:** 1 Institute of Laboratory Medicine, Clinical Chemistry and Molecular Diagnostics, Universitätsklinikum Leipzig, Leipzig University, Leipzig, Germany; 2 Department of Medical Laboratories, Faculty of Health Sciences, American University of Madaba (AUM), Amman, Jordan; 3 Department of Biotechnology, University of Sargodha, Sargodha, Pakistan; 4 Internal Medicine I and Clinical Chemistry, German Diabetes Center (DZD), University of Heidelberg, Heidelberg, Germany; 5 Department of Medicine, Vanderbilt University Medical Center, Nashville, TN USA; 6 Institute of Pathology, University of Heidelberg, Heidelberg, Germany; 7 Leipzig Medical Biobank, Leipzig University, Leipzig, Germany; 8 Institute for Medical Informatics, Statistics and Epidemiology, Leipzig University, Leipzig, Germany; 9 Genome Analytics, Helmholtz Centre for Infection Research, Braunschweig, Germany; 10 Clinic of Nephrology and Hypertension, Diabetes and Endocrinology, Otto-von-Guericke University, Magdeburg, Germany; 11 Department of Molecular Medicine, The Scripps Research Institute, La Jolla, CA USA; 12 Helmholtz Institute for Metabolic, Obesity and Vascular Research (HI-MAG) of the Helmholtz Center Munich at the University of Leipzig and University Hospital Leipzig, Leipzig, Germany; 13 Medical Department III – Endocrinology, Nephrology, Rheumatology, University of Leipzig Medical Center, Leipzig, Germany; 14 Department of Chemistry, Marquette University, Milwaukee, WI USA

**Keywords:** Chronic kidney disease, DNA methylation, Senescence, Translational research, Diagnostic markers

## Abstract

A major obstacle in diabetes is the metabolic or hyperglycemic memory, which lacks specific therapies. Here we show that glucose-mediated changes in gene expression largely persist in diabetic kidney disease (DKD) despite reversing hyperglycemia. The senescence-associated cyclin-dependent kinase inhibitor p21 (*Cdkn1a*) was the top hit among genes persistently induced by hyperglycemia and was associated with induction of the p53-p21 pathway. Persistent p21 induction was confirmed in various animal models, human samples and in vitro models. Tubular and urinary p21-levels were associated with DKD severity and remained elevated despite improved blood glucose levels in humans. Mechanistically, sustained tubular p21 expression in DKD is linked to demethylation of its promoter and reduced DNMT1 expression. Two disease resolving agents, protease activated protein C (3K3A-aPC) and parmodulin-2, reversed sustained tubular p21 expression, tubular senescence, and DKD. Thus, p21-dependent tubular senescence is a pathway contributing to the hyperglycemic memory, which can be therapeutically targeted.

## Abstract (web summary)

Persistent diabetic complications despite controlled blood glucose levels, known as hyperglycemic memory, remain a poorly understood phenomenon in diabetic kidney disease. Here the authors identify senescence-associated gene p21 as a regulator of hyperglycemic memory, the suppression of which improves hyperglycemic memory and renal function.

## Introduction

Diabetic kidney disease (DKD) is now the leading cause of end-stage renal disease in industrialized countries, affecting approximately 40% of diabetic patients^1^. Current therapies, particularly blood sugar control and renin angiotensin aldosterone system (RAAS) inhibition, at best delay disease progression^2^. Sodium-glucose co-transporter-2 inhibitors (SGLT2i) are promising therapeutic adjuvants, but the long-term consequences, the associated risk profile and their ability to halt or even reverse disease progression remain largely unknown^3^. The persistence of diabetes-associated complications despite improved blood glucose control is known as metabolic or hyperglycemic memory^4^. Disease reversal, e.g., the improvement of already established DKD, remains an unmet medical need^2^. Accumulating evidence suggests that epigenetic mechanisms such as DNA methylation or posttranslational histone modifications are mechanistically linked to hyperglycemic memory^4^, but these insights have not yet led to new diagnostic or therapeutic approaches.

Several pathomechanisms have been linked to DKD^5^, yet their potential role in hyperglycemic memory is unknown. Senescence, reflecting permanent proliferative arrest, primarily affects renal tubular cells in chronic kidney disease, including DKD^6,7^. The severity of tubular senescence correlates with kidney injury, loss of renal function, and renal fibrosis^8,9^. Senescence is associated with inflammation and with mitochondrial reactive oxygen species (ROS), all hallmarks of DKD^8–10^.

The mitochondrial redox regulator p66^Shc^ is epigenetically induced in diabetic patients and mice, and p66^Shc^ deficiency protects against diabetic glomerulopathy in mice^11,12^. The cytoprotective and signaling-competent coagulation protease-activated protein C (aPC) reverses epigenetically induced p66^Shc^ expression in experimental diabetes^12,13^. These data illustrate that (i) epigenetic changes in diabetic vascular disease are in principle reversible and (ii) that the hyperglycemic memory is linked to endothelial dysfunction, as aPC’s plasma levels reflect endothelial function^14,15^.

Both hypomethylation and hypermethylation have been reported in association with diabetes and glomerular and tubulointerstitial damage in the context of DKD^4,16^. Tubulointerstitial damage in DKD is characterized by the accumulation of extracellular matrix (fibrosis), inflammation, and senescence of tubular cells^17^. The contribution of senescence to hyperglycemic memory in DKD remains unknown.

In the current study, we combined hypothesis-free approaches with analyses of human samples, genetic and interventional mouse models and in vitro studies to gain insights into mechanisms of hyperglycemic memory. We show that glucose-mediated changes in gene expression largely persist in DKD despite lowering glucose. The senescence associated gene *Cdkn1a* is a top regulator of hyperglycemic memory. Sustained *Cdkn1a* expression, depends on reduced DNMT1 mediated DNA methylation. Targeting sustained p21 expression by aPC or aPC-based therapies reverses persistent renal injury and improves renal function in DKD.

## Results

### Identification of genes and pathways associated with hyperglycemic memory

**Fig. 1:**
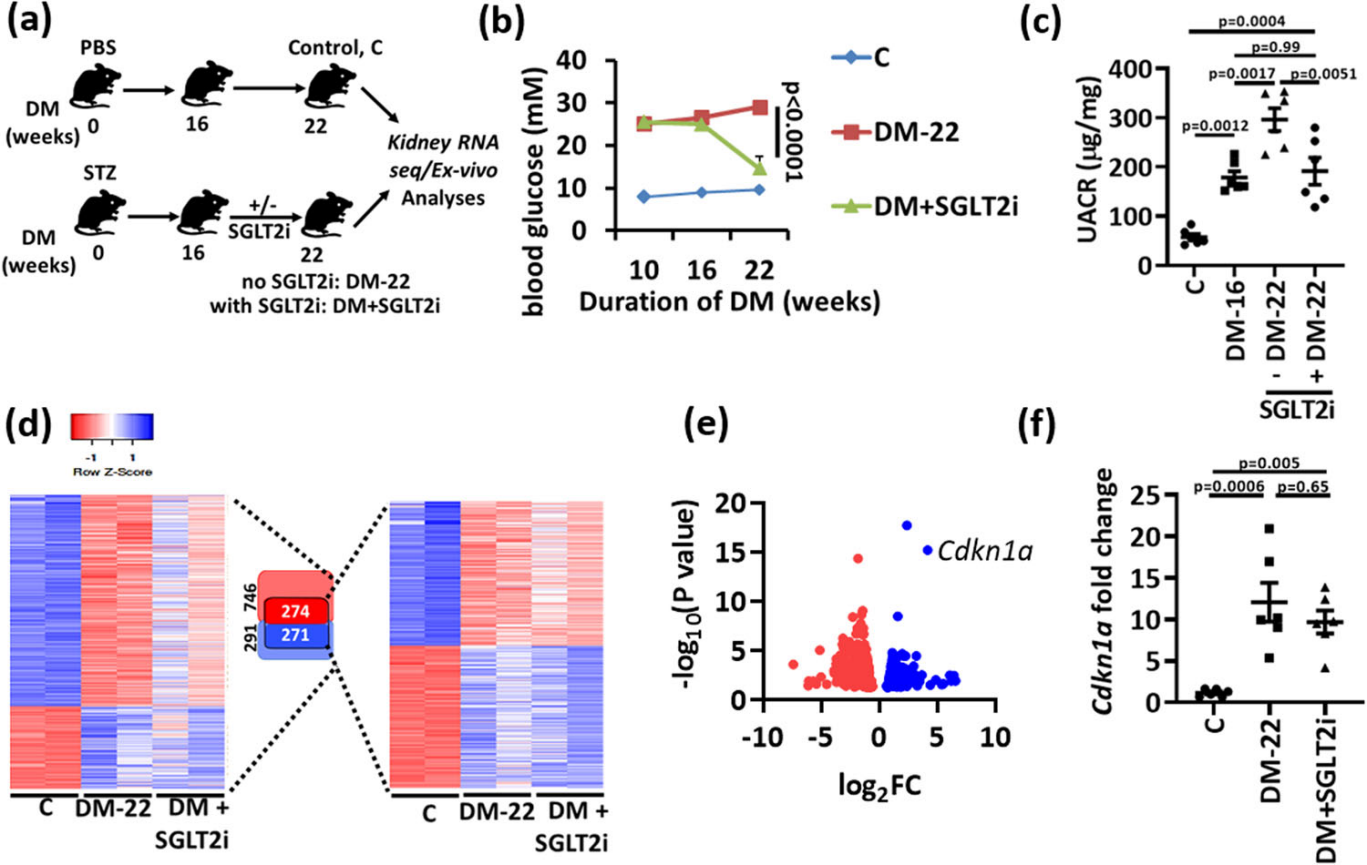
Identification of genes and pathways associated with hyperglycemic memory. **a** Experimental scheme showing non-diabetic control (C) or diabetic mice without (DM-22) or with intervention to reduce blood glucose levels by sodium/glucose cotransporter-2 inhibitor (Dapagliflozin®, DM + SGLT2i). Mice were age matched and SGLT2i treatment was started after 16 weeks of persistent hyperglycemia (streptozotocin; STZ-induced hyperglycemia). **b** Average blood glucose levels in the experimental groups after 10, 16 or 22 weeks. **c** Dot plot summarizing albuminuria (urinary albumin-creatinine ratio, µg albumin/mg creatinine; UACR) in non-diabetic (C) or diabetic mice without (DM-16 and DM-22) or with intervention to reduce blood glucose levels (DM + SGLT2i). **d** Heat map summarizing gene-expression in experimental groups (C, DM-22, DM + SGLT2i). All genes with significantly changed expression between control and diabetic mice are shown on the left side of the panel and are illustrated by lightly colored boxes in the middle (number of genes shown in black). The subset of genes with persistently changed expression despite reduced blood glucose levels are shown on the right side of the panel and illustrated by the darker colored smaller boxes in the middle (number of genes shown in white). **e** Volcano plot summarizing the persistently induced and repressed genes in diabetic mice after blood glucose reduction based on Log FC values and FDR. *Ckdn1a* (p21) belongs to the top persistently induced genes. **f** Sustained renal p21 mRNA expression (*Cdkn1a*, qRT-PCR) in vivo (DM-22; T1DM, STZ-model) despite reducing glucose levels (DM + SGLT2i; T1DM, STZ-model) as compared to mice with persistently elevated glucose levels (DM-22) or normoglycemic controls (C). Line graph and dot plots reflecting mean ± SEM of six mice per group; one-way ANOVA with Sidak’s multiple comparison test. Source data are provided as a Source Data file-Fig-1.

To identify regulators of renal hyperglycemic memory, we used a mouse model of hyperglycemia reversal and conducted hypothesis-free expression analyses (RNAseq). Persistent hyperglycemia was induced in mice for 16 weeks (using streptozotocin; see methods), at which stage mice had developed albuminuria (Fig. 1a–c). Subsequently, blood glucose levels were markedly reduced in a subgroup of mice for additional 6 weeks using an SGLT2 inhibitor (SGLT2i, Dapagliflozin®, DM + SGLT2i), while in another subgroup blood glucose levels remained elevated for the entire 22 weeks period (DM-22, Fig. 1a, b, Supplementary Fig. 1a). In age-matched non-diabetic control mice, SGLT2i had no impact on renal function or histology (Supplementary Fig. 1b–f). Reduction of blood glucose levels halted kidney function decline (DM-16 *versus* DM + SGLT2i), resulting in lower albuminuria compared to untreated diabetic mice (DM-22), but albuminuria remained increased compared to non-diabetic control mice (C, Fig. 1c). Thus, while lowering blood glucose levels halted disease progression, it failed to reverse already established albuminuria in mice, mimicking the hyperglycemic memory.

**Fig. 2:**
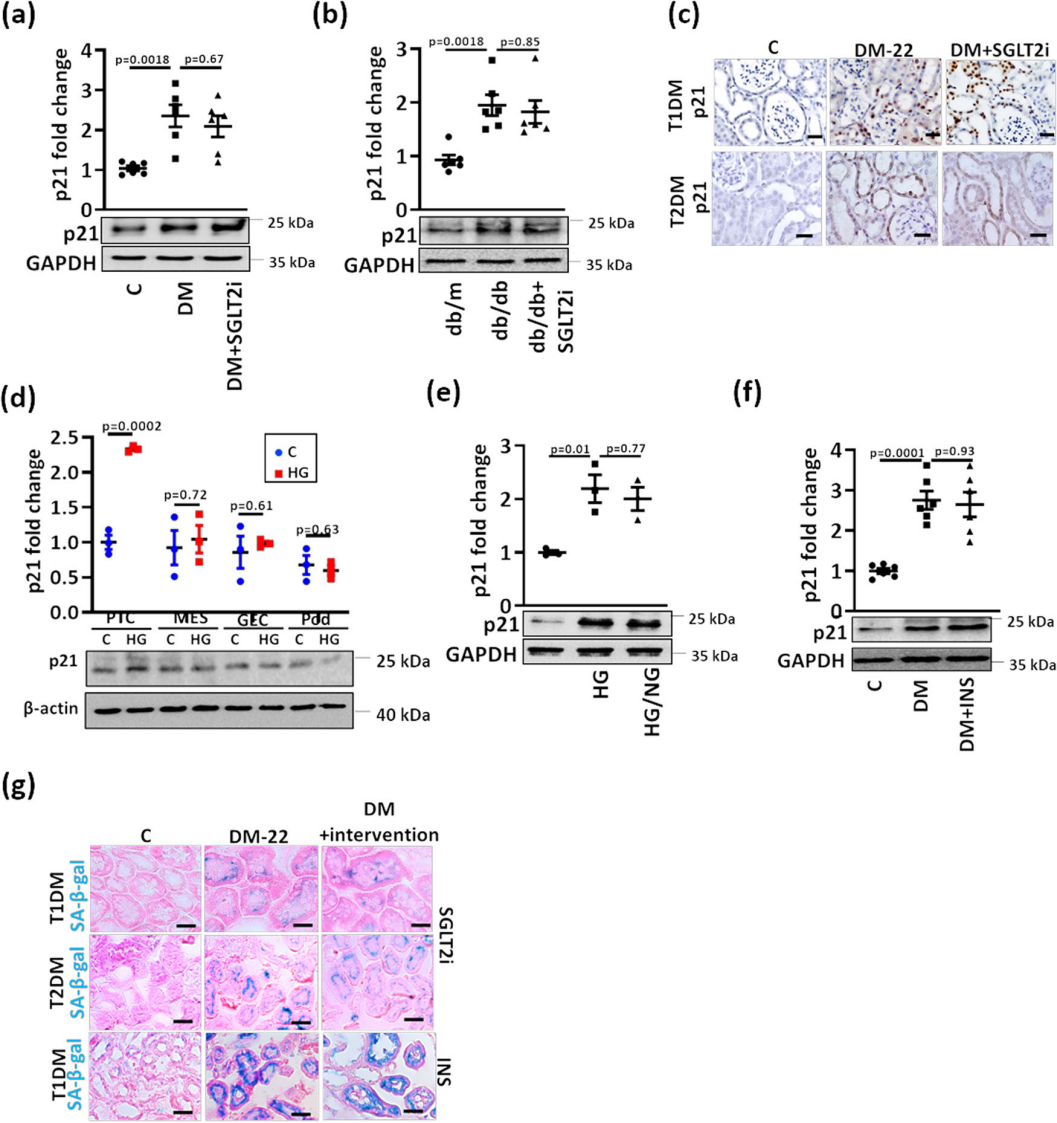
Sustained p21 expression despite glucose lowering in experimental DKD. **a**, **b** Sustained p21 expression in vivo (DM-22; T1DM, STZ-model, (**a**); T2DM, db/db-model, (**b**) despite reducing glucose levels (DM + SGLT2i, (**a**); db/db+SGLT2i, (**b**) as compared to mice with persistently elevated glucose levels (DM-22, (**a**); db/db, (**b**) or normoglycemic controls (C, **a**; db/m, **b**). Exemplary immunoblots (**a**, **b**). GAPDH: loading control (bottom, **a**, **b**) and dot plots summarizing results (top, **a**, **b**). **c** Immunohistochemical images of sustained p21 expression in kidneys of T1DM (STZ-model, top) and T2DM (db/db model, bottom) diabetic mice despite reducing glucose levels (DM + SGLT2i) as compared to mice with persistenly elevated blood glucose levels (DM-22); C: normoglycemic controls. p21 is detected by HRP-DAB reaction, brown; hematoxylin nuclear counter stain, blue. Scale bars represent 20 µm. **d** Exemplary p21immunoblot (bottom; loading control: β-actin) and dot plot summarizing results (top) in mouse primary tubular cells (PTC), mouse mesangial cells (MES), mouse glomerular endothelial cells (GEC), or mouse podocytes (Pod) maintained under normo- (5 mM; C) or high glucose (25 mM; HG) conditions. **e** Sustained p21 expression in vitro (Human kidney cells, HEK293) despite reducing glucose levels. HEK-293 cells were maintained under normal glucose (5 mM, C), high glucose (48 h of 25 mM, HG), or high glucose followed by normal glucose (25 and 5 mM, each for 24 h, HG/NG). Exemplary immunoblots (bottom, GAPDH: loading control) and dot plots summarizing results (top). **f** Sustained p21 expression in vivo (STZ-model) despite reducing glucose levels using insulin (DM + INS) as compared to mice with persistently elevated glucose levels (DM-22) and normoglycemic controls (C). Exemplary immunoblots (bottom, GAPDH: loading control) and dot plots summarizing results (top). **g** Exemplary histological images of SA-β-gal stain (senescence associated β-galactosidase, blue; eosin counterstain) in T1DM (STZ-model, top and bottom) and T2DM (db/db model, middle) despite reducing glucose levels (DM + intervention; SGLT2i, top and middle or insulin, INS, bottom) as compared to mice with persistently elevated glucose levels (DM-22) or normoglycemic controls (C). Scale bars represent 20 µm. Dot plots reflecting mean ± SEM of 6 mice per group or 3 independent experiments; one-way ANOVA with Sidak’s multiple comparison test (a,b,e,f); t-test comparing C versus HG for each cell line (d) Gels/blots were processed in parallel; source data are provided as a Source Data file-Fig-2.

To identify persistently altered gene expression in association with sustained albuminuria, RNAseq data were compared among nondiabetic control (C), untreated (DM-22) and SGLT2i-treated (DM + SGLT2i) diabetic mice. Compared to controls (C), 291 genes were induced, and 746 genes were suppressed in diabetic mice (DM-22, Fig. 1d). Despite reducing blood glucose levels in the SGLT2i-treated mice (DM + SGLT2i), the expression of 271 (out of 291) induced and 274 (out of 746) suppressed genes remained increased or suppressed, respectively, reflecting persistent gene expression possibly contributing to hyperglycemic memory (Fig. 1d). The cell-cycle and senescence associated gene *Cdkn1a* (p21) was among the top persistently induced genes (Fig. 1e). Functional annotation of these memorized genes revealed pathways related to metabolism (metabolic and endocrine pathways), growth factor signaling, chemokine and cytokine-mediated inflammation, and p53-p21 pathway and feedback loops as the most prevalent changes (Supplementary Fig. 1g). Persistent p21 induction was confirmed at mRNA (Fig. 1f) and protein level (Fig. 2a).

### Persistent induction of tubular p21 expression and senescence in experimental DKD

Induction of p21 in DKD has been reported, but its contribution to hyperglycemic memory has not been shown hitherto^18^. Using a model of type 2 diabetes mellitus (T2DM; db/db mice) and a similar experimental set up (persistent hyperglycemia until age 16 weeks followed by reduction of blood glucose levels using SGLT2i; Supplementary Fig. 2a–c), we likewise observed sustained albuminuria (Supplementary Fig. 2d) in association with persistent p21 induction in renal cortex extracts (Fig. 2b, Supplementary Fig. 2e).

Immunohistological analyses revealed persistently increased p21 expression despite lowering blood glucose levels in tubular cells in the T1DM and the T2DM models (Fig. 2c, Supplementary Fig. 3a, b). Predominant p21 induction in tubular cells by high glucose (HG) was confirmed in vitro comparing mouse primary tubular cells (PTCs), mesangial cells (MESs), glomerular endothelial cells (GECs), and podocytes (Pod, Fig. 2d) and in diabetic eNOS^−/−^ mice, an established murine model with severe DKD (Supplementary Fig. 3c, d). Induction of p21 expression in the tubular compartment preceded structural glomerular changes in diabetes models (STZ, db/db, Supplementary Fig. 3e–j), indicating that p21-induction in tubular cells is not a consequence of glomerular injury.

In line with the in vivo observations, high glucose concentrations (25 mM, HG) cell-autonomously induced p21 expression within 24 hours in human kidney (HEK-293, Fig. 2e) and mouse tubular (BUMPT, Supplementary Fig. 4a, b) cells, and p21 expression remained high despite lowering the glucose concentration to 5 mM for an additional 24 hours in both cell lines. Concomitant exposure of tubular cells (BUMPT) to SGLT2i, both in the presence of normal (5 mM) or high (25 mM) glucose concentration, prevented glucose uptake by tubular cells (Supplementary Fig. 4c) and had no impact on p21 expression (Supplementary Fig. 4a, b), suggesting that SGLT2i itself does not modulate p21.

To ascertain that persistent p21-expression is not a consequence of SGLT2i treatment, we lowered blood glucose levels in the T1DM model using insulin (Supplementary Fig. 5a). Blood glucose levels were efficiently reduced by both interventions, with insulin being slightly more efficient (Supplementary Fig. 5b, c). Despite blood glucose lowering by insulin, p21 expression in renal cortex extracts remained high (Fig. 2f), which was associated with persistent albuminuria and histological damage in tubular cells (Supplementary Fig. 5d–h).

As p21 is closely associated with senescence^19^, we performed senescence-associated β-galactosidase (SA-β-gal) staining. SA-β-gal staining was increased in tubular cells of diabetic mice (T1DM and T2DM models) and remained high despite reducing blood glucose levels using SGLT2i or insulin (Fig. 2g, Supplementary Fig. 6). Sustained p21 expression and SA-β-gal staining despite glucose lowering interventions were associated with other markers of senescence, such as induction of senescence associated secretors phenotype (SASP) genes (e.g. IL-1b, IL-6) and of cell-cycle kinase inhibitors (p15, p16, p19), reduced proliferation (Ki-67) and Lamin B1, and increased γ-H2A.X (Supplementary Fig. 7). Likewise, in HEK-293 cells and mouse PTC, the maintenance of 25 mM glucose for 24 h induced β-galactosidase staining, which did not normalize despite returning cells to normal glucose concentrations (5 mM) (Supplementary Fig. 8). Sustained β-galactosidase staining was associated with other markers of cellular senescence (induction of γ-H2A.X and SASP-factors, reduction of Ki-67 or Lamin B1) (Supplementary Fig. 8). Thus, p21 expression remains elevated despite the normalization of glucose levels and is associated with senescence markers in renal tubular cells in various models of DKD.

### Sustained tubular p21 expression and senescence despite glucose normalization in humans

**Fig. 3:**
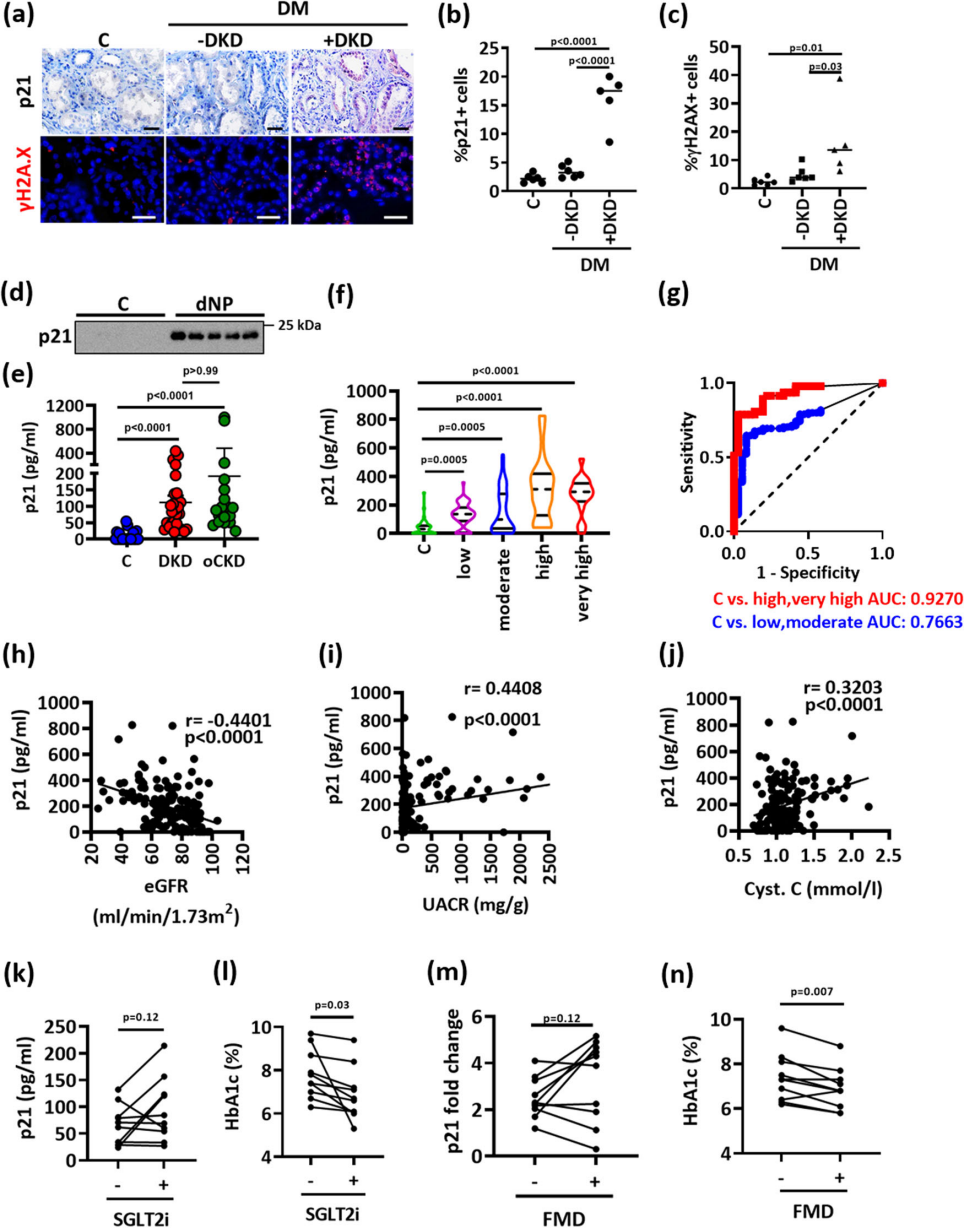
Sustained tubular p21 expression in human DKD. **a**–**c** Exemplary histological images of human kidney sections stained for p21 (top) or γ-H2A.X (histone H2A family X, bottom) obtained from non-diabetic controls (C; *n* = 6) or diabetic patients without (DM-DKD; *n* = 6) or with (DM + DKD; *n* = 5) DKD; p21 is detected by HRP-DAB reaction, brown; hematoxylin nuclear counter stain, blue; γ-H2A.X is immunofluorescently detected, red; DAPI nuclear counterstain, blue. Dot plots summarizing results for p21 (**b**) and γ-H2A.X (**c**); Scale bars represent 20 μm. one-way ANOVA with Sidak’s multiple comparison test. **d**, **e** Urinary p21, detected by immunoblotting (**d**) or ELISA (**e**; pg/ml) is markedly increased in patients with DKD (*n* = 26) compared to non-diabetic controls with normal kidney function (C; *n* = 22). Urinary p21 levels in patients with other causes of chronic kidney disease (oCKD; *n* = 18) are comparable to those in patients with DKD. Exemplary immunoblot (d) and dot plots reflecting mean ± SD showing the distribution of the urinary levels of p21 (**e**, Kruskal–Wallis test with Dunn’s multiple comparison test. **f** Violin plots showing the distribution of the urinary levels of p21 (pg/ml; ELISA) in normoglycemic controls (C; *n* = 36) and diabetic individuals which are classified according to KDIGO criteria from the LIFE-adult cohort (low risk, n = 52; moderate risk, *n* = 53; high risk, *n* = 29 and very high risk, n = 18). Kruskal-Wallis test with Dunn’s multiple comparison test. **g** Receiver operating characteristic (ROC) analyses of urinary p21 (pg/ml; ELISA) in diabetic individuals with low or moderate risk of CKD (blue) and in diabetic patients with high or very high risk of CKD (red) compared to non-diabetic controls (C). AUC: area under the curve. **h**–**j** Negative correlation of urinary p21 (pg/ml, ELISA) with estimated glomerular filtration rate (eGFR, ml/min/1.73 m², (**h**) and positive correlation with urinary albumin creatinine ration (UACR, mg albumin /g creatinine, (**i**) or with cystatin C serum levels (Cyt. C, mmol/l, **j**) in diabetic individuals from the LIFE-adult cohort. Pearson’s correlation. **k**–**n** line-graphs illustrating individual changes of urinary p21 (**k**; ELISA, pg/ml, **m**; immunoblot) or HbA1c (**l**, **n**) in diabetic patients with known DKD (*n* = 10) before (−) and after (+) SGLT2i treatment (**k**, **l**) or before (−) and after (+) fasting mimicking diet (FMD, **m**, **n**). Paired t-test. Gels/blots were processed in parallel; source data are provided as a Source Data file-Fig-3.

To scrutinize the relevance of these findings in the context of human DKD, we first analyzed p21 expression by immunohistochemistry. Tubular p21 expression was increased in renal biopsies obtained from diabetic patients with DKD (DM + DKD) but not in diabetic patients without DKD (DM-DKD) or non-diabetic controls (C, Supplementary Table 1, Fig. 3a, b). Tubular p21 expression was associated with increased γ-H2A.X staining (phosphorylated H2A histone family member X, a marker of DNA damage and senescence^20^) in patients with DKD (Fig. 3a, c).

Biopsies from diabetic patients are not routinely obtained, precluding monitoring of tubular p21 expression. Hence, we determined whether p21 can be detected in urine samples. p21 was readily detectable by immunoblotting in randomly chosen urinary samples from diabetic patients with known DKD (*N* = 5), but not in healthy controls (*N* = 6, Fig. 3d). We next determined urinary p21 in a group of patients with known DKD (*N* = 26) and compared these to patients with normal renal function (*N* = 22, Supplementary Table 2), both recruited from the local outpatient clinic. Urinary p21 was readily detectable by ELISA and was on average much higher in patients with DKD (26 out of 26 positive) in comparison to outpatients with other diseases, but normal renal function (6 out of 22 positive, Fig. 3e).

To determine whether tubular p21 expression is specific for DKD or may be observed in other forms of chronic kidney disease (CKD), we obtained renal biopsies from patients with CKD unrelated to diabetes mellitus. Again, we observed p21 expression predominately in tubular cells (Supplementary Fig. 9a). Likewise, in a different group of patients with CKD unrelated to diabetes mellitus, again recruited from the local outpatient clinic, we observed increased urinary p21 levels (Fig. 3e, Supplementary Table 2). These results suggest that tubular p21 expression is not specific for DKD but may be a hallmark of non-reversible CKD (“nrCKD”).

We next analyzed urinary p21 in DKD in a larger cohort. We identified non-diabetic and diabetic individuals in a large cross-sectional cohort (LIFE-Adult, Supplementary Table 3)^21^. The severity of DKD was classified according to the KDIGO criteria^22^. Urinary p21 was again significantly increased in diabetic participants compared to non-diabetic individuals (Fig. 3f). Urinary p21 levels were already increased in diabetic patients with a low or moderate risk of CKD, and further increased in patients with high or very high risk of CKD (Fig. 3f). ROC-analyses of urinary p21 revealed an AUC of 0.7663 for the detection of patients at a low or moderate CKD risk, while the AUC was 0.9270 for the detection of patients at a high or very high risk of CKD (Fig. 3g), corroborating that urinary p21 increases with the severity of kidney disease. Accordingly, urinary p21 was inversely correlated with the glomerular filtration rate (GFR) and directly correlated with albuminuria and cystatin C serum levels in diabetic patients (Fig. 3h–j).

To analyze whether p21 levels remain elevated in patients with DKD despite blood glucose reduction, we assessed urinary p21 in diabetic patients before and after blood glucose lowering resulting from SGLT2i treatment (Supplementary Table 4) or from a fasting-mimicking diet^23^ (Supplementary Table 5). Urinary p21 was increased in patients with DKD and remained high despite blood glucose reduction in most of these patients (Fig. 3k–n, Supplementary Fig. 9b–d).

Taken together, (i) the increase of tubular p21 expression in diabetic patients with DKD, but not in diabetic patients without DKD, (ii) the increase of urinary p21 in diabetic patients in association with an increasing KDIGO grade of CKD, and (iii) the persistent increase of urinary p21 despite improved blood glucose levels in diabetic patients support a model in which renal tubular p21 expression reflects persistent tubular damage in DKD, contributing to the hyperglycemic memory. These data raise the question of whether reversing p21 induction may “erase” the hyperglycemic memory.

### aPC reverses glucose induced p21 promoter methylation and sustained p21 expression

**Fig. 4:**
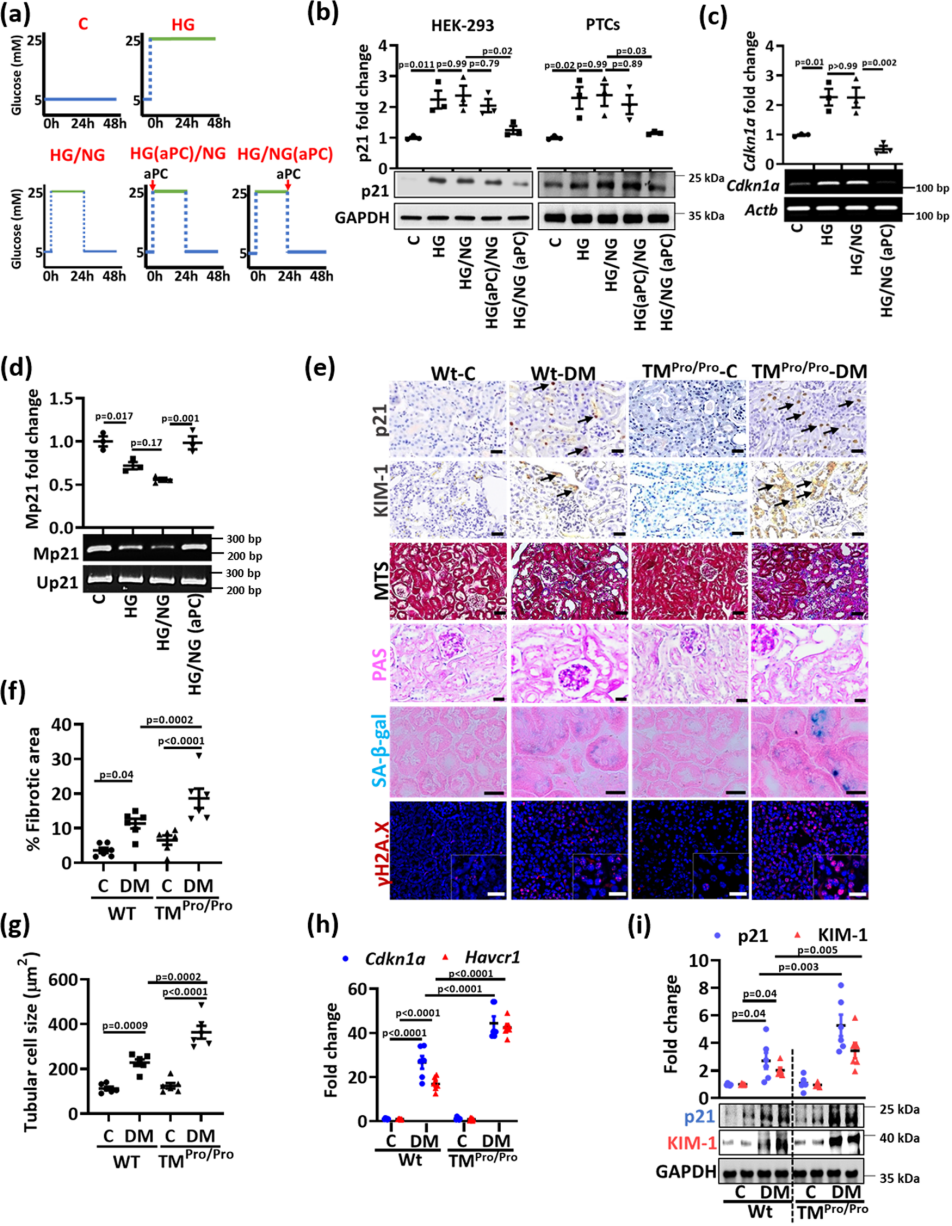
Impaired protein C activation increases tubular p21 expression and senescence in vivo. **a** Experimental scheme of in vitro experiments with human kidney cells (HEK-293) or mouse primary tubular cells (PTCs). Experimental conditions: control with continuously normal glucose (C, 5 mM glucose), continuously high glucose (HG, 25 mM, 48 h), high glucose for 24 h followed by normal glucose (NG, 5 mM glucose) for 24 h without aPC-exposure (HG/NG), with aPC (20 nM) during the high glucose exposure (HG(aPC)/NG), or with aPC (20 nM) upon returning cells to normal glucose (HG/NG(aPC)). **b** Exemplary immunoblots of p21 (bottom; loading control: GAPDH) in HEK-293 cells and mouse PTCs and dot plots summarizing results (top). Experimental conditions as described in (**a**). **c**, **d** Exemplary images (bottom) and dot plots summarizing results (top) of p21 (*Cdkn1a*) mRNA-levels determined by semiquantitative RT-PCR (control: β-actin, *Actb*; c) and methylation-specific PCR (d) of the p21-promoter (Mp21; control: unmethylated p21, Up21) in HEK-293 cells (experimental conditions as described in **a**). **e** Exemplary histological images from non-diabetic (C) or diabetic (DM) wild-type (Wt) and TM^Pro/Pro^ mice for p21 (immunohistochemically detected by HRP-DAB-reaction, brown, examples illustrated by arrows; hematoxylin counterstain), interstitial fibrosis (Masson’s trichrome stain, MTS), periodic acid Schiff stain (PAS), SA-β-gal. stain (senescence associated β-galactosidase, blue; eosin counterstain), and γ-H2A.X immunohistochemistry (histone H2A family X, red; DAPI nuclear counterstain, insets: larger magnification); all scale bars represent 20 μm. **f**, **g** Dot plots summarizing tubular fibrotic area (**f**) and tubular cell size (**g**) in experimental groups (as described in (**e**). **h** Dot plot summarizing renal p21 (*Cdkn1a*) and KIM-1 (*Havcr1*) expression (mRNA, qRT-PCR) in experimental groups (as described in **e**). **i** Representative immunoblots (bottom; loading control: GAPDH) and dot plot summarizing results (top) for renal p21 and KIM-1 expression in experimental groups (as described in **e**). Dot plots reflecting mean ± SEM of 3 independent experiments (**b**–**d**) or 6 mice per group (**f**–**i**); one-way ANOVA with Sidak’s multiple comparison test (**b**–**d**) or two-way ANOVA with Sidak’s multiple comparison test (**f**–**i**). Gels/blots were processed in parallel; source data are provided as a Source Data file-Fig-4.

We have previously shown that the coagulation protease aPC reverses epigenetically induced p66^Shc^ expression in diabetic vascular diseases^12,13^. To determine whether aPC reverses glucose-induced and sustained p21 expression, we conducted in vitro experiments using HEK-293 and mouse PTCs. HG induced p21 expression in HEK-293 and PTCs, and the expression remained high despite normalization of glucose levels (HG/NG, Fig. 4a–c). Exposure of cells to aPC (20 nM) along with high glucose concentrations had no impact on p21 induction or sustained p21 expression during the subsequent 24 hours of low glucose concentrations (HG(aPC)/NG, Fig. 4a, b). In contrast, the addition of aPC at the time of glucose normalization reduced p21 expression (HG/NG(aPC), Fig. 4a–c).

In view of perpetuated p21 mRNA expression in tubular cells, we next analyzed p21 promoter methylation. HG-induced p21 mRNA expression was associated with reduced p21 promoter methylation (Fig. 4c, d). Restoring low glucose concentrations had no impact on p21 mRNA expression and p21 promoter hypomethylation (HG/NG, Fig. 4c, d), while the addition of aPC (20 nM) at the time glucose levels were reduced fully restored p21 promoter methylation and reduced p21 mRNA expression (HG/NG(aPC), Fig. 4c, d).

To determine whether the aPC-dependent p21 suppression in tubular cells reduces senescence, we analyzed PTCs. The sustained induction of senescence-associated markers (increased SASP-factors, γ-H2A.X, β-galactosidase staining and decreased Ki-67, Lamin B1) in PTCs upon exposures to 25 mM glucose for 24 h followed by 24 h of normal glucose levels (5 mM) was normalized upon addition of aPC (20 nM) at the time of returning cells to normal glucose concentrations (Supplementary Fig. 10). These results show that aPC reverses glucose-induced, sustained p21 expression, associated p21 promoter hypomethylation and markers of senescence in renal tubular cells.

### Impaired protein C activation increases tubular p21 expression and senescence in vivo

Hyperglycemia is associated with endothelial dysfunction and impaired thrombomodulin (TM)-dependent protein C activation^14,15^. To determine whether impaired TM-dependent protein C activation aggravates glucose-induced tubular p21 expression and senescence, we analyzed mice with low endogenous aPC generation secondary to a point mutation in the TM gene (*Thbd*, Glu404Pro, TM^Pro/Pro^ mice)^24^. This point mutation mimics impaired TM-dependent protein C activation due to oxidative damage^25^. If diabetic, TM^Pro/Pro^ mice display increased glomerular damage^15^, but the consequences for tubular damage and tubular senescence have not been investigated hitherto. In addition to albuminuria and normalized kidney weight, interstitial fibrosis and tubular cell size (reflecting cellular hypertrophy) were enhanced in diabetic TM^Pro/Pro^ mice after 22-weeks of persistent hyperglycemia as compared to diabetic wild-type littermates despite comparable blood glucose levels (Fig. 4e–g, Supplementary Fig. 11a–e). Concomitantly, p21 (*Cdkn1a*) and KIM-1 (kidney injury molecule-1, *Havrc1*) expression (mRNA and protein levels) were increased in diabetic TM^Pro/Pro^ mice as compared to diabetic wild-type mice (Fig. 4e, h, i, Supplementary Fig. 11f). The increased tubular cell hypertrophy and p21 expression indicate enhanced tubular cell senescence, which is supported by greater SA-β-gal and γ-H2A.X staining (Fig. 4e, Supplementary Fig. 11g, h) and induction of SASP genes in diabetic TM^Pro/Pro^ mice (Supplementary Fig. 12a). Changes of other markers reflecting senescence (p15, p16, p19, Ki-67 and Lamin B1) were comparable between diabetic wild type and diabetic TM^Pro/Pro^ mice (Supplementary Fig. 12b–e). These data show that impaired endogenous PC activation aggravates tubulointerstitial damage and tubular cell senescence in DKD, potentially in a p21-dependent fashion.

### p21 mediates enhanced tubular senescence in aPC-deficient mice

**Fig. 5:**
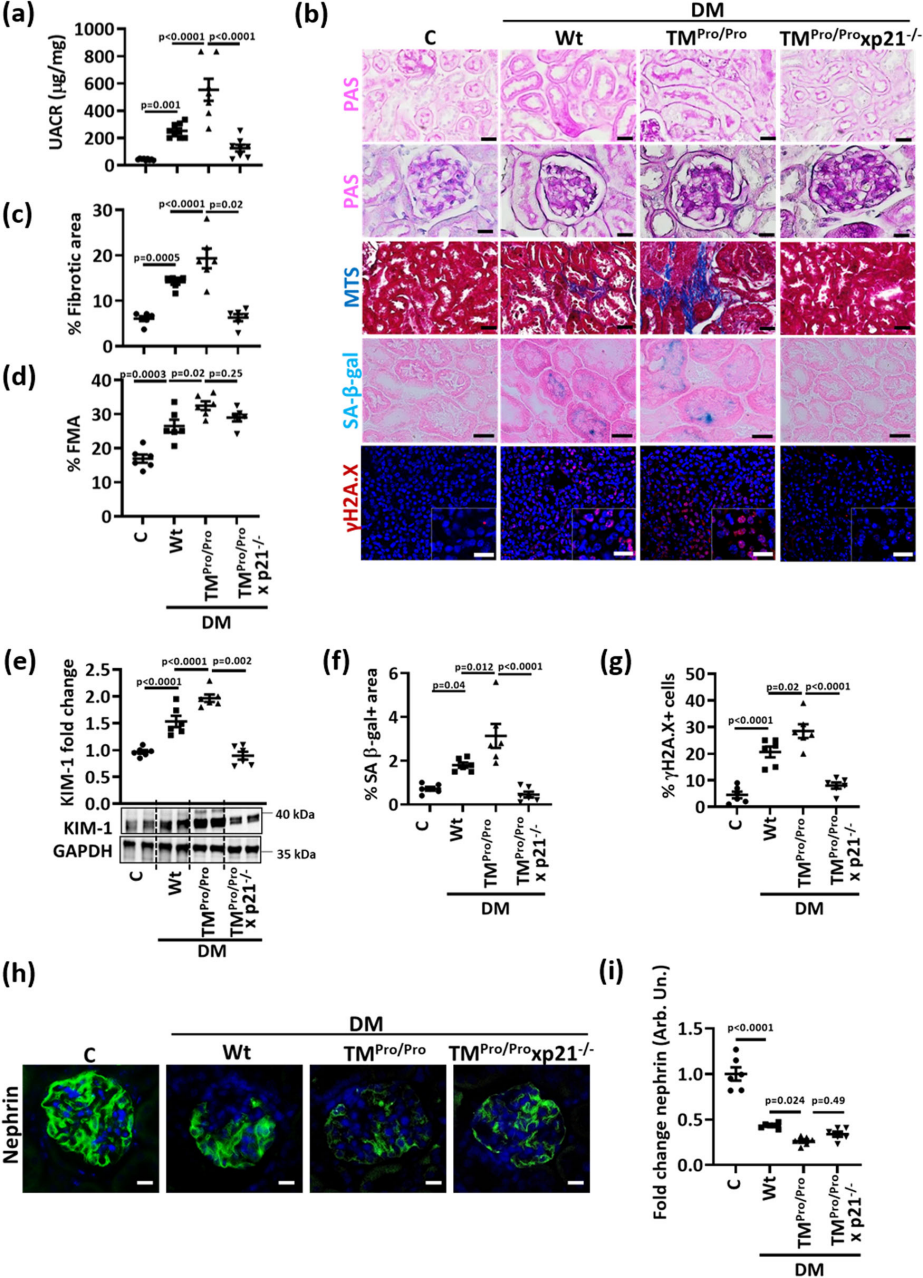
p21 mediates enhanced tubular senescence in aPC-deficient mice. **a** Dot plot summarizing albuminuria (urinary albumin-creatinine ratio, µg albumin/mg creatinine; UACR) in non-diabetic (C, *n* = 9) or diabetic (DM) wild-type (Wt, *n* = 8), TM^Pro/Pro^ (*n* = 7) and TM^Pro/Pro^ x p21^−/−^ (*n* = 8) mice. **b** Exemplary histological images of periodic acid Schiff stains (PAS) showing tubuli (top) or glomeruli (2^nd^ row), interstitial fibrosis (Masson’s trichrome stain, MTS), SA-β-gal. stain (senescence associated β-galactosidase, blue; eosin counterstain), and γ-H2A.X immunohistochemistry (histone H2A family X, red; DAPI nuclear counterstain, insets: larger magnification) in experimental groups (as described in **a**); all scale bars represent 20 μm. **c**, **d** Dot plots summarizing tubular fibrotic area (**c**) and fractional mesangial area (FMA, **d**), the latter reflecting glomerulosclerosis in experimental groups (as described in **a**). **e** Exemplary immunoblot (bottom; loading control: GAPDH) and dot plot summarizing results (top) of renal KIM-1 expression in experimental groups (as described in **a**). **f**, **g** Dot plots summarizing percentage of SA-β-gal. positive area (**f**) and percentage of γ-H2A.X positive cells (**g**) in experimental groups (as described in **a**). **h**, **i** Exemplary histological images of glomerular nephrin expression (green, DAPI nuclear counterstain, blue, **h**), and dot plot summarizing nephrin staining intensity fold change (**i**) in experimental groups (as described in **a**). Dot plots reflecting mean ± SEM of six mice per group; one-way ANOVA with Sidak’s multiple comparison test. Source data are provided as a Source Data file-Fig-5.

To analyze the relevance of p21 for increased tubulointerstitial damage and tubular senescence in diabetic aPC-deficient mice, we analyzed TM^Pro/Pro^ mice with genetically superimposed p21 deficiency (TM^Pro/Pro^ x p21^−/−^). Despite comparable blood glucose levels among diabetic mice (22 weeks of persistent hyperglycemia), genetically superimposed p21 deficiency markedly reduced albuminuria and normalized kidney weight in hyperglycemic TM^Pro/Pro^ mice (Fig. 5a, Supplementary Fig. 13a, b). In parallel, tubular and interstitial damage (fibrotic area, KIM-1 expression, Fig. 5b–e) as well as markers of tubular cell senescence (SA-β-gal, γ-H2A.X, proliferation (Ki-67) and Lamin B1 expression, Fig. 5b, f, g and Supplementary Fig. 13c–e) were improved in TM^Pro/Pro^ x p21^−/−^ mice. Remarkably, indices of glomerular injury, such as extracellular matrix accumulation (FMA, fractional mesangial area, Fig. 5b, d) or reduced podocyte marker expression (nephrin, synaptopodin, WT-1, Fig. 5h, i, Supplementary Fig. 14), were not improved in TM^Pro/Pro^ x p21^−/−^ mice, indicating that p21 induction causes primarily tubular damage in mice with low aPC levels. Taken together, the increased tubulointerstitial damage and senescence in diabetic TM^Pro/Pro^ mice depends on p21 expression, supporting a model in which thrombomodulin-dependent aPC-generation regulates tubular p21 expression and senescence in DKD.

### High glucose and aPC differentially regulate renal DNMT1 expression

**Fig. 6:**
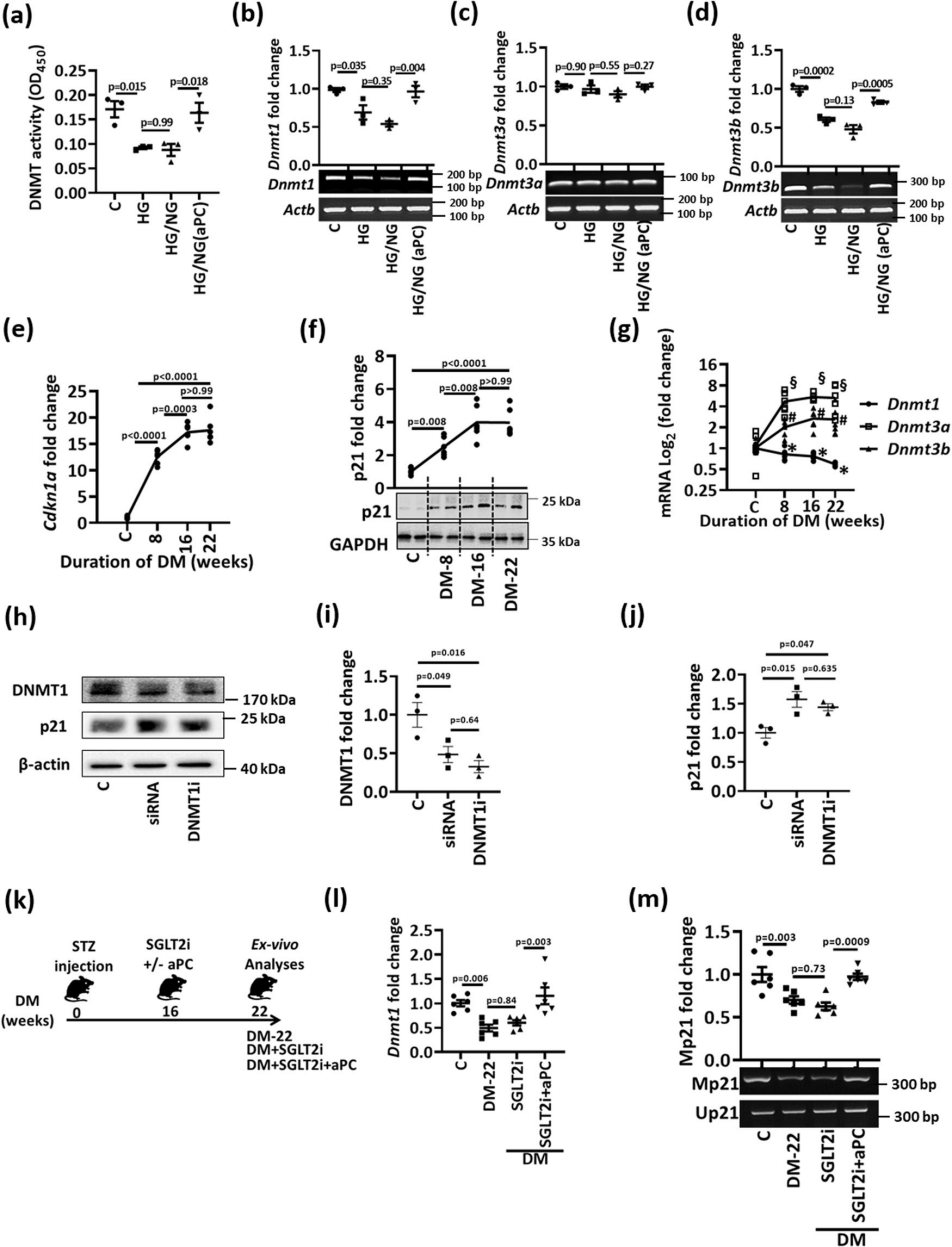
aPC regulates p21 promoter methylation and DNMT1 in tubular cells. **a** Dot-plot summarizing total DNMT-activity in HEK-293 cells. Experimental conditions: control with continuously normal glucose (C, 5 mM glucose), continuously high glucose (HG, 25 mM, 48 h), high glucose for 24 h followed by normal glucose (NG, 5 mM glucose) for 24 h without aPC-exposure (HG/NG), or with aPC (20 nM) upon returning cells to normal glucose (HG/NG(aPC)). **b**–**d** Exemplary gel-images of *Dnmt1* (**b**), *Dnmt3a* (**c**), and *Dnmt3b* (**d**); (bottom; semi-quantitative RT-PCR, loading control: β-actin, *Actb*) expression and dot-plots summarizing results (top) in experimental conditions (as described in **a**). **e**–**g** Kinetics of renal p21 (*Cdkn1a*) expression (**e**, mRNA, qRT-PCR; **f**, protein expression). Exemplary immunoblot (f, bottom; GAPDH: loading control) and line-graph summarizing results (top). Line-graph summarizing *Dnmt1, Dnmt3a* and *Dnmt3b* expression in vivo (**g**, mRNA, qRT-PCR). Diabetic wild-type mice (STZ model, age 8, 16, or 22 weeks) were compared to non-diabetic mice (C, age 30 weeks). *P = 0.022 (C vs 8), *P = 0.002 (C vs 16), *P < 0.0001 (C vs 22). #*P* = 0.001 (C vs 8), #*P* = 0.0002 (C vs 16), #*P* = 0.0003 (C vs 22). §*P* = 0.15 (C vs 8), §*P* = 0.007 (C vs 16), §*P* = 0.011 (C vs 22). **h**–**j** Exemplary immunoblots of DNMT1 and p21 (β-actin: loading control) in murine primary tubular cells (**h**) and dot-plots summarizing results of DNMT1 (**i**) and p21 (**j**). Experimental conditions: control (C), DNMT1 siRNA (24 h, siRNA), and DNMT1 vivo morpholino (10 µM, 24 h, DNMT1(**i**). **k** Experimental scheme: 16 weeks after induction of persistent hyperglycemia with STZ, diabetic mice were treated with PBS (DM-22), sodium/glucose cotransporter 2-inhibitor (Dapagliflozin®, DM + SGLT2i) or a combination of SGLT2i and aPC (DM + SGLT2i + aPC) for further 6 weeks. **l**, **m** Dot-plots summarizing renal *Dnmt1* expression (i, mRNA, qRT-PCR), and exemplary images of methylation-specific PCR (**j**, bottom) and dot-plot summarizing results (**j**, top) of p21-promoter (Mp21; control: unmethylated p21, Up21) in experimental groups (as described in **h**). Dot-plots or line-graphs reflecting mean ± SEM of three independent experiments (**a**–**d**, **i**, **j**), 4 mice per group (**e**–**g**), or 6 mice per group (**i**, **m**); one-way ANOVA with Sidak’s multiple comparison test. Gels/blots were processed in parallel; source data are provided as a Source Data file-Fig-6.

We next investigated the mechanism regulating persistent tubular p21 expression and p21-promoter demethylation in hyperglycemic conditions. As DNA methylation depends on the activity of DNA methyltransferases (DNMTs), we analyzed total DNMT activity. HG reduced DNMT activity in HEK-293 cells in vitro, which remained low despite normalization of glucose levels (HG/NG), but was restored upon concomitant exposure to aPC (20 nM, HG/NG(aPC), Fig. 6a). Expression analyses of individual DNMTs revealed that HG induced sustained suppression of DNMT1 and DNMT3b but had no impact on DNMT3a (Fig. 6b–d). Addition of aPC at the time of returning cells to normal glucose concentrations restored DNMT1 and DNMT3b expression in vitro (Fig. 6b, d).

To scrutinize the relevance of p21 and DNMTs in vivo, we first determined the expression of p21 and DNMTs at different time points after the induction of persistent hyperglycemia. An increase of p21 mRNA and protein expression was already detectable 8 weeks after induction of hyperglycemia and increased further until week 16 (Fig. 6e, f). In parallel, we observed a reduction in DNMT1 in vivo, whereas DNMT3a and DNTM3b were induced (Fig. 6g). As epigenetic control is finely tuned by methylating and demethylating enzymes, we screened additional epigenetic regulators previously linked with DKD^4,26^. Among these, expression of Tet2 and Kdm6a were persistently induced despite glucose lowering by SGLT2i or insulin (Supplementary Fig. 15). As suppression of DNMT1 in BUMPT cells and primary murine tubular cells was sufficient to induce p21 expression (Fig. 6h–j, Supplementary Fig. 16), we focused on the role of DNMT1 in regulating p21 expression in DKD.

To determine whether DNMT1 suppression is memorized in diabetic mice and whether aPC can reverse memorized DNMT1 suppression in vivo, we again reversed hyperglycemia in mice after 16 weeks for an additional 6 weeks (DM + SGLT2i, Fig. 6k). A subgroup of mice received aPC in addition to the SGLT2i following an established protocol (DM + SGLT2i + aPC, Fig. 6k)^12^. In mice with persistent hyperglycemia for 22 weeks (DM-22), DNMT1 expression was reduced (Fig. 6l). A reduction of blood glucose levels by SGLT2i from weeks 16 to 22 did not restore DNMT1 expression (DM + SGLT2i, Fig. 6l), while addition of aPC restored DNMT1 expression (DM + SGLT2i + aPC, Fig. 6l). Parallel to restoring DNMT1 expression, aPC reversed hyperglycemia-induced persistent hypomethylation of the p21 promoter (Fig. 6m). These results support a model in which reduced DNMT1 expression is linked with p21 promoter hypomethylation and increased p21 expression in DKD, which can be reversed by aPC.

### aPC reverses glucose-induced and sustained renal p21 expression and tubular injury via DNMT1

**Fig. 7:**
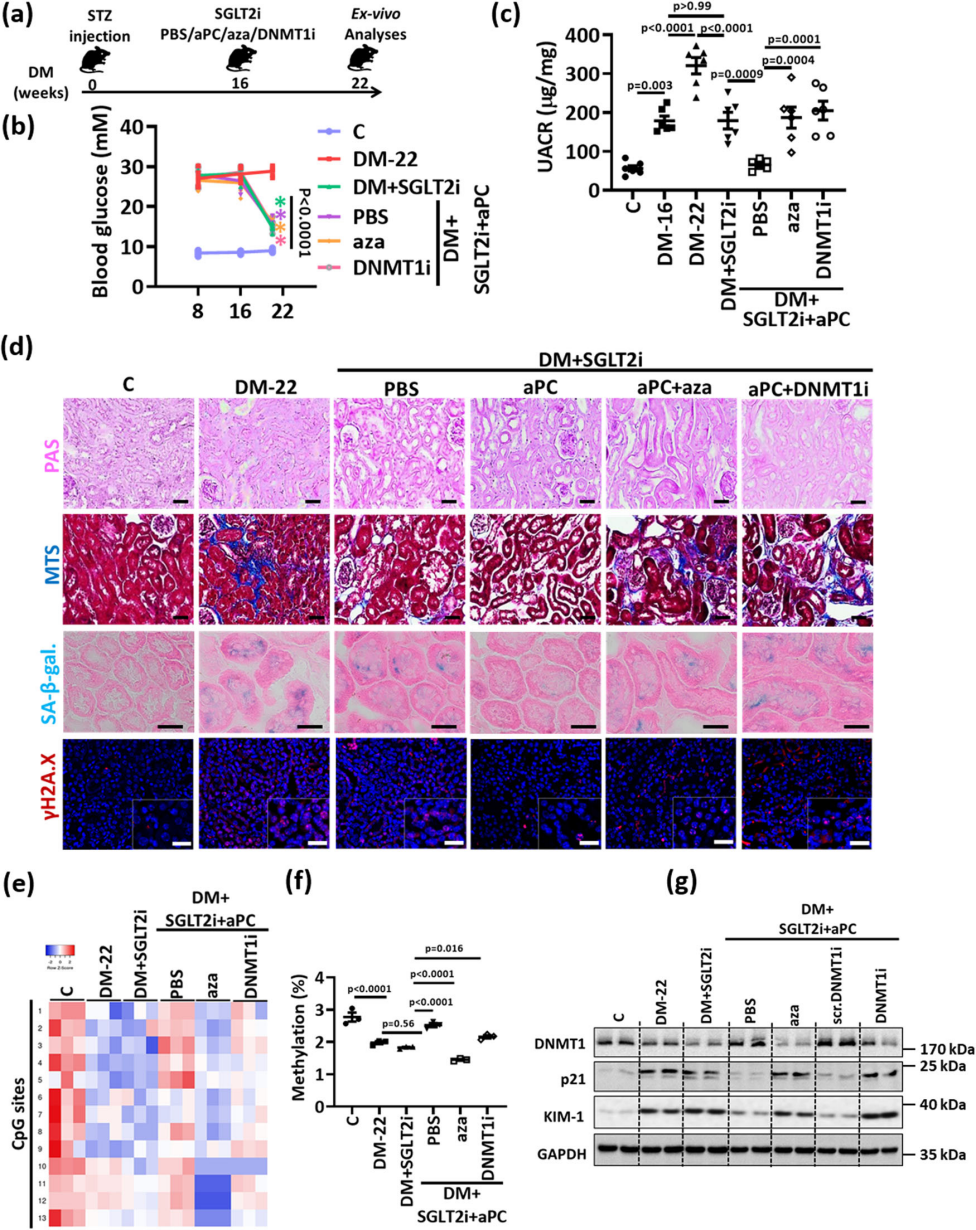
aPC reverses epigenetically sustained renal p21 expression and tubular senescence. **a** Experimental scheme: 16 weeks after induction of persistent hyperglycemia, diabetic mice were treated with PBS (DM-22), sodium/glucose cotransporter 2-inhibitor (Dapagliflozin®, DM + SGLT2i), a combination of SGLT2i and aPC (DM + SGLT2i + aPC), a combination of SGLT2i, aPC and the pan DNMTs-inhibitor 5-aza-deoxycytidine (DM + SGLT2i + aPC+aza), or a combination of SGLT2i, aPC and a vivo morpholino targeting DNMT1 (DM + SGLT2i-aPC-DNMT1i) for further 6 weeks. **b** Average blood glucose levels in experimental groups (as described in a) after 8 or 16 weeks of persistent hyperglycemia and at 22 weeks. **c** Dot plot summarizing albuminuria (urinary albumin-creatinine ratio, µg albumin/mg creatinine; UACR) in experimental groups (as described in **a**). Baseline albuminuria before interventions was determined after 16 weeks of hyperglycemia (DM-16). **d** Exemplary histological images of periodic acid Schiff stains (PAS), interstitial fibrosis (Masson’s trichrome stain, MTS), SA- β-gal. stain (senescence associated β-galactosidase, blue; eosin counterstain), and γ-H2A.X (histone H2A, family X, immunohistochemistry, red; DAPI: nuclear counterstain, insets: larger magnification) in experimental groups (as described in **a**); scale bars represent 20 μm; all scale bars represent 20 μm. **e**, **f** Heat map summarizing methylation status of CpG sites in the p21 promotor region (**e**, the degree of methylation at each CpG site is represented according to the color code.) and dot plot summarizing cumulative quantification CpG islands’ methylation (**f**) in kidneys of experimental groups (as described in **a**). **g** Expression of renal p21, DNMT1 and KIM-1 protein (exemplary immunoblot, **g**; loading control: GAPDH in experimental groups (as described in **a**). A nonspecific scrambled vivo morpholino (scr. DNMT1i) has no effect. Line graph and dot plots reflecting mean ± SEM of 6 mice per group; one-way ANOVA with Sidak’s multiple comparison test. Gels/blots were processed in parallel; source data are provided as a Source Data file-Fig-7.

To analyze whether aPC reverses tubular injury and senescence via DNMT1 in vivo, we reduced blood glucose levels in diabetic mice using SGLT2i starting at week 16 post induction of hyperglycemia for further six weeks (DM + SGLT2i), adding aPC in a subgroup of mice (DM + SGLT2i + aPC+PBS, Fig. 7a, b). In agreement with results shown in Fig. 1, lowering blood glucose levels did not normalize albuminuria (Fig. 7c). Likewise, markers of tubular injury (tubular dilatation, tubulointerstitial fibrosis, increased KIM-1 expression) and tubular senescence (expression of p21, SA-β-gal, γ-H2A.X, Ki-67, Lamin B) as well as reduced DNMT1 expression and p21 promoter demethylation were comparable in SGLT2i-treated (DM + SGLT2i) mice and mice with persistent hyperglycemia (DM-22, Fig. 7d–g, Supplementary Fig. 17). In contrast, treatment of diabetic mice with aPC in addition to SGLT2i from weeks 16 to 22 markedly reduced albuminuria to levels below those observed after 16 weeks of hyperglycemia (DM-16 versus DM + SGLT2i + aPC+PBS, Fig. 7b) and comparable to those in non-diabetic control mice (C versus DM + SGLT2i + aPC+PBS, Fig. 7b). In addition, markers of tubular damage (tubular dilatation, tubulointerstitial fibrosis, KIM-1 expression) and tubular senescence (expression of p21, SA-β-gal, γ-H2A.X, Ki-67, Lamin B) were normalized while DNMT1 expression and p21 promoter methylation were increased by aPC (Fig. 7d–g, Supplementary Fig. 17).

To determine whether aPC controls p21 expression and tubular injury via DNMT1, we chose two complimentary approaches. We either (i) added the pan DNMT inhibitor 5-aza-2’-deoxycytidine (DM + SGLT2i + aPC+aza; 5-aza-2’-deoxycytidine reduces DNMT1 activity and its expression^27^) or (ii) specifically inhibited DNMT1 expression (using vivo-morpholino^28^, DM + SGLT2i + aPC+DNMTi) on top of SGLT2i and aPC treatment in mice with 16 weeks of established hyperglycemia (Fig. 7a). 5-aza-2’-deoxycytidine and DNMT1 vivo morpholino markedly reduced DNMT1 expression (Fig. 7g, Supplementary Fig. 17f). Both interventions abolished aPC’s protective effect, increasing albuminuria levels, tubular dilatation, tubulointerstitial fibrosis, KIM-1 and expression of the senescence associated markers p21, SA-β-gal γ-H2A.X, while Ki-67, Lamin B1 were reduced to levels observed in mice treated with SGLT2i only (Fig. 7c–g, Supplementary Fig. 17). In agreement with an epigenetic regulation, p21 promoter methylation was increased and p21 mRNA levels were suppressed by aPC (DM + SGLT2i + aPC+PBS), but this effect was reversed upon additional treatment with 5-aza-2’-deoxycytidine (DM + SGLT2i + aPC+aza) or DNMT1 vivo morpholino (DM + SGLT2i + aPC+DNMTi, Fig. 7e). A nonspecific scrambled vivo morpholino (Scr-DNMTi) had no effect (Fig. 7, Supplementary Fig. 17). These data establish that aPC controls renal p21 expression via DNMT1.

### aPC regulates epigenetically sustained p21 expression independent of its anticoagulant function

aPC conveys its cytoprotective effect at least partially independent of its anticoagulant properties through receptor-dependent mechanisms, involving typically protease-activated receptors (PARs) and the endothelial protein C receptor (EPCR)^29^. Expression of PAR1, PAR2, PAR4, and EPCR, known to be expressed in an immortalized mouse tubular cell line (BUMPT-cells)^30^, was confirmed in mouse PTCs (Supplementary Fig. 18a). Upon knockdown of PARs or EPCR, we identified PAR1 and EPCR as the receptors required for aPC-mediated reversal of glucose-induced and sustained p21 and KIM-1 expression (Supplementary Fig. 18b, c). The receptor dependent modulation of p21 expression suggests that aPC’s effect on tubular p21 expression is independent of its anticoagulant function.

**Fig. 8:**
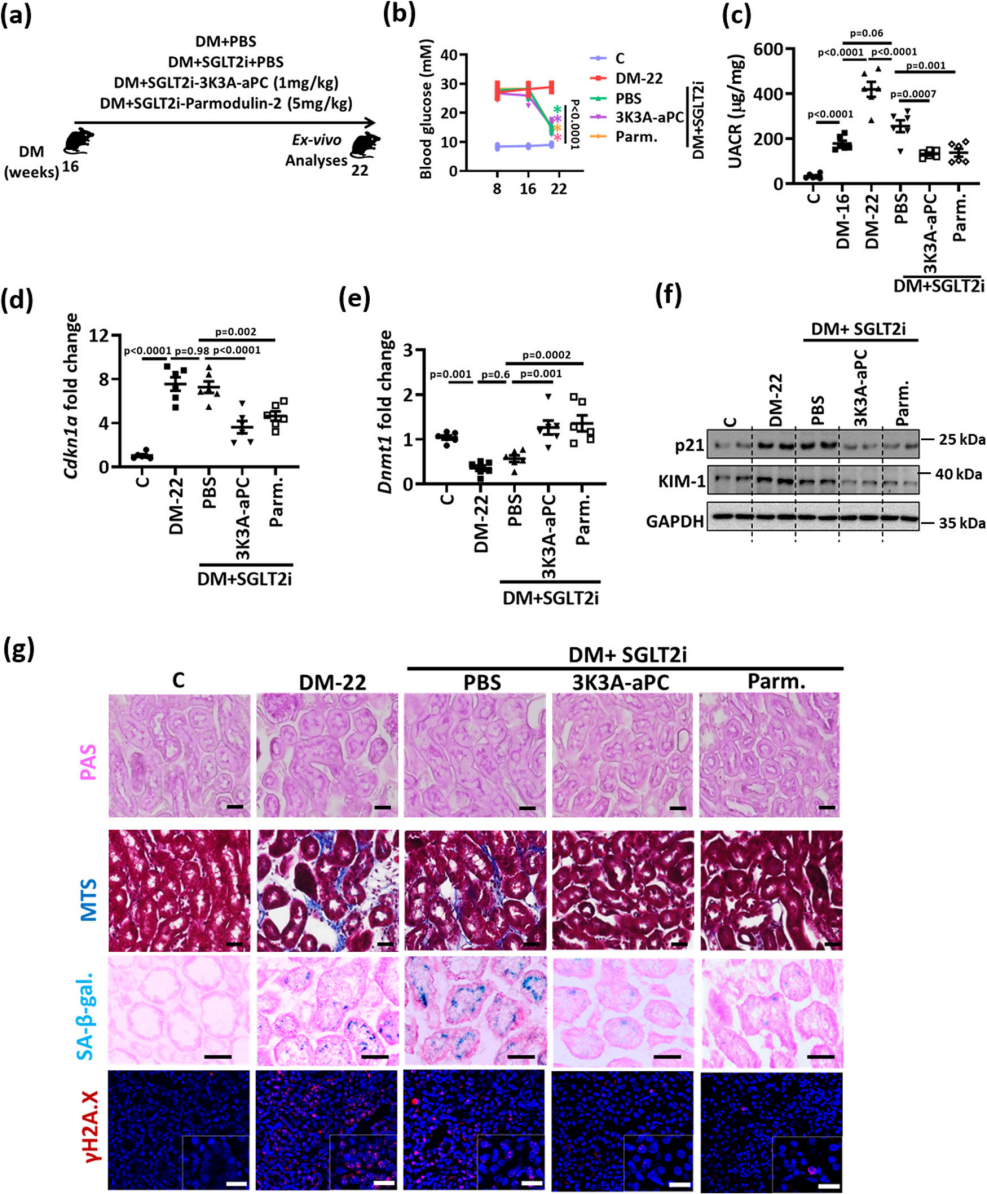
aPC regulates epigenetically sustained p21 expression independent of its anticoagulant function. **a** Experimental scheme: 16 weeks after induction of persistent hyperglycemia, diabetic mice were treated with PBS (DM + PBS), sodium/glucose cotransporter 2-inhibitor (Dapagliflozin®, DM + SGLT2i), a combination of SGLT2i and 3K3A-aPC (DM + SGLT2i + 3K3A-aPC) or a combination of SGLT2i, aPC and parmodulin-2 (DM + SGLT2i + Parm.) for further 6 weeks. **b** Average blood glucose levels after 8 and and16 weeks of persistent hyperglycemia and at 22 weeks in experimental groups (as described in **a**). **c** Dot plot summarizing albuminuria (urinary albumin-creatinine ratio, µg albumin/mg creatinine; UACR) in experimental groups (as described in **a**). Baseline albuminuria before interventions was determined after 16 weeks of hyperglycemia (DM-16). **d**–**f** Expression of renal p21 mRNA (*Cdkn1a*, qRT-PCR, **d**; and protein, **f**), renal DNMT1 mRNA (*Dnmt1*, qRT-PCR, **e**), and renal KIM-1 (protein, **f**) in experimental groups (as described in **a**). Exemplary immunoblots (f; GAPDH: loading control). **g** Exemplary histological images of periodic acid Schiff stain (PAS), interstitial fibrosis (Masson’s trichrome stain, MTS), SA- β-gal. stain (senescence associated β-galactosidase), and γ-H2A.X (histone H2A, family X, red, immunofluorescent, DAPI nuclear counterstain, insets: larger magnification) in experimental groups (as described in **a**); all scale bars represent 20 μm. Line graph or dot plots reflecting mean ± SEM of six mice per group; one-way ANOVA with Sidak’s multiple comparison test. Gels/blots were processed in parallel; source data are provided as a Source Data file-Fig-8.

To determine whether aPC reverses glucose induced p21 expression and associated tubular damage independent of its anticoagulant function in vivo, we used aPC-based pharmacological agents with markedly reduced anticoagulant potential^12^: (i) 3K3A-aPC, an aPC mutant with largely reduced anticoagulant but sustained signaling efficacy and (ii) parmodulin-2, a small peptide mimicking biased aPC signaling via PAR1^31–33^. Again, after 16 weeks of hyperglycemia we initiated treatment with SGLT2i in combination with phosphate-buffered saline (PBS, control), 3K3A-aPC, or parmodulin-2 (Fig. 8a). While blood glucose levels in SGLT2i plus PBS, 3K3A-aPC or parmodulin-2 treated mice were comparable (Fig. 8b), 3K3A-aPC or parmodulin-2 on top of SGLT2i both markedly reduced albuminuria compared to that in SGLT2i + PBS mice (Fig. 8c). While SGLT2i + PBS had no impact on DNMT1 or p21 expression, 3K3A-aPC and parmodulin-2 in addition to SGLT2i increased DNMT1 while reducing p21 expression in renal cortex extracts (Fig. 8d–f, Supplementary Fig. 19a), which was associated with improvement of renal tubular damage (reduced tubular dilatation, interstitial fibrosis, KIM-1 expression) and a normalization of senescence markers (p21, SA-β-gal γ-H2A.X, Ki-67, Lamin B, Fig. 8f, g and Supplementary Fig. 19b–i).

**Fig. 9:**
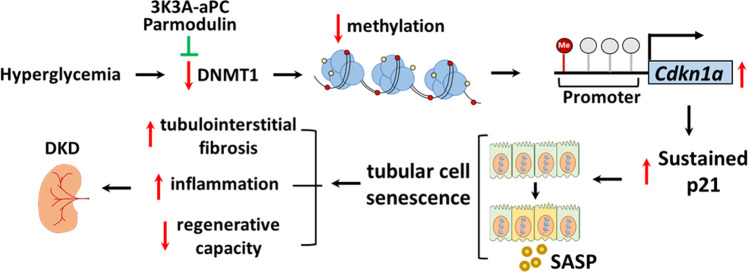
Proposed model summarizing the p21-dependent regulation of the hyperglycemic memory and possible interventions. Hyperglycemia suppresses DNMT1 expression in the tubular compartment, inducing hypomethylation of the p21 promoter, p21 expression, and tubular senescence. The latter is associated with inflammation, impairs the endogenous tubular repair capability and induces tubulointerstitial fibrosis, promoting tubulointerstitial damage and DKD. Exploiting aPC-signaling (Parmodulin, 3K3A-aPC) reverses the epigenetically sustained p21 expression.

Taken together, these data demonstrate that sustained p21 expression drives hyperglycemic memory in the context of DKD. Therapeutic approaches mimicking aPC signaling reduce tubular p21 expression and reverse–at least in mice–hyperglycemic memory (Fig. 9).

## Discussion

We conducted an unbiased expression analysis aiming to define pathways contributing to hyperglycemic memory in DKD, e.g., the persistence of albuminuria despite a marked improvement in blood glucose levels. Approximately half of the hyperglycemia-induced changes in gene expression persisted despite blood glucose reduction, reflecting hyperglycemic memory. The senescence-associated cyclin-dependent kinase inhibitor p21 (*Cdkn1a*), a gene regulating cell proliferation and senescence^34^, was the top hit among genes that were persistently induced. p21 itself is controlled by DNMT1. Restoring DNMT1 and reducing p21 expression by exploiting aPC signaling reverses already established DKD in mice (Fig. 9). This study identified p21-dependent tubular senescence as a pathway contributing to hyperglycemic memory and demonstrates that this mechanism can be therapeutically targeted.

Studies using whole body p21 knock out mice previously established a role of p21 in DKD^35^. Ubiquitous p21 deficiency from birth onwards conveys glomerular and tubular protection in diabetic mice^35^. However, how p21 is regulated in DKD and its role in hyperglycemic memory remained unknown. The role of p21 in tubular senescence in DKD is in agreement with previous observations: (i) the level and duration of p21 induction determine the onset of cellular growth arrest; (ii) p21 expression is sufficient to induce senescence; (iii) p21 expression restricted to tubular cells is sufficient to induce renal fibrosis following acute kidney injury^36,37^; and (iv) p21 induces endoreduplication^38^, which may impair renal tubular cell recovery following acute kidney injury^39,40^. While p21-induced cell-cycle arrest and/or endoreduplication are plausible explanations for impaired renal recovery, the exact mechanisms through which p21-induced senescence prevents renal recovery despite glucose normalization remain to be determined. Yet, our data support a function of sustained p21 expression in the tubular compartment given (i) the cell-autonomous, glucose-dependent induction of p21 in tubular, but not in other renal cell types, (ii) the induction of tubular p21 expression before detectable glomerular injury, (iii) the sustained p21 expression in tubular cells despite glucose lowering, and (iv) the tubular, but not glomerular protection in diabetic TM^Pro/Pro^ x p21^−/−^ mice. However, given intrarenal crosstalk between the glomerular and tubular compartments we currently cannot exclude that persistent glomerular damage contributes to tubular p21 expression. These data support a model in which prolonged and sustained p21 expression causes tubular senescence and compromises tubular repair capacity in DKD patients^41^, even after glycemic control has improved.

The current results suggest that epigenetic regulation of p21 depends on DNMT1. DNMT1 is a regulator of tubular development and nephron progenitor cell renewal during embryonic nephrogenesis^42,43^, supporting a crucial function of DNMT1 in the renal tubular compartment. However, DNMT1 is not expected to specifically target p21 and its reduced expression in DKD is expected to de-repress (hence induce) other genes. Accordingly, about 93% of genes induced in hyperglycemic mice remained elevated despite lowering blood glucose levels, while only about 36% of genes suppressed in hyperglycemic mice remained repressed (Fig. 1d), supporting a concept in which reduced DNMT1 activity results in DNA-demethylation and increased expression of various genes. Analyses of human kidney tissue and peripheral blood monocytes identified hypomethylation and increased expression of other genes previously linked with DKD, such as the integrin β2, genes related to TNF signaling and the redox-regulator TXNIP, corroborating that DNA hypomethylation contributes to the persistent expression of genes linked with diabetic vascular complications^44,45^. Hence, we cannot exclude that other genes regulated by DNMT1 contribute to the hyperglycemic memory.

DNA methylation is a net result of coordinated changes of DNA methylation and demethylation regulators. In addition to p21, expression of Tet2 and Kdm6a, which have been linked with DKD in the past^4,26,46^, remained sustained despite glucose lowering by SGLT2i or insulin. Considering that several pathways mediate cytosine demethylation^47^, the function of DNMT1 to maintain cytosine methylation may be particularly important. Congruently, suppressing DNMT1 expression was sufficient to reduce p21 promoter methylation and induce p21 expression both in vivo and in vitro. However, we cannot exclude that other regulators of DNA-methylation alter cytosine-methylation and gene expression, contributing to the hyperglycemic memory. Likewise, it is possible that not only hypomethylation, but also hypermethylation contributes to the hyperglycemic memory. Yet, the current data do support a crucial function of DNMT1 and p21 for the hyperglycemic memory.

While the current results indicate that sustained p21 expression is linked to reduced DNMT1 expression and p21-promoter demethylation, the mechanism initially inducing p21 expression may be unrelated. DNMT1, p21 and γ-H2A.X are associated with DNA damage response (DDR) and, hence, it is possible that increased tubular ROS generation in DKD induces DNA damage and DDR, inducing p21 expression^48^. Alternatively, p21 expression was increased in diabetic TM^Pro/Pro^ mice (in which aPC-generation is impaired), while aPC suppressed p21 expression in vitro and in vivo. Therefore, endothelial dysfunction with loss of endothelial TM expression and associated reduced aPC generation may both trigger (by regulating ROS)^12^ and maintain (epigenetically, via DNMT1, this study) p21 expression. The question of how tubular p21 expression is induced is addressed in ongoing work.

The regulation of DNMT1 by aPC raises the intriguing possibility that this coagulation protease may epigenetically control other genes. Indeed, in addition to its effect on p21, aPC reverses glucose-induced expression of p66^Shc^, a mitochondrial protein promoting ROS-generation, in podocytes (glomerular cells) and macrophages^12,13^. Furthermore, inhibitors of coagulation factors fIIa (dabigatran) or fXa (rivaroxaban) differentially regulate aPC generation, which is linked to differential gene expression and epigenetic marks^49^. Epigenetic control of gene-expression by aPC provides a rationale for the long-lasting effects of therapeutic aPC applications despite its short half-life^50^ and–considering the broad cytoprotective effects of aPC–may be relevant in other disease settings^29,51^.

The reversal of glucose-induced and sustained p21 or p66^Shc^ expression in tubular cells and in podocytes, respectively, is in agreement with the engagement of different receptor complexes and different mechanisms regulated by aPC in the tubular and glomerular compartments. Thus, aPC signals via a heterodimer of PAR3 with PAR2 (human) or PAR1 (mouse) in conjunction with integrin β_3_ in podocytes, while in tubular cells aPC signals via a heterodimer of PAR1 and EPCR^12,52^ (and current results). Mechanistically, aPC targets the unfolded protein response in glomerular cells^53^ and cellular senescence in tubular cells (this study). These observations support a concept in which different pathomechanisms in the glomerular and tubular compartments promote DKD^54,55^. Importantly, aPC ameliorates both glomerular and tubular damage despite targeting different and cell-specific receptor-complexes and pathways, providing a rationale for its high efficacy in animal models of renal injury^12,52,56^ (and current results).

aPC has shown beneficial effects in a number of acute and chronic disease models^29,50^, suggesting that this coagulation protease is a disease-resolving mediator. However, the therapeutic value of aPC itself is limited by its inherent anticoagulant properties. As previously shown in acute disease models^31,57^ and here in a chronic disease model, harnessing specifically the signaling-dependent disease-resolving effects of aPC is feasible by mimicking its signaling properties. aPC mutants lacking binding sites for the coagulation factor Va (3K3A-aPC) or small molecules mimicking biased signaling of aPC via the G-protein coupled receptor PAR1^31,50^ (parmodulins) are devoid of an anticoagulant function, rendering aPC-based therapies feasible not only in acute, but also chronic diseases.

We used established therapeutic approaches to improve hyperglycemia (SGLT2i and insulin) and observed sustained p21 expression in both cases, supporting the notion that sustained p21 expression reflects the hyperglycemic memory independent of the glucose-lowering intervention and independent of exposure of tubular cells to urinary glucose (which is expected to be increased in SGLT2i-treated, but not insulin-treated mice). Lowering blood glucose levels provided partial protection, as reflected by halted disease progression, which is in agreement with the protective effects of SGLT2 inhibitors in clinical studies^58^. Intriguingly, aPC-based therapeutics provided a benefit on top of SGLT2-inhibtion. Therefore, despite recent data suggesting that SGLT2 inhibitors are useful new therapeutic adjuncts in patients with diabetes, additional therapies for full kidney recovery may be required.

To circumvent the lack of kidney biopsies from diabetic patients before and after improved blood glucose control, we determined urinary p21 levels. p21 was strongly increased in the urine of most diabetic patients and increased with the severity of DKD. Distribution of p21 levels in advanced stages of DKD appeared to be dichotomic, with a small subgroup of patients presenting with low urinary p21 levels despite kidney dysfunction (Fig. 3e). Considering the proposed role of p21 as a mediator and biomarker of the hyperglycemic memory in DKD, urinary p21 levels may allow better stratification of patients with DKD, identifying those patients with advanced kidney injury and an established renal hyperglycemic memory^59^. If confirmed, these patients may not respond to glucose lowering alone, but may profit from a potential therapy targeting tubular senescence.

In summary, we identified a pathway contributing to hyperglycemic memory in the kidney and provided experimental in vivo evidence that targeting hyperglycemic memory in the context of DKD is feasible, potentially by mimicking the cytoprotective effects of aPC (e.g., using small molecules such as parmodulins, pepducins, or aptamers)^31,50,60^.

## Methods

### Study design

The objectives of this study were to identify mechanisms contributing to the hyperglycemic memory in the context of DKD and to test whether this memory could be reversed using suitable therapeutic interventions. We analyzed kidneys from diabetic mice with and without adjusted blood glucose levels using RNAseq, immunostaining, western blotting and quantitative polymerase chain reaction (qPCR). All uncropped blots and gels are provided within the source data. We confirmed our findings in human kidney biopsies and in urine samples from a large cohort of diabetic patients (LIFE-ADULT). Mechanistic studies using gene knockdown and analyses of DNA methylation were conducted in human and mouse renal cells in vitro.

### Mice and in vivo interventions

Wild-type male and female C57BL/6 and p21^*CIP1/WAF1*^ constitutive knock-out (p21^−/−^) mice were obtained from Jackson Laboratory (Bar Harbor, ME, USA). Non diabetic C57BLKsJ-db/+ (db/m) and diabetic C57BL/KSJ-db (db/db) male and female mice were obtained from Janvier (S.A.S., St. Berthevin Cedex, France). TM^Pro/Pro^ mice have been previously described^15,24^. TM^Pro/Pro^ mice were crossed with p21^−/−^ mice, to generate TM^Pro/Pro^ x p21^−/−^ double mutant mice. In some mice hyperglycemia was induced in 8 weeks old mice using low dose streptozotocin (STZ) injection following established protocols (intraperitoneally, 60 mg/kg body weight, freshly dissolved in 0.05 mol/l sterile sodium citrate, pH 4.5 for 5 consecutive days)^12,52^. Age-matched control mice received sodium-citrate. Blood and tissue samples were collected after 22 weeks of persistent hyperglycemia^12,15,52^. Subsets of mice were treated with a sodium-glucose co-transporter 2 inhibitor (SGLT-2i, Dapagliflozin®, 25 mg/kg body weight, in the drinking water^61^, with the activated protein C (aPC, 1 mg/kg body weight, every other day, intraperitoneally)^12,53^, with insulin glargine (Lantus®, 1-2 IU, every other day, subcutaneously), with 5-aza-2’-deoxycytidine (aza, 0,1 mg/kg body weight, every other day, intraperitoneally)^13^, an aPC variant lacking specifically anticoagulant function (3K3A-aPC; 1 mg/kg body weight, every other day, intraperitoneally)^31^, the biased PAR1 agonist parmodulin-2 (ML161, Parm, 5 mg/kg body weight, every other day, intraperitoneally)^31,33^, DNMT1 vivo morpholino (DNMTi, targeting the translation of transcript variant 1 of mouse DNMT1; 6 mg/kg body weight in PBS, every other day, intraperitoneally)^62^, or DNMT1 mismatch vivo morpholino (Scr.DNMTi). Control mice received PBS every other day (intraperitoneally). All interventions were conducted for six weeks, starting after 16 weeks of persistent hyperglycemia. Albuminuria indicative of DKD was ensured before initiating treatment. All animal experiments were conducted following standards and procedures approved by the local Animal Care and Use Committee (Landesverwaltungsamt Halle and Leipzig, Germany).

### Cell lines and primary cells

Human embryonic kidney cells (HEK-293) were routinely grown and maintained according to supplier’s instructions (ATCC, USA, Cat No: CRL-1573). Mouse primary proximal tubular cells (PTC) were isolated and cultured according to established protocols^30^. Immortalized Boston University mouse proximal tubular cells (BUMPT) were obtained from Dr. Wilfred Lieberthal (Boston University) and cultured as described previously^30^. Please refer to supplementary methods for further details.

### Human renal biopsies and urine samples

Human renal biopsy samples were provided by the tissue bank of the National Center for Tumor Diseases (NCT, Heidelberg, Germany). Human urine samples obtained from the local outpatient clinic were obtained based on informed consent (Ethic vote no: 082-10-19042010, University of Leipzig). All participants of the LIFE-ADULT cohort (Ethic vote no: 263-2009-14122009 and 201/17-ek, University of Leipzig)^21^ were fasted on average for 12.8 ± 1.9 h. LIFE-ADULT participants were compensated with a travel allowance. Urine samples from the FMD study (DRKS00014287) and HEIST-DiC observational study were obtained after ethical approval (Ethic vote no: 383/2016, 204/2004 and 682/2016, Ruprecht-Karls-University of Heidelberg) and informed consent. For the FMD study, the primary and secondary outcomes are not a part of this study and have been separately published^23^. The participants did not receive any compensation. Patients receiving de novo a SGLT2 inhibitor were identified in the diabetes outpatient clinic. Please see Supplementary Tables 1–5 for patient characteristics.

### Statistical analysis

The data are summarized as mean ± SEM (standard error of the mean). Statistical analyses were performed with parametric Student’s *t*-test and ANOVA or non-parametric Mann–Whitney-Test and Kruskal–Wallis test, as appropriate, and post-hoc comparison with the method of Tukey, Bonferroni or Sidak’s multiple comparison. The Kolmogorov–Smirnov (KS) test or D’Agostino–Pearson-Normality-test was used to determine whether the data are consistent with a Gaussian distribution. Prism 9 (www.graphpad.com) software was used for statistical analyses. Statistical significance was accepted at values of *p* < 0.05.

### Reporting summary

Further information on research design is available in the Nature Research Reporting Summary linked to this article.

## Supplementary information

Supplementary Information File

Reporting Summary

## Data Availability

The sequencing data generated in this study have been deposited in the GEO data repository database under accession code GSE199929. The raw data are available as source data. The source data generated in this study are provided in the Supplementary Information/Source Data file. Databases used within the study: Heatmapper (heatmapper.ca) was used to generate heat maps. pantherDB (http://www.pantherdb.org/) was used for pathway analysis. Source data are provided with this paper.

## Source data

Source data
