# Supplementary material for: Reversal of the renal hyperglycemic memory in diabetic kidney disease by targeting sustained tubular p21 expression
Source: Nat Commun. 2022 Aug 27;13:5062. doi: 10.1038/s41467-022-32477-9 (PMC9420151; doi:10.1038/s41467-022-32477-9)
Supplement: Supplementary file 1 — Supplementary Information File [file 41467_2022_32477_MOESM1_ESM.pdf]

Supplementary Materials for

Title: Reversal of the renal hyperglycemic memory in diabetic kidney disease by targeting sustained tubular p21 expression

Authors: Moh'd Mohanad Al-Dabet[1,2]†, Khurrum Shahzad[1,3]†, Ahmed Elwakiel[1]†, Alba Sulaj[4], Stefan Kopf[4], Fabian Bock[1,5], Ihsan Gadi[1], Silke Zimmermann[1], Rajiv Rana[1], Shruthi Krishnan[1], Dheerendra Gupta[1], Jayakumar Manoharan[1], Sameen Fatima[1], Sumra Nazir[1], Constantin Schwab[6], Ronny Baber[1,7], Markus Scholz[8], Robert Geffers[9], Peter Rene Mertens[10], Peter P. Nawroth[4], John Griffin[11], Maria Keller[12,13], Chris Dockendorff[14], Shrey Kohli[1]*, Berend Isermann[1]*

Affiliations:
[1] Institute of Laboratory Medicine, Clinical Chemistry and Molecular Diagnostics, Universitätsklinikum Leipzig, Leipzig University, Leipzig, Germany.
[2] Department of Medical Laboratories, Faculty of Health Sciences, American University of Madaba (AUM), Amman, Jordan.
[3] Department of Biotechnology, University of Sargodha, Pakistan.
[4] Internal Medicine I and Clinical Chemistry, German Diabetes Center (DZD), University of Heidelberg, Heidelberg, Germany.
[5] Department of Medicine, Vanderbilt University Medical Center, Nashville, Tennessee, USA.
[6] Institute of Pathology, University of Heidelberg, Heidelberg, Germany.
[7] Leipzig Medical Biobank, Leipzig University, Leipzig, Germany.
[8] Institute for Medical Informatics, Statistics and Epidemiology, Leipzig University, Leipzig, Germany.
[9]Helmholtz Centre for Infection Research, Braunschweig, Germany.
[10] Clinic of Nephrology and Hypertension, Diabetes and Endocrinology, Otto-von-Guericke University, Magdeburg, Germany.
[11] Department of Molecular Medicine, The Scripps Research Institute, La Jolla, CA, USA.
[12] Helmholtz Institute for Metabolic, Obesity and Vascular Research (HI-MAG) of the Helmholtz Center Munich at the University of Leipzig and University Hospital Leipzig, Leipzig, Germany.
[13] Medical Department III – Endocrinology, Nephrology, Rheumatology, University of Leipzig Medical Center, Leipzig, Germany.
[14] Department of Chemistry, Marquette University, Milwaukee, WI, USA.

†These authors contributed equally to the work.

*Corresponding author:
Berend Isermann, MD
Institute of Laboratory Medicine, Clinical Chemistry and Molecular Diagnostics
Leipzig University
Liebigstr. 27A, 04103 Leipzig, Germany.
Phone: +49 – (0)341-97-22200
Fax: +49 – (0)341-97-22379
E-mail: berend.isermann@medizin.uni-leipzig.de

Inventory of supporting information

Supplementary Methods

Supplementary Tables

Table S1 (corresponding to Fig. 3a-c).

Table S2 (corresponding to Fig. 3e).

Table S3 (corresponding to Fig. 3f-j).

Table S4 (corresponding to Fig. 3k,l).

Table S5 (corresponding to Fig. 3m,n).

Table S6

Table S7

Supplementary Figures

Supp. Fig. 1

Supp. Fig. 2

Supp. Fig. 3

Supp. Fig. 4

Supp. Fig. 5

Supp. fig. 6

Supp. Fig. 7

Supp. Fig. 8

Supp. Fig. 9

Supp. Fig. 10

Supp. Fig. 11

Supp. Fig. 12

Supp. Fig. 13

Supp. Fig. 14

Supp. Fig. 15

Supp. Fig. 16

Supp. Fig. 17

Supp. Fig. 18

Supp. Fig. 19

Uncropped Blots for supplementary figures

Supplementary references

Supplementary Methods

Reagents

The following antibodies were used in the current study:

Antibody	Company	Cat. no.	Clone	Dilution
rabbit anti-p21	Santa Cruz Biotechnology	sc-397	C-19	1:400
rabbit anti-megalin	Santa Cruz Biotechnology	sc-25470	H-245	1:100
mouse-anti synaptopodin	Santa Cruz Biotechnology	sc-515842	D-9	1:400
rabbit anti-DNMT1	Cell Signaling Technology	5032	D63A6	1:1000
rabbit anti-human p21	Cell Signaling Technology	2947	12D1	1:1000
rabbit anti-γ-H2A.X	Cell Signaling Technology	9718	20E3	1:1000
rabbit anti-Ki-67	Cell Signaling Technology	9129	D3B5	1:350
rabbit anti-Lamin B1	Cell Signaling Technology	17416	E6M5T	1:500
rabbit anti-β-actin	Cell Signaling Technology	4970	13E5	1:2000
anti-rabbit IgG, HRP-linked	Cell Signaling Technology	7074	NA	1:4000
rabbit anti-KIM-1	Abcam	ab47635	NA	1:2000
rabbit anti-mouse p21	Abcam	ab188224	EPR18021	1:800
rabbit anti-GAPDH	Sigma-Aldrich	G9545	NA	1:25000
goat anti-mouse nephrin	R&D systems	AF3159	NA	1:300
anti-mouse/rabbit IgG polymer, HRP-linked	R&D systems	VC002-025	NA	direct
rabbit anti-WT-1	Abnova	MAB20854	CEH-23	1:350
anti-rabbit IgG, texas red linked	Vector Laboratories	TI-1000	NA	1:400
anti-goat IgG, fluorescein linked	Vector Laboratories	FI-5000	NA	1:400

Other reagents: Mouse DNMT1 siRNA (sc-35203), Dapagliflozin® (Santa Cruz Biotechnology, Germany); Dulbecco′s Modified Eagle′s Medium (DMEM), DMEM/F-12, RPMI 1640, Ham's F-12 nutrient mixture, trypsin-EDTA, penicillin/streptomycin solution, fetal bovine serum (FBS), ITS supplement, D(+)-Glucose, type I collagenase, RNA*later*™, Hank's Balanced Salt Solution (HBSS) and HEPES buffer (PAA Laboratories, Austria); donkey serum, horse serum, antibiotic/antimycotic solution, APO transferrin and hydrocortisone solution (Sigma-Aldrich, Germany); recombinant human epidermal growth factor (R&D systems, USA); IFN-γ (Cell Sciences, Germany); BCA reagent (Perbio Science, Germany); Vectashield mounting medium with DAPI and citrate-based antigen-unmasking solution (citrate-based, Vector Laboratories, USA); PVDF membrane and immobilon TM western chemiluminescent HRP substrate (Millipore, United States); ammonium persulphate (Merck, Germany); streptozotocin (Enzo Life Sciences, Germany); insulin glargine (Lantus®, Sanofi, France); accu-chek test strips, accu-check glucometer and protease inhibitor cocktail (Roche Diagnostics, Germany); albumin fraction V, hematoxylin Gill II, acrylamide, agarose, periodic acid-Schiff (PAS) reagent, aqueous Eosin, Histokitt synthetic mounting medium and

dimethylsulfoxide (DMSO, Carl ROTH, Germany); aqueous mounting medium (ZYTOMED, Germany); Trizol Reagent, soyabean trypsin inhibitor and PBS (Life Technologies, Germany); mouse DNMT1 vivo morpholino and DNMT1 mismatch vivo morpholino (Gene Tools, USA); TurboFect (Thermo Fisher Scientific, Germany).

Kits used in this study: RevertAid™ H minus first strand cDNA synthesis kit, Dynabeads® mRNA DIRECT™ micro purification kit (Thermo Fisher Scientific, Germany); QIAamp DNA mini kit, RNeasy mini kit, PyroMark PCR kit, PyroMark Gold Q24 Reagents, EpiTect MSP kit (Qiagen, Germany); EZ DNA Methylation kit (Zymo Research, Germany), human p21 matched antibody pair kit (abcam, USA); mouse albumin quantification ELISA kit (Bethyl Laboratories, Germany); Trichrome stain (Masson) kit and senescence cells histochemical staining kit (Sigma-Aldrich, Germany); DAB substrate kit (Vector Laboratories, USA); EpiQuik DNA methyltransferase activity/inhibition assay kit (Epigentek, USA); ScriptSeq v2 RNA-Seq library preparation kit (Epicentre Technologies, USA), TruSeq SBS Kit v3-HS (Illumina, USA).

Mice and interventions:

Mice had been backcrossed onto the C57BL/6 background for at least 9 generations and were routinely maintained on the C57BL/6 background. Age matched littermates were randomly assigned to intervention or control groups. For diabetes model, Blood glucose and body weight were measured once weekly [1,2]. On average 85 - 90% of STZ-injected mice became diabetic (blood glucose >17 mmol/l) within the first 4 weeks and these were included as diabetic mice in the experiments. Mice that did not develop persistently elevated blood glucose levels and maintained blood glucose levels <11 mmol/l despite STZ-injections were included in the control group [1]. The endpoints analysed did not differ between STZ-injected, but normoglycaemic mice and sodium-citrated injected mice (controls).

Cell culture and *in vitro* interventions

HEK-293 cells (ATCC, USA, Cat No: CRL-1573) were routinely grown and maintained in DMEM low glucose (5.5 mM glucose concentration) medium in the presence of 10% FBS at 37°C. BUMPT cells were grown in DMEM low glucose in the presence of 10% FBS and maintained at 33°C in the presence of interferon γ (INF-γ, 10 U/ml) to enhance the expression of a thermosensitive T-antigen. Under these conditions BUMPT cells remain proliferative. To induce the differentiation, BUMPT cells were grown for 2 days at 37°C in the same medium without INF-γ. Differentiation was ascertained by expression of megalin, a marker of proximal tubular cells. Only differentiated cells were used for further experiments. Immortalized mouse mesangial cells (MES13) were grown in a 3:1 mixture of DMEM low glucose and Ham's F12 nutrient mixture with 14 mM HEPES and 10% FBS according to supplier’s instructions (ATCC, USA, Cat No: CRL-1927) at 37°C. Primary mouse glomerular endothelial cells (Cell Biologics, Cat No.: C57-6014G) were grown in M1168 mouse endothelial cell medium with growth factors, as provided by the manufacturer (Cell Biologics, USA) at 37°C. Conditionally immortalized mouse podocytes (obtained from Prof. Jochen Reiser, USA) were grown on collagen type I in RPMI 1640 medium at 33°C in the presence of IFN-γ (10 U/mL) to enhance expression of the thermosensitive T antigen. Under these conditions, the cells proliferate and are undifferentiated. To induce differentiation, podocytes were grown at 37 °C in the absence

of IFN-γ for 14 days. Experiments were performed 14 days after the induction of differentiation. Differentiation was confirmed by examining the expression of synaptopodin.

The *in vitro* model of hyperglycemic memory was performed according to an established protocol [3] with minor modifications. PTCs or early passages of HEK-293 cells were maintained in high concentration of glucose (25 mM) for 24 h followed by glucose normalization (5.5 mM) for another 24 h (*via* media changing). In pre aPC-treatment condition (HG(aPC)/NG) aPC (20 nM) was added to the high glucose medium, whereas in post aPC-treatment (HG/NG(aPC) aPC (20 nM) was added upon returning cells to normal glucose (5.5 mM glucose).
In a subset of experiments, BUMPT cells were maintained in high concentration of glucose (25 mM) for 48 h with or without SGLT2i (Dapagliflozin®, 2 µM). Other cells were maintained in high concentration of glucose (25 mM) for 24 h followed by glucose normalization (5.5 mM) for another 24 h (*via* media changing).

Glucose uptake assay

The fluorescent glucose derivative 2-(N-(7-Nitrobenz-2-oxa-1,3-diazol-4-yl) Amino)-2-Deoxyglucose (2-NBDG, Cayman) was used to determine cellular glucose uptake. BUMPT cells were seeded at a density of $1x10^4$/well in 96-well plates and were maintained in culture medium under normal glucose (5.5 mM) or high glucose (25 mM) concentration for 24 h. For glucose uptake assay, the culture medium was removed from each well and the cells were rinsed three times with HBSS. Then, the cells were incubated for 2 h at 37°C in the presence of 2-NBDG (200 µM) dissolved in HBSS with or without SGLT2i (Dapagliflozin®, 2 µM) and with normal (5.5 mM) or high (25 mM) glucose concentration. The uptake reaction was stopped by removing the incubation buffer and washing the cells three times with HBSS. 2-NBDG fluorescence was measured using Cytation 5 multi-mode plate reader (BioTek) at excitation/emission maxima of 475/550 nm, respectively.

In vitro Knockdown

Knockdown of protease activated receptors (PARs) or endothelial protein C receptor (EPCR) in PTCs was achieved by lentiviral transduction of short hairpin RNA (shRNA) constructs (pGIPZ-F2r, pGIPZ-F2rl1, pGIPZ-F2rl3, and pGIPZ-Procr, obtained from Dharmacon, Lafayette, CO, USA; targeting mouse PAR1: 5'-ATA TAA GAA GTG ACA TCC A- ; mouse PAR2: 5'-TTG AGC TGA AGA GTA GGA GC- , mouse PAR4: 5'-AAA CAG AGT CCA GTA GTG AGG- ; and mouse EPCR: 5'-AAA TTC CTG CAG TTC ATA CCG- , respectively). A scrambled non-silencing RNA (5'-TAC GAG TAT GAT GTT GGT GGG-) was transduced into PTCs as control. Lentiviral particles were generated and concentrated from HEK-293T cells [4]. HEK-293T cells were transduced with pGIPZ-F2r, pGIPZ-F2rl1, pGIPZ-F2rl3 or pGIPZ-Procr together with the packaging plasmid psPAX2 and VSV-G expressing plasmid (pMD2.g; Addgene, USA). The lentiviral particles were harvested from the supernatant 36 and 48 h post-transduction. The lentiviral supernatant was concentrated by mixing virus-containing medium with 50% PEG6000 solution, 4 M sodium chloride and 1 x PBS at 26%, 11%, 12% by volume and incubated on a shaker at 4°C for 3-4 h. Following incubation, the solution was aliquoted into 50 ml falcons and centrifuged at the 4700 g at 4°C for 30 min. After centrifugation, the supernatant was carefully removed, and the tube was placed on tissue paper

for 1 minute. Cell culture media was added to the semi-dried tube for re-suspension, and then the tube was placed in the 4°C fridge with a cover for overnight recovery. The enriched lentivirus was harvested and aliquoted before being snap frozen.

Knockdown of DNMT1 was achieved by transfection of small interfering RNA (siRNA) against mouse DNMT1 or by the use of vivo morpholino targeting the translation of transcript variant 1 of mouse DNMT1. For the siRNA knockdown, PTC were plated at a density of $2.5x10^5$/well in a 6-well plate with complete medium. At a confluency of 80-90%, the supernatants were removed, and the cells were supplemented with fresh medium and transfected with mouse DNMT1 siRNA (Santa Cruz Biotechnology, Germany) using TurboFect transfection reagent (Thermo Fisher Scientific, Germany) according to the manufacturer's instructions.

For vivo morpholino knockdown, PTC or BUMPT cells were plated at a density of $2.5x10^5$/well in a 6-well plate with complete medium. At a confluency of 80-90%, the supernatants were removed, and the cells were supplemented with fresh medium containing varying concentrations of vivo morpholino (5, 10 or 20 µM) for 24 h. In a subset of experiments, BUMPT cells were treated with high glucose (25 mM) for additional 24 h after vivo morpholino treatment (10 µM) for 24 h. Knockdown efficiency in all experiments was confirmed by immunoblotting.

Preparation of activated protein C

Activated protein C (aPC) was generated according to an established protocol[5]. Prothrombin complex (Prothromplex NF600), containing all vitamin K dependent coagulation factors, was reconstituted with sterile water and supplemented with CaCl2 at a final concentration of 20 mM. The column for purification of protein C was equilibrated at RT with 1 liter of washing buffer (0.1 M NaCl, 20 mM Tris, pH 7.5, 5 mM benzamidine HCl, 2 mM Ca2+, 0.02% sodium azide). The reconstituted prothombin complex was gravity eluted on a column filled with Affigel-10 resin covalently linked to a calcium-dependent monoclonal antibody to PC (HPC4). The column was washed first with two column volumes of washing buffer and then two column volumes with a wash buffer rich in salt (0.5 M NaCl, 20 mM Tris, pH 7.5, 5 mM benzamidine HCl, 2 mM Ca2+, 0.02% sodium azide). Then the benzamidine was washed off the column with a buffer of 0.1 M NaCl, 20 mM Tris pH 7.5, 2 mM Ca2+, 0.02% sodium azide. To elute PC the column was gravity eluted with elution buffer (0.1 M NaCl, 20 mMTris, pH 7.5, 5 mM EDTA, 0.02% sodium azide, pH 7.5) and 3 ml fractions were collected. The peak fractions were identified by measuring absorbance at 280 nm. The peak fractions were pooled. The recovered PC was activated with human plasma thrombin (5% w/w, 3 h at 37°C). To isolate activated protein C (aPC) ion exchange chromatography with FPLC (ÄKTAFPLC®, GE Healthcare Life Sciences) was used. First, thrombin was removed with a cation exchange column MonoS (GE Healthcare Life Sciences). Then a MonoQ anion exchange column (GE Healthcare Life Sciences) was equilibrated with 10% of a 20 mM Tris, pH 7.5, 1 M NaCl buffer. After applying the solution that contains aPC a 10-100% gradient of a 20 mM Tris, pH 7.5, 1 M NaCl buffer was run through the column to elute aPC at a flow of 1-2 ml/min under continuous monitoring of OD and conductivity. APC eluted at ~36 mS cm-1 by conductivity or at 40% of the buffer. Fractions of 0.5 ml were collected during the peak and pooled. Proteolytic activity of purified aPC was ascertained with the chromogenic substrate SPECTROZYME® PCa and purity was ascertained by a Coomassie stained gel and immunoblotting.

Urine collection and processing

Mouse urine was collected in metabolic cages at indicated time points. Mice were individually placed in metabolic cages to collect 12 h urine samples. Mouse urine was used for determination of albumin and creatinine.

Urine samples from the LIFE-ADULT cohort were centrifuged at 2750 g for 10 min within 2h and stored at –80°C by the LIFE-Biobank team, part of the Leipzig Medical Biobank. Other urine samples were centrifuged at 5000 g for 5 min to remove debris and dead cells. Human urine supernatants were used for determination of p21 by immunoblotting or ELISA (see next section). For p21 immunoblotting, aliquots of the human urine supernatant were centrifuged again at 20000 g for 60 min and the supernatant was mixed with laemmli sample buffer containing protease inhibitors.

p21 ELISA for human urine samples

For determination of urinary p21 by ELISA we used anti-human p21 matched antibody pair Kit (abcam, USA) according to manufacturer’s instructions. Maxisorb plates were coated with p21 capture antibody overnight at 4°C followed by washing and blocking at 37°C for 1 h. Human urine supernatants were diluted (1:2), added to the microplates and incubated for 2 h at room temperature on a plate shaker set to 400 rpm. This was followed by washing (3x, using wash buffer) and incubation with detector antibody for 1 h at room temperature on a plate shaker at 400 rpm. Following washing (3x, using wash buffer), HRP-Streptavidin solution was added and the plate was incubated for 1 h at room temperature on a plate shaker at 400 rpm. Plates were washed (3x using wash buffer) and tetramethylbenzidine (TMB) substrate was added for color development. Reaction was stopped by adding stop solution (as provided in the kit) and absorbance was measured at 450 nm after 10 min.

Albuminuria in mouse urine samples

Mouse urine albumin and creatinine were measured using established protocols [1,4]. Urine albumin was determined using a mouse albumin ELISA kit (Mouse albumin ELISA quantification kit, Bethyl Laboratories) according to the manufacturer’s instructions. Urine creatinine was determined using a commercially available assay (Roche Diagnostics, Cobas c501 module) in the Institute of Clinical Chemistry and Pathobiochemistry, Medical Faculty, Otto-von-Guericke University, Magdeburg, Germany and institute of laboratory medicine, clinical chemistry and molecular diagnostics, Leipzig, Germany[5].

Methylation specific PCR (MSP)

Methylation-specific PCR was performed using commercially available assays [2,6]. Genomic DNA was extracted from HEK-293 cells or kidney tissues using the QIAamp DNA Mini kit (QIAGEN) and treated with sodium bisulfite using the EZ DNA Methylation kit (Zymo Research) according to the manufacturer’s instructions. PCR amplification was performed with the EpiTect MSP kit (QIAGEN) using primer pairs designed to specifically detect either methylated or unmethylated CpG sites in the human or mouse p21 promoter: human methylated forward, 5′-TTT GTT GTA TGA TTT GAG TTA G-3′; human methylated reverse, 5′-TAA TCC CTC ACT AAA TCA CCT C-3′; human unmethylated forward, 5′-ATC ATT CTG GCC TCA AGA TGC-3′; and human unmethylated reverse, 5′-CGG CTC CAC AAG GAA CTG AC-3′;

mouse methylated forward 5′- GTT AGC GAG TTT TCG GGA TC-3′; mouse methylated reverse 5′-CTC GAC TAC TAC AAT TAA CGT CGA A-3′; mouse unmethylated forward 5′-GGT TAG TGA GTT TTT GGG ATT G-3′; mouse unmethylated reverse 5′-TCT CAA CTA CTA CAA TTA ACA TCA AA-3′. For ratio analyses, amplicons against methylated and unmethylated CpGs were visualized on a 2% (w/v) agarose gel. Universal methylated human and mouse DNA (Millipore) was used as a positive control.

Pyrosequencing

Bisulfite-converted DNA was used to amplify the differentially methylated sites within the promoter region of mouse *Cdkn1a* (chr17:29,093,442-29,093,823) using PyroMark CpG custom assay primers (PMC00110489) and PyroMark PCR kit (Qiagen; **supplementary table**-7). The product was further subjected to pyrosequencing on a PyroMark Q24 and using PyroMark Q24 software, version 2.0.6 (Qiagen), a total of 13 CpG sites were screened. The percentage of methylation at every CpG site was determined as well as the mean methylation level across all 13 CpG sites.

DNMT activity assay

DNMTs activity was measured by EpiQuik DNA Methyltransferase Activity/Inhibition assay kit (Epigentek) according to the manufacturer's instructions. Confluent HEK-293 cells were treated with a cytoplasmic extraction buffer (10 mM HEPES, 1.5 mM $MgCl_2$, 10 mM KCl and 0.5 mM DTT), briefly vortexed and then centrifuged (14000 g, 4°C, 30 sec). Supernatants containing the cytoplasmic protein fractions were discarded. The pellet was re-suspended in 100 µl of a nuclear extraction buffer (20 mM HEPES, 420 mM NaCl, 1.5 mM $MgCl_2$, 0.2 mM EDTA, 0.5 mM DTT and 25% glycerol), incubated for 20 min on ice, followed by centrifugation (13.000 g, 4°C, 5 min). Supernatants containing DNMTs within the nuclear extracts were collected and added to wells coated with cytosine-rich DNA-substrate and pre-incubated with the DNMT assay buffer and the methyl group donor Adomet. Wells were covered and incubated for 1 h at 37°C. Wells were then washed three times and the capture antibody against 5-methylcytosine was added. After 1 h incubation on an orbital shaker at room temperature wells were washed 4 times, the secondary detection antibody was added, and samples were incubated for 30 min at room temperature. Wells were washed five times and developer solution was added for 5 min before adding the stop solution. Absorbance was immediately measured at 450 nm.

Immunoblotting

Cell lysates were prepared using RIPA buffer containing 50 mM Tris (pH7.4), 1% NP-40, 0.25% sodium-deoxycholate, 150 mM NaCl, 1 mM EDTA, 1 mM Na_3VO_4, 1 mM NaF supplemented with protease inhibitor cocktail. Lysates were centrifuged (13000 g for 10 min at 4°C) and insoluble debris was discarded. Protein concentration in supernatants was quantified using BCA reagent. Equal amounts of protein were electrophoretically separated on 7.5%, 10% or 12.5% SDS polyacrylamide gel, transferred to PVDF membranes and probed with desired primary antibodies at appropriate dilution (KIM-1: 1:2000; SC-p21: 1:350; CST-p21: 1:1000; DNMT1: 1:1000; GAPDH: 1:25000; β-actin: 1:1000). After overnight incubation with primary antibodies at 4°C membranes were washed with TBST and incubated with anti-rabbit IgG (1:2000) horseradish peroxidase-conjugated antibody for 1 hr at room temperature. Blots were

developed with the enhanced chemiluminescence system. To compare and quantify levels of proteins, the density of each band was measured using ImageJ software. Equal loading for total cell or tissue lysates was determined by GAPDH or β-actin immunoblots using the same blot whenever possible.

Reverse transcriptase PCR (RT-PCR)

Kidney tissues stored in RNA*later*™ (Invitrogen) were thawed on ice and transferred directly into Trizol (Life Technologies) for isolation of total RNA following the manufacturer's protocol. For *in viro* cells, Trizol was added to the cells after removing the medium and washing with PBS. Quality of total RNA was ensured on agarose gel and by analysis of the A260/280 ratio. The reverse transcription reaction was conducted using 1 µg of total RNA after treatment with DNase I (5 U/5 µg RNA, 30 min, 37°C) followed by reverse transcription using RevertAid First Strand cDNA Synthesis Kit (Thermo Fisher Scientific). Semi-quantitative polymerase chain reactions were performed and gene expression was normalized to β-actin. PCR products were separated on a 1.5 – 1.8% agarose gel and visualized by ethidium bromide (Et-Br) staining. Reactions lacking reverse transcription served as negative controls. For primers sequence see **supplementary table 6**.

Quantitative real time PCR (qRT-PCR)

Total RNA was extracted from kidney tissues or PTC. RNA quality was ensured, and cDNA was generated as outlined in the previous section. qRT-PCR was carried out on CFX Connect Real-time system (Bio-Rad Laboratories, Hercules, CA) using SYBR Green (Takyon™, Eurogentec). For quantitative analysis results were normalized to 18srRNA or to β-actin. Gene expression was analyzed using the $2^{-(\Delta\Delta Ct)}$ method. Reactions lacking cDNA served as negative controls. For primers sequence see **supplementary table 6**.

RNA expression profiling

RNA was isolated from murine renal cortex using RNAeasy mini kit (QIAGEN). RNA was isolated from non-diabetic, age matched control mice (C, C57Bl/6), from mice with 22 weeks of persistent hyperglycemic following STZ injection as outlined above (DM), or from mice with 16 weeks of persistent hyperglycemia followed by 6 additional weeks with lowered blood glucose levels using SGLT2i (DM+SGLT2i). In another set of experiments, we isolated RNA from aged matched (22 weeks age) normoglycemic control mice (db/m), hyperglycemic diabetic mice (db/db), and db/db mice with initially elevated, but then markedly reduced blood glucose levels for 6 weeks prior to analyses (db/db+SGLT2i). Quality and integrity of total RNA was verified using an Agilent Technologies 2100 Bioanalyzer (Agilent Technologies; Waldbronn, Germany).

The Dynabeads® mRNA DIRECT™ Micro Purification Kit (Thermo Fisher) was used for purification of mRNA. The RNA sequencing library was generated from 500ng total RNA using Dynabeads® mRNA DIRECT(tm) Micro Purification Kit (Thermo Fisher) for mRNA purification followed by NEBNext® Ultra(tm) II Directional RNA Library Prep Kit (New England BioLabs) according to manufacture´s protocols. The libraries were sequenced on Illumina NovaSeq 6000 using NovaSeq 6000 S2 Reagent Kit (100 cycles, paired end run) with an average of 50 mio reads per RNA sample. Each FASTQ file gets a quality report generated by FASTQC tool (v0.11.8). Before alignment to reference genome (mm10, transcriptome:

Mus_musculus.GRCm38.79.gtf) each sequence in the raw FASTQ files were trimmed on base call quality and sequencing adapter contamination using Trim Galore! wrapper tool (version 0.4.4) . Reads shorter than 20 bp were removed from FASTQ file. Trimmed reads were aligned to the reference genome using open source short read aligner STAR 2.5.2b (https://code.google.com/p/rna-star/) with settings according to log file. Further statistical analysis were performed using R Project for Statistical Computing version 4.1.0 (2021-05-18). Feature counts were determined using R package "Rsubread 2.6.1". Only genes showing counts greater 5 at least two times across all samples were considered for further analysis (data cleansing). Gene annotation was done by R package "bioMaRt. Before starting the statistical analysis steps, expression data was log2 transform and TMM normalized ("edgeR 3.34.0"). Differential gene expression was calculated by R package "edgeR 3.34.0". . The statistical analysis was performed on the filtered and annotated data with Limma package (moderated t statistics). To correct for multiple testing error, the Benjamini-Hochberg correction (BH) was used.

Functional annotation and Pathway analysis

The threshold to identify differentially expressed genes (DEGs) was set to a logFc value of ±0.58 resulting in 1.5-fold expression change. Statistically significant DEGs ($p<0.01$; $FDR<0.1$) were categorized into genes with persistently changed expression despite glucose lowering and genes with normalized expression upon glucose lowering. Heatmapper (heatmapper.ca) was used to generate heatmaps of gene expression data. Row dendrograms were generated using hierarchical row clustering calculated by squared Euclidean distances. pantherDB (http://www.pantherdb.org/) was used for pathway analysis. The percentage of gene hits relative to total number of pathway hits was used to evaluate prominent pathways involved.

Histology, immunohistochemistry and histological analyses

Tubular morphometrics were determined using a histological score based on periodic acid Schiff's (PAS) stained images, following an established protocol [7]. At least 50 glomerular and tubular cross sections were analyzed for each mouse. For determination of the tubular and glomerular parameters adjacent sections were compared to identify the maximal glomerular diameter and to ensure that a transversal tubular cross-sections were analyzed and to avoid artefacts from tangentially cut glomeruli or tubuli. To calculate the epithelial surface area of tubular cross sections, the difference of the area of external tubuli profile and the area of the tubular lumen was determined [7]. Tubular cell hyperplasia was evaluated on the same tubular cross sections by counting the number of nuclei[7]. Tubular cell hypertrophy was calculated as the ratio between epithelial surface and the number of nuclei for tubular section [7].

Renal tubulointerstitial fibrosis was evaluated using Masson's trichrome staining (MTS) [4]. Ten visual fields per slide were randomly selected at 200 x magnification. Interstitial fibrosis was quantified using NIH-ImageJ software, generating a binary image allowing automatic calculation of the stained area as percentage of the image area [4]. The glomerular fractional mesangial area (FMA) was calculated following the current DCC (Diabetes Complications Consortium) protocol [4]. In brief, 5 μm thick sections were stained with PAS reagent. In every investigated glomerulus, the tuft glomerular area was determined using NIH-ImageJ software. FMA was calculated as the fractional area positive for PAS in the glomerular tuft area, reported as the percentage.

p21, KIM-1 and WT-1 expression were determined by immunohistochemistry (IHC) following established protocols [2]. The number of p21 or WT-1 positive cells (defined as those stained brown using a DAB substrate, 3, -diaminobenzidin; hematoxylin counterstain) was determined in at least 10 randomly chosen fields per section at a magnification of 40 x in the renal cortical area.

Images for all histological analyses were obtained using an Olympus BX43 microscope, Olympus XC30 Camera, and Olympus cellSens Dimension 1.5 Image software. All images of a specific analytical method (e.g. FMA, IHC-stains) were taken with the same settings. The evaluations were performed by a blinded investigator.

Immunofluorescence

Immunofluorescence staining of γH2A.X, Ki-67, Lamin B1, nephrin and synaptopodin was performed on paraffin embedded kidney sections or PTC grown on cover slips [8]. For paraffin sections, after rehydration and antigen retrieval, the sections were incubated with a blocking buffer (2.5% donkey serum in PBS) followed by overnight incubation with primary antibodies. For cover slips, after fixation in 3.7% paraformaldehyde, the cover slips were blocked (1% BSA in PBS) followed by overnight incubation with primary antibodies. Sections or coverslips were washed then incubated with secondary antibodies for 2 h followed by further washing and mounting with Vectashield mounting medium containing the nuclear stain DAPI. Images were acquired using Leica THUNDER imaging microscope (DMi8 platform; Leica, Germany). Images were analyzed using Leica Application Suite X (LAS X) software 8, Leica, Germany). Fluorescence intensities were analyzed using image-J software according to the method of corrected total cell fluorescence (CTCF) as outlined previously [9].

Senescence associated beta galactosidase (SA-β-gal.) staining

Renal cell senescence was evaluated using Senescence Cells Histochemical Staining Kit (CS0030-Sigma) according to the manufacturer's protocol. 5 µm thick cryro-sections of renal tissue or early passage HEK-293 cells were washed in 1 x PBS and fixed for 1 min (2% formaldehyde, 0.2% glutaraldehyde, 7.04 mM Na_2HPO_4, 1.47 mM KH_2PO_4, 0.137 M NaCl, 2.68 mM KCl) and then rinsed in ice-cold 1 x PBS for 5 min. The cryo-sections were incubated with freshly prepared X-gal staining solution (10 ml containing 0.25 ml of 40 mg/ml X-gal Solution, 125 µl of 400 mM potassium ferricyanide, 125 µl of 400 mM potassium ferrocyanide) at 37°C overnight. Next, excess staining solution was washed away using ice-cold 1 x PBS, and the sections were overlaid with 70% glycerol followed by staining assessment. For frozen sections, aqueous Eosin was used for counterstaining before mounting the slides with 70% glycerol. The development of blue dots/areas was counted as positive staining. Senescence was evaluated as the ratio (in percent) of the blue-colored area in each sample relative to the total area of at least 10 renal cortical visual fields per section.

Supplementary Tables

**Characteristic**	**Control (n=6)**	**DM (-DKD) (n=6)**	**DM (+DKD) (n=5)**
**Age** (years)	71.5 ± 3.1	75.0 ± 3.2	78.2 ± 2.5
**Sex** (female/male)	(3/3)	(3/3)	(2/3)
**History (N) of**			
Hypertension	2	4	4
Brain tumor	1	-	-
COPD	1	-	-
Steatosis hepatitis	1	-	-
peripheral arterial occlusive disease	1	-	-
Hypothyroidism	1	-	-
Type 2 diabetes	-	6	5
**Diabetic complications (N)**			
Diabetic neuropathy	-	2	-
Diabetic kidney disease (DKD)	-	-	5
**Serum creatinine** (mg/dl)	1.1 ± 0.17	1.1 ± 0.06	1.9 ± 0.36
**Serum urea** (mg/dl)	6.6 ± 0.66	6.8 ± 0.69	10.8 ± 1.78

Table S1 (corresponding to Fig. 3a-c). Anthropometric, clinical and metabolic characteristics of renal biopsy control and diabetic patients with and without diabetic kidney disease (+/- DKD). Data represents mean ± SEM. Abbreviations: DKD: Diabetic Kidney Disease, DM: Diabetes Mellitus, COPD: chronic obstructive pulmonary disease.

Characteristic	Control (n=22)	DKD (n=26)	oCKD (n=18)
**Age** (years)	64.2 ± 2.1	65 ± 2.6 n.s.	61,9 ± 3,7 n.s.
**Sex** (female/male)	(8/14)	(5/21)	(6/12)
**Diabetes complication**			-
Nephropathy (n)	-	26	
Retinopathy (n)	-	3	
Neuropathy (n)	-	10	
**History of**			
Hypertension (n)	7	18	7
Coronary heart disease	0	12	5
Myocardial infarction (n)	0	4	4
OSAS (n)	3	3	0
Arthrosis (n)	5	4	1
Thyroid nodules (n)	0	1	0
Thyreoidectomy (n)	2	0	1
**Diabetes therapy**			
Metformin (n)	-	5	-
DPP-4 (n)	-	4	-
SGLT2 inhibitor (n)	-	1	-
Glinide (n)	-	0	-
Long-acting insulin	-	18	-
**Other medication**			
RAAS-inhibitors (n)	5	12	9
Beta-blockers (n)	2	15	6
Thiazide diuretics (n)	0	2	3
Loop diuretics (n)	2	13	2
Calcium-antagonist (n)	1	5	0
Statin (n)	2	17	2
Ezetimib (n)	0	4	2
Acetyl-salicylic acid (n)	0	7	3
L-Thyroxin	3	3	2
**Glycemic control**			
HbA1c (%)	5.7 ± 0.2	7.5 ± 0.2**	5,6 ± 0,13 n.s.
RBG (mmol/l)	6.1± 0.8	9.9 ± 1.1*	5,2 ± 0,4 n.s.
**Serum creatinine** (mg/dl)	0.86 ± 0.17	1.67 ± 0.71***	2,28 ± 0,7***
**eGFR** (ml/min/1.73m²)	77,6 ± 3,5	43,9 ± 5,6***	41,2 ± 5,2***
**Albuminuria** (mg albumin/g urine-creatinine)	7,2 ± 0,8	537,4 ± 152,9**	511,2 ± 147,9***

Table S2 (corresponding to Fig. 3e). Anthropometric, clinical and metabolic characteristics of non-diabetic controls, diabetic patients with known DKD, and patients with other causes of CKD (oCKD) recruited from the local outpatient clinic. Data represents mean ± SEM, comparison by *t*-test (DKD or oCKD against control group), *P<0.05, ** P<0.01, *** P<0.001 and n.s: non-significant. Abbreviations: DPP-4: dipeptydil peptidase 4, SGLT2: sodium-glucose cotransporter 2, RAAS: renin-angiotensin-aldosterone system, RBG: random blood glucose, eGFR: estimated glomerular filtration rate.

Characteristic	Control	Low risk	Moderate risk	High risk	Very high risk
**Number of patients**	**n=36**	**n=52**	**n=53**	**n=29**	**n=18**
**Age** (years)	(63.2 ± 0.4)	(71.0 ± 0.6)***	(70.1 ± 0.7)***	(71.0 ± 0.9)***	(74.1 ± 1.1)***
**Sex** (female/male)	(15/22)	(7/45)	(15/38)	(11/18)	(5/13)
**BMI** (kg/m²)	28.38±0.76	30.1±4.6	30.7±4.5*	30.6±4.3*	31.2±4.3*
**Blood pressure**					
systolic (mmHg)	135.28 ± 2.91	138.8 ± 19.2	131.8 ± 18.3	134.1 ± 19.8	147.6 ± 32.4
diastolic (mmHg)	76.92 ± 1.73	73.9 ± 9.2	71.1 ± 8.9**	70.6 ± 8.6**	77.7 ± 14.0
**Diabetes therapy (N)**					
Metformin	-	28	37	18	5
DPP-4	-	8	17	7	6
Glinide	-	6	7	7	4
Long-acting insulin	-	8	14	11	12
**Other medication (N)**					
RAAS-inhibitors	6	37	41	20	18
Beta-blockers	5	23	27	15	12
Thiazide diuretics	0	8	0	0	0
Loop diuretics	0	2	0	0	0
Calcium-antagonist	0	9	19	20	6
Statin	1	27	23	14	7
Ezetimib	0	2	0	0	0
Acetyl-salicylic acid	1	17	0	0	0
L-Thyroxin	3	7	11	3	1
**Glycemic control**					
HbA1c (%)	5.40 ± 0.10	6.1 ± 0.7***	6.4 ± 0.7***	6.2 ± 1.2**	6.0 ± 0.6**
**Serum creatinine** (mg/dl)	(0.99 ± 0.10)	(0.92 ± 0.02)	(0.95 ± 0.02)	(1.05 ± 0.05)	(1.50 ± 0.07)***
**eGFR** (ml/min/1.73m²)	(99.4 ± 0.4)	(79.8 ± 1.3)***	(68.9 ± 1.4)**	(73.9 ± 3.2)***	(40.9 ± 3.0)***
**Albuminuria** (mg albumin/g urine-creatinine)	(8.8 ± 1.12)	(11.1 ± 1.0) n.s.	(85.4 ± 10.4)***	(730 ± 203.8)***	(802 ± 176.2)***

Table S3 (corresponding to Fig. 3f-j). Anthropometric, clinical and metabolic characteristics of non-diabetic controls and type-2 diabetic individuals from the LIFE-adult cohort. The severity of DKD was classified according to the KDIGO criteria as low, moderate, high and very high risk of chronic kidney disease (CKD). Data represents mean ± SEM, comparison by *t*-test (each diabetic group against controls), *P<0.05, ** P<0.01, *** P<0.001 and n.s: non-significant. Abbreviations: BMI: body mass index, DPP-4: dipeptydil peptidase 4, RAAS: renin-angiotensin-aldosterone system, eGFR: estimated glomerular filtration rate.

Characteristic	Baseline (n=10)	after SGLT2i (n=10)
**Age** (years)	60.9 ± 3.2	--
**Sex** (female/male)	1/9	--
**Duration post SGLT2i (months)**	--	7.2 ± 0.8
**Diabetes therapy (n)**		
Metformin	7	7
DPP-4	2	3
SGLT2 inhibitors	10	10
Glinide	0	0
Long-acting insulin	3	3
**Other medication (n)**		
RAAS-inhibitors	7	7
Beta-blockers	6	6
Thiazide diuretics	1	1
Loop diuretics	2	2
Calcium-antagonist	2	2
Statin	4	4
Ezetimib	0	0
Acetyl-salicylic acid	6	6
L-Thyroxin	2	2
**Glycemic control**		
HbA1c (%)	7.8 ± 0.3	6.9 ± 0.3*
FBG (mg/dl)	175.4 ± 12.21	133.0 ± 9.25*
**eGFR** (ml/min/1.73m²)	96.9 ± 3.9	99.0 ± 2.9 n.s
**Albuminuria** (mg albumin/g urine-creatinine)	17.6 ± 6.9	17.6 ± 7.4 n.s

Table S4 (corresponding to Fig. 3k,l). Anthropometric, clinical and metabolic characteristics of diabetic patients before and after start of SGLT2i treatment (Empagliflozin) recruited from the HEIST-DiC study. Data represents mean ± SEM, comparison by paired *t*-test, **P*<0.05, n.s: non-significant. Abbreviations: DPP-4: dipeptydil peptidase 4, SGLT2: sodium-glucose cotransporter 2, RAAS: renin-angiotensin-aldosterone system, eGFR: estimated glomerular filtration rate.

**Characteristic**	**Baseline (n=10)**	**After 3 cycles FMD (n=10)**
**Age** (years)	64.8 ± 2.4	--
**Sex** (female/male)	4/10	--
**BMI** (kg/m²)	30.4 ± 1.6	28.5 ± 1.6
**Diabetes duration** (years)	15.0 ± 2.2	--
**Diabetes complication (n)**		
Nephropathy	10	8
Retinopathy	2	2
Neuropathy	4	4
**History (n) of**		
Hypertension	10	10
Coronary heart disease	2	2
Myocardial infarction	1	1
OSAS	1	1
Arthrosis	4	4
Thyroid nodules	2	2
Thyreoidectomy	2	2
**Diabetes therapy (n)**		
Metformin	9	9
DPP-4	4	3
SGLT2 inhibitors	3	1
Glinide	1	0
Long-acting insulin	1	1
**Other medication (n)**		
RAAS-inhibitors	9	9
Beta-blockers	5	5
Thiazide diuretics	4	4
Loop diuretics	3	3
Calcium-antagonist	3	4
Statin	3	3
Ezetimib	2	2
Acetyl-salicylic acid	2	3
**Glycemic control**		
HbA1c (%)	7.4 ± 0.3	6.9 ± 0.3**
FBG (mM)	8.6 ± 0.7	7.8 ± 1.1
**Blood pressure** (systolic/diastolic mmHg)	138.6/83.3 ± 3.8/3.0	137.4/80.7 ± 3.3/2.7
**Serum creatinine** (mg/dl)	0.9 ± 0.1	0.9 ± 0.1
**eGFR** (ml/min/1.73m²)	93.1 ± 7.1	96.9 ± 7.7
**eGFR from cystatin C** (ml/min/1.73m²)	88.2 ± 5.9	86.8 ± 5.4
**Total cholesterol** (mg/dl)	184.2 ± 12.9	167.0 ± 13.4
**LDL** (mg/dl)	101.5 ± 12.7	97.2 ± 13.4
**HDL** (mg/dl)	45.4 ± 3.2	44.5 ± 3.7
**Triglycerides** (mg/dl)	245.5 ± 77.3	125.7 ± 20.7
**Albuminuria** (mg albumin/g urine-creatinine)	62.8 ± 16.2	47.9 ± 8.7

Table S5 (corresponding to Fig. 3m,n). Anthropometric, clinical and metabolic characteristics of diabetic patients before and after 3 cycles of FMD (fasting mimicking

diet). Data represents mean ± SEM, comparison by paired *t*-test, $^{**}P<0.01$. Abbreviations: BMI: body mass index, OSAS: obstructive sleep apnea syndrome, DPP-4: dipeptydil peptidase 4, SGLT2: sodium-glucose cotransporter 2, RAAS: renin-angiotensin-aldosterone system, FPG: fasting plasma glucose, eGFR: estimated glomerular filtration rate, LDL: low-density Lipoprotein, HDL: high-density lipoprotein. The study is registered in the German Clinical Trials Register (DRKS00014287).

Target	Sequence (5’-3’)
DNMT1 vivo morpholino	CAGGTTGCAGACGACAGAACAGCTC
Scr. morpholino	CAGCTTCCAGACCACACAACACCTC
Mt-hp21 (taken from Bott et al [10])	**F:** TTTGTTGTATGATTTGAGTTAG
	**R:** TAATCCCTCACTAAATCACCTC
Um-hp21 (taken from Bott et al [10])	**F:** ATCATTCTGGCCTCAAGATGC
	**R:** CGGCTCCACAAGGAACTGAC
hDNMT1	**F:** GGCGGCTCAAAGATTTGGAAAGAG
	**R:** CACCGTTCTCCAAGGACAAATC
hDNMT3a	**F:** CTTTGATGGGATTGCTACAGG
	**R:** ACACCTCGGAGGCAATGTAG
hDNMT3b	**F:** CAGGGAAAACTGCAAAGCTC
	**R:** ATTTGTTACGTCGTGGCTCC
hp21	**F:** CCGAAGTCAGTTCCTTGTGG
	**R:** CATGGGTTCTGACGGACAT
hβ-actin	**F:** GCCTCGCCTTTGCCGAT
	**R:** CCACGATGGAGGGGAAGAC
Mt-mp21	**F:** ATATTAGTGATTTGGAAAAGAGTTAGT
	**R:** CTCCAATTCCCCTAAACTCTAAC
Um-mp21	**F:**ATATCAGTGATCTGGAAAAGAGTTAGT
	**R:** CTCCAATTCCCCTAGACTCTGAC
mp21	**F:** TCCACAGCGATATCCAGACA
	**R:** GGACATCACCAGGATTGGAC
mDNMT1	**F:** CAGAGACTCCCGAGGACAGA
	**R:** TTTACGTGTCGTTTTTCGTCTC
mDNMT3a	**F:** CGTGAGTCCGGTGTGTCA
	**R:** CTCCAACCACACACACAAGG
mDNMT3b	**F:** TTCAGTGACCAGTCCTCAGACACGAA
	**R:** TCAGAAGGCTGGAGACCTCCCTCTT
mKIM-1	**F:** TCCACACATGTACCAACATCAA
	**R:** GTCACAGTGCCATTCCAGTC
mPAR1	**F:** CCAGCCAGAATCAGAGAGGA
	**R:** CGGAGATGAAGGGAGGAG
mPAR2	**F:** CCAGGAAGAAGGCAAACATC
	**R:** TGTCCCCCACCAATACCTC
mPAR3	**F:** CATCCTGCTGTTTGTGGTTG
	**R:** TACCCAGTTGTTGCCATTGA
mPAR4	**F:** GCAGACCTTCCGATTAGCTG
	**R:** CACTGCCGAGAACAGTACCA
mEPCR	**F:** CTACAACCGGACTCGGTATGAA
	**R:** CCAGGACCAGTGATGTGTAAGA
mβ-actin	**F:** CTAGACTTCGAGCAGGAGATGG
	**R:** GCTAGGAGCCAGAGCAGTAATC
18srRNA	**F:** GGCCCTGTAATTGGAATGAGTC
	**R:** CCAAGATCCAACTACGAGCTT
mIl-1b	**F:** CCACCTTTTGACAGTGATGAGA
	**R:** GACAGCCCAGGTCAAAGGTT
mIl-6	**F:** CGTGGAAATGAGAAAAGAGTTGT
	**R:** GGTAGCATCCATCATTTCTTTGT

mIl-10	**F:** CGGGAAGACAATAACTGCACCC
	**R:** CGGTTAGCAGTATGTTGTCCAGC
mCcl-8	**F:** CATGGAAGCTGTGGTTTTCCAGA
	**R:** CCATGTACTCACTGACCCACTTC
mCcl-11	**F:** TCCATCCCAACTTCCTGCTGCT
	**R:** CTCTTTGCCCAACCTGGTCTTG
mCxcl-12	**F:** GGCGGTCAAAAAGTTTGCCTT
	**R:** CAGTTAGCCTTGCCTTTGTTCAG
mCxcl-13	**F:** CATAGATCGGATTCAAGTTACGCC
	**R:** GTAACCATTTGGCACGAGGATTC
mIcam-1	**F:** AGTCCGCTGTGCTTTGAGA
	**R:** CACACTCTCCGGAAACGAATACA
mMcp-1	**F:** ACCTGCTGCTACTCATTCACC
	**R:** GAGCTTGGTGACAAAAACTACAG
mMmp-3	**F:** CTCCACAGACTTGTCCCGTT
	**R:** ATGCTGTGGGAGTTCCATAGAG
mTnf-a	**F:** GGTGCCTATGTCTCAGCCTCTT
	**R:** GCCATAGAACTGATGAGAGGGAG
mGrem-2	**F:** CTCGCCTTACAAGGATGGTAGC
	**R:** AGGTACTTGCGCTCGGTGACTA
mCdkn2a	**F:** TGTTGAGGCTAGAGAGGATCTTG
	**R:** CGAATCTGCACCGTAGTTGAGC
mCdkn2b	**F:** GGGCAAGTGGAGACGGTG
	**R:** GCCCATCATCATGACCTGGATT
mCdkn2d	**F:** AAGACGGCCTTGCAGGTCA
	**R:** GCATCTTGGACATTGGGGCT
mTet2	**F:** GCTCCAGTAGGACTGAGAAGGGA
	**R:** GCTGCGGTTGTGCTGTCATT
mTet3	**F:** TTGGGGACACCCTCTACCAG
	**R:** GCACAGGTCCGGTCATCATT
mKdm3a	**F:** ACACTTAGGCAAAAGCACTGG
	**R:** GAAGGGCTCCTCCCTTTCA
mKdm6a	**F:** CATAACCGCACAAACCTGACC
	**R:** GACCTTTGTGAAGCCCCTGA

Table S6: Sequences for vivo morpholinos and Primers used in the current study. Abbreviations: h: human; m: mouse; Mt: methylated; Um: unmethylated; F: forward; R: reverse.

Primer	Sequence (5’-3’)
Forward primer	ATATTAGTGATTTGGAAAAGAGTTAGT
Reverse primer (biotinylated)	CTCCAATTCCCCTAAACTCTAAC
Sequencing Primer	ATTGTTGTTTATAGATGTATGTG

Table S7: Sequences of primers used for pyrosequencing of p21 promotor.

Supplementary Figures for

Title: Reversal of the renal hyperglycemic memory in diabetic kidney disease by targeting sustained tubular p21 expression

Supp. Fig. 1 (related to Fig. 1)

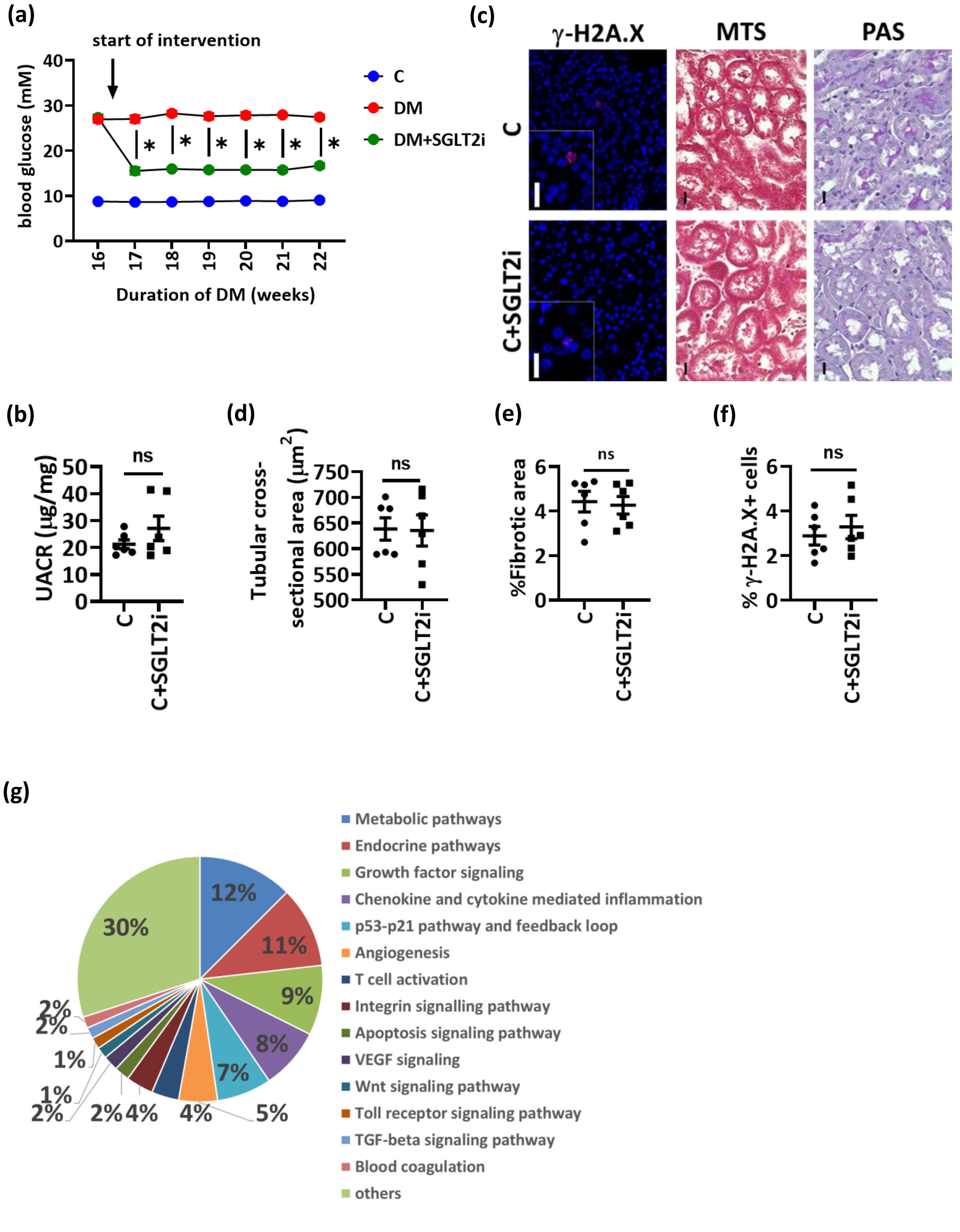

Supp. Fig. 1: Sustained p21 expression in a model of type 1 DM despite reduced blood glucose levels with SGLT2 inhibition

a) Line graph summarizing the weekly average blood glucose levels in the experimental groups: non-diabetic controls (C) and diabetic mice (DM, STZ-model). 16 weeks after induction of persistent hyperglycemia, diabetic mice were treated with PBS (DM) or sodium/glucose cotransporter 2-inhibitor (Dapagliflozin®, SGLT2i; DM+SGLT2i) for further 6 weeks. Line graphs reflecting mean±SEM of 6 mice per group; two-way ANOVA with Sidak's multiple comparison test, *P<0.0001.

b) Dot plot summarizing albuminuria (urinary albumin-creatinine ratio, µg albumin/mg creatinine; UACR) in wild type non-diabetic mice (C) compared to non-diabetic mice treated for 6 weeks with SGLT2i (Dapagliflozin®, C+SGLT2i). Dot plot reflecting mean±SEMof 6 mice per group; unpaired t-test, ns: non-significant. P=0.25.

c) Exemplary histological images of periodic acid Schiff staining (PAS), interstitial fibrosis (Masson's trichrome stain, MTS), and γ-H2A.X stainings in experimental groups (as described in b). γ-H2A.X is immunofluorescently detected, red; DAPI nuclear counterstain, blue; insets: larger magnification; all scale bars represent 20 µm.

d-f) Dot plots summarizing tubular cross-sectional area **(d)**, percentage of fibrotic area, **(e)** and γ-H2A.X positive cells, **(f)** in experimental groups (as described in **b**). Dot plot reflecting mean±SEM of 6 mice per group; unpaired t-test, ns: non-significant, P=0.94 **(d)**, P=0.79 **(e)**, P=0.56 **(f)**.

g) Pie chart reflecting renal pathways identified based on genes persistently changed despite lowering blood glucose in type 1 diabetic mice.

Supp. Fig. 2 (related to Fig. 2)

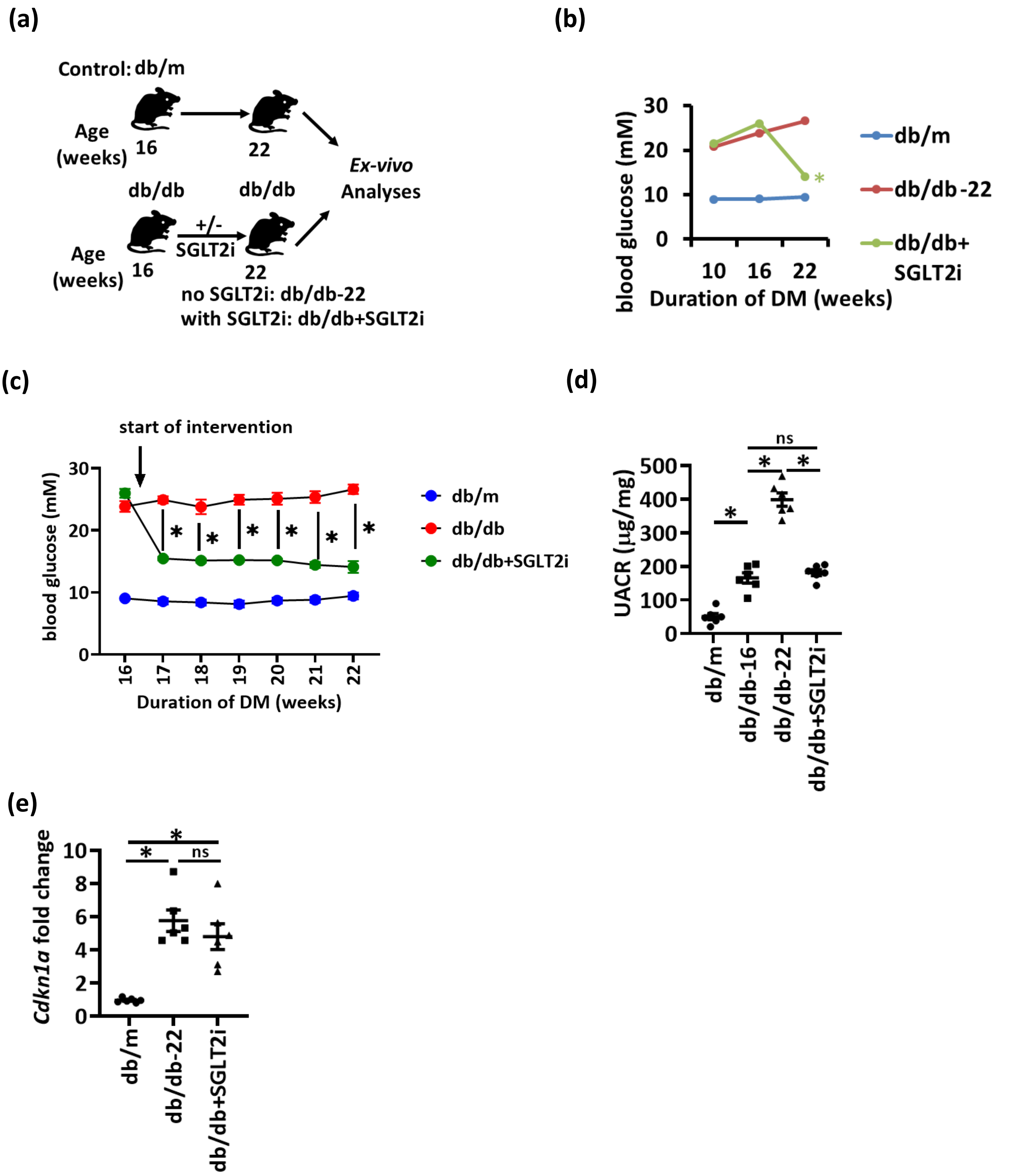

Supp. Fig. 2: Sustained p21 expression despite reduced blood glucose levels in a model of type 2 DM

a) Experimental scheme of type 2 diabetes mellitus model (T2DM): 16 weeks old db/dbmice were treated with PBS (db/db-22) or with the sodium/glucose cotransporter 2-inhibitor (Dapagliflozin®, SGLT2i, db/db+SGLT2i) for further 6 weeks. Non-diabetic mice (db/m) were used as controls.

b) Average blood glucose levels in experimental groups (T2DM-model, as described in a) after 10 or 16 weeks of hyperglycemia and at 22 weeks. Line graphs reflecting mean±SEM of 6 mice per group; one-way ANOVA with Sidak's multiple comparison test, *P<0.0001.

c) Line graph summarizing the weekly average blood glucose levels in the experimental groups (as described in a) from week 16 to week 22. Intervention was started at 16 weeks of age. Line graphs reflecting mean±SEM of 6 mice per group; two-way ANOVA with Sidak's multiple comparison test, *P<0.0001.

d) Dot plot summarizing albuminuria (urinary albumin-creatinine ratio, µg albumin/mg creatinine; UACR) in experimental groups (as described in a). Dot plot reflecting mean±SEM of 6 mice per group; one-way ANOVA with Sidak's multiple comparison test, *P<0.05; ns: non-significant. P<0.0001 (db/m vs db/db-16), P<0.0001 (db/db-16 vs db/db-22), P<0.0001 (db/db-22 vs db/db+SGLT2i), P=0.9 (db/db-16 vs db/db+SGLT2i).

e) Dot plot summarizing renal p21 mRNA (*Cdkn1a*, qRT-PCR) in experimental groups (as described in a). Dot plot reflecting mean±SEM of 6 mice per group; one-way ANOVA with Sidak's multiple comparison test, *P<0.05; ns: non-significant. P=0.0001 (db/m vs db/db-22), P=0.6 (db/db-22 vs db/db+SGLT2i), P=0.001 (db/m vs db/db+SGLT2i).

Supp. Fig. 3 (related to Fig. 2)

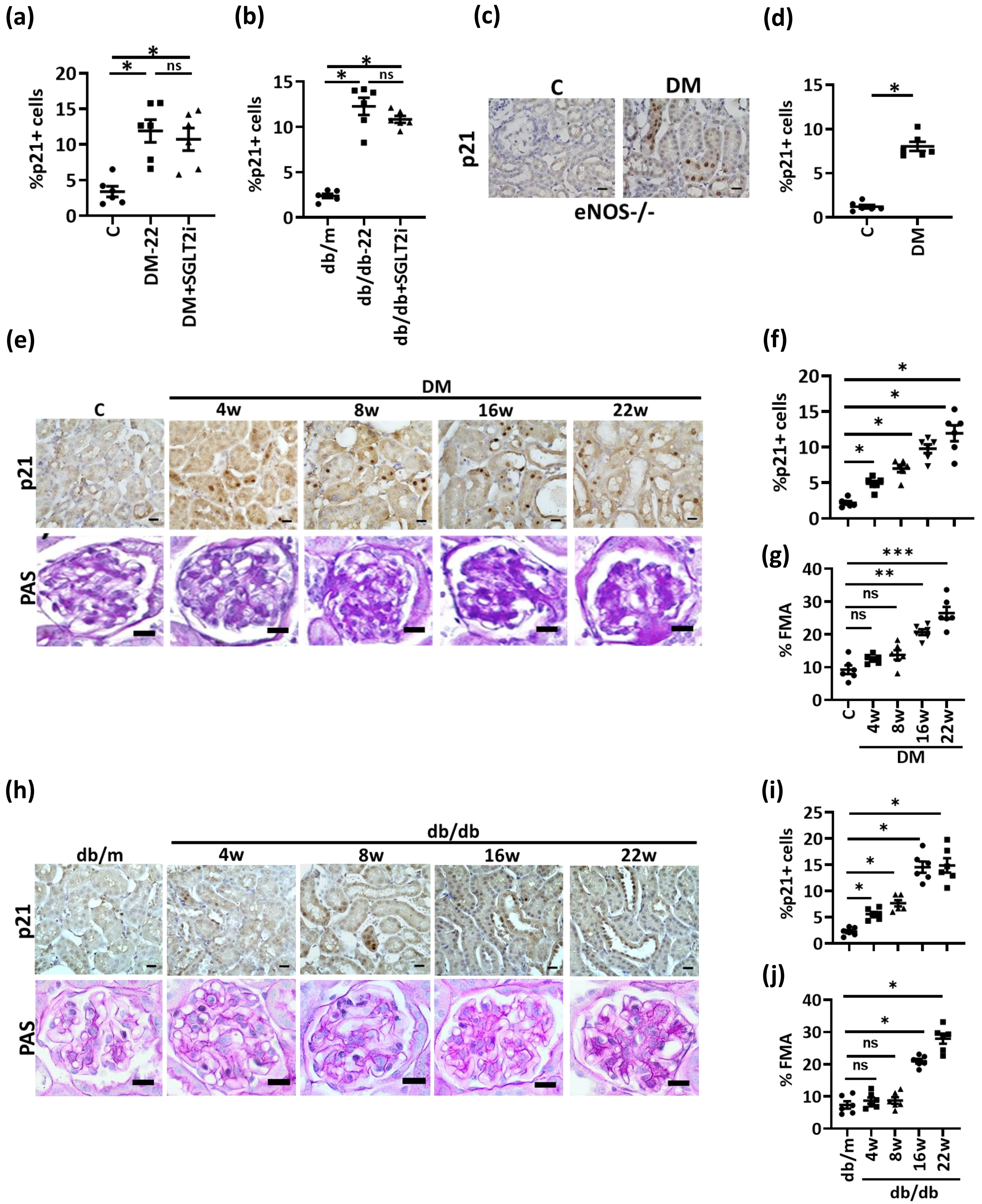

Supp. Fig. 3: Sustained p21 expression in various DKD models despite reduced blood glucose levels and in vivo time kinetics of p21 induction

a,b) Dot plots showing percentage of cells staining positive for p21 in vivo (T1DM, a; T2DM model, b) despite reducing glucose levels (DM+SGLT2i, a; db/db+SGLT2i, b) as compared to mice with persistently elevated glucose levels (DM-22, a; db/db, b) or normoglycemic controls (C, a; db/m, b). Dot plots reflecting mean±SEM of 6 mice per group; one-way ANOVA with Sidak's multiple comparison test, *P<0.05; ns: non-significant. P=0.001 (C vs DM-22), P=0.91 (DM-22 vs DM+SGLT2i), P=0.005 (C vs DM+SGLT2i), P<0.0001 (db/m vs db/db-22), P=0.31 (db/db-22 vs db/db+SGLT2i), P<0.0001 (db/m vs db/db+SGLT2i).

c,d) Exemplary histological images of p21 **(c)** in non-diabetic (C) or diabetic (T1DM) eNOS-/- mice (immunohistochemistry, p21 detected by HRP-DAB-reaction, brown; hematoxylin counterstain) and dot plot showing percentage of cells staining positive for p21 **(d)**. Dot plots reflecting mean±SEM of 6 mice per group; unpaired t-test, P<0.0001.

e-g) Exemplary histological images of p21 and of periodic acid Schiff staining (PAS; **e**) and dot plots summarizing results for p21 **(f)** and fractional mesangial area (**g**; FMA) in non-diabetic (C, control, age 28 weeks) or diabetic mice (DM; STZ-model; 4, 8, 16 and 22 weeks post STZ). p21 is detected by HRP-DAB reaction, brown; hematoxylin nuclear counter stain, blue. Scale bars represent 20 µm. Dot plots reflecting mean±SEM of 6 mice per group; one-way ANOVA with Sidak's multiple comparison test, *P<0.05; **P<0.01; ***P<0.001; ns: non-significant. **f)** P=0.04 (C vs 4w), P=0.0002 (C vs 8w), P<0.0001 (C vs 16w), P<0.0001 (C vs 22w). **g)** P=0.29 (C vs 4w), P=0.07 (C vs 8w), P<0.0001 (C vs 16w), P<0.0001 (C vs 22w).

h-j) Exemplary histological images of p21 and periodic acid Schiff staining (PAS; h) and dot plots summarizing results for p21 (i) and fractional mesangial area (j; FMA) in non-diabetic db/m (age 22 weeks) or diabetic db/db mice at 4, 8, 16 and 22 weeks of age. p21 is detected by HRP-DAB reaction, brown; hematoxylin nuclear counter stain, blue. Scale bars represent 20 µm. Dot plots reflecting mean±SEM of 6 mice per group; one-way ANOVA with Sidak's multiple comparison test, *P<0.05; ns: non-significant. **i)** P=0.04 (db/m vs 4w), P=0.0005 (db/m vs 8w), P<0.0001 (db/m vs 16w), P<0.0001 (db/m vs 22w). **j)** P=0.86 (db/m vs 4w), P=0.85 (db/m vs 8w), P<0.0001 (db/m vs 16w), P<0.0001 (db/m vs 22w).

Supp. Fig. 4 (related to Fig. 2)

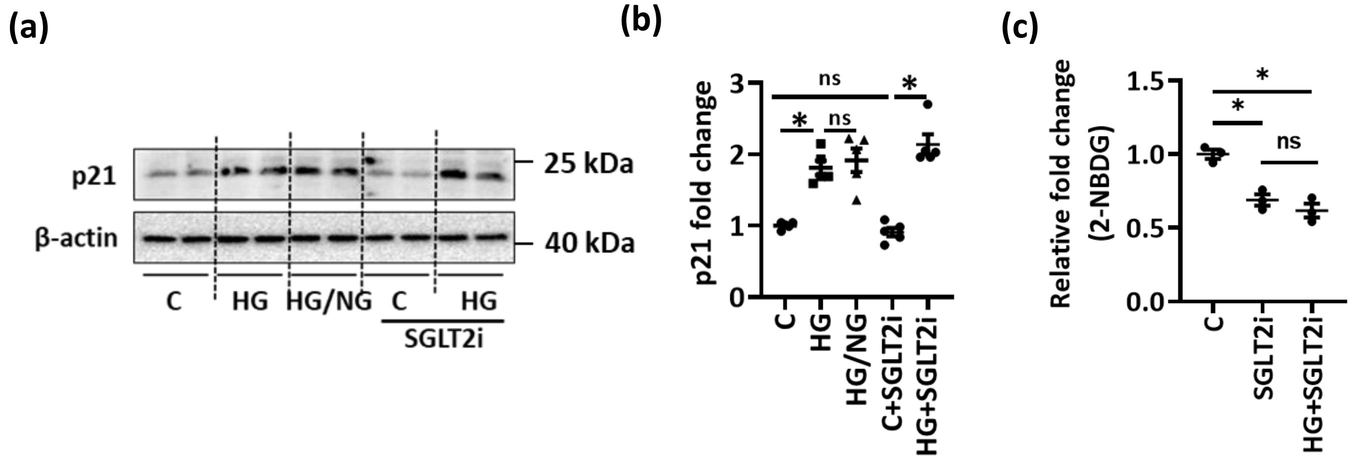

Supp. Fig. 4: Effect of SGLT2 inhibitor on p21 expression and glucose uptake in vitro

a,b) Exemplary immunoblot of p21 (a; β-actin: loading control) in BUMPT cells and dot plot summarizing results (b). Experimental conditions: control with continuously normal glucose (C, 5 mM glucose), continuously high glucose (HG, 25 mM, 48 h), high glucose for 24 h followed by normal glucose (NG, 5 mM glucose) for 24 h, control with SGLT2i (Dapagliflozin®, 2 µM, C+SGLT2i), or continuously high glucose with SGLT2i (Dapagliflozin®, 2 µM, added in the second 24h, HG+SGLT2i). Dot plot reflecting mean±SEM of 3 independent experiments; one-way ANOVA with Sidak's multiple comparison test, *P<0.05; ns: non-significant. P=0.0002 (C vs HG), P=0.94 (HG vs HG/NG), P=0.96 (C vs C+SGLT2i), P<0.0001 (C+SGLT2i vs HG+SGLT2i).

c) Dot plot reflecting glucose uptake by renal tubular cells (BUMPT) exposed to a fluorescent glucose tracer (2-NBDG, 200 µM) in the presence or absence of SGLT2i (Dapagliflozin®, 2 µM, 2 h). BUMPT cells were cultured in normal (5 mM glucose, C) or high (25 mM glucose, HG) glucose concentrations, and these concentrations were maintained during 2-NBDG exposure. Dot plot reflecting Mean mean± SEM of 3 independent repeat experiments; one-way ANOVA with Sidak's multiple comparison test, *P<0.05; ns: non-significant. P=0.0003 (C vs SGLT2i), P=0.45 (SGLT2i vs HG+SGLT2i), P=0.001 (C vs HG+SGLT2i).

Supp. Fig. 5 (related to Fig. 2)

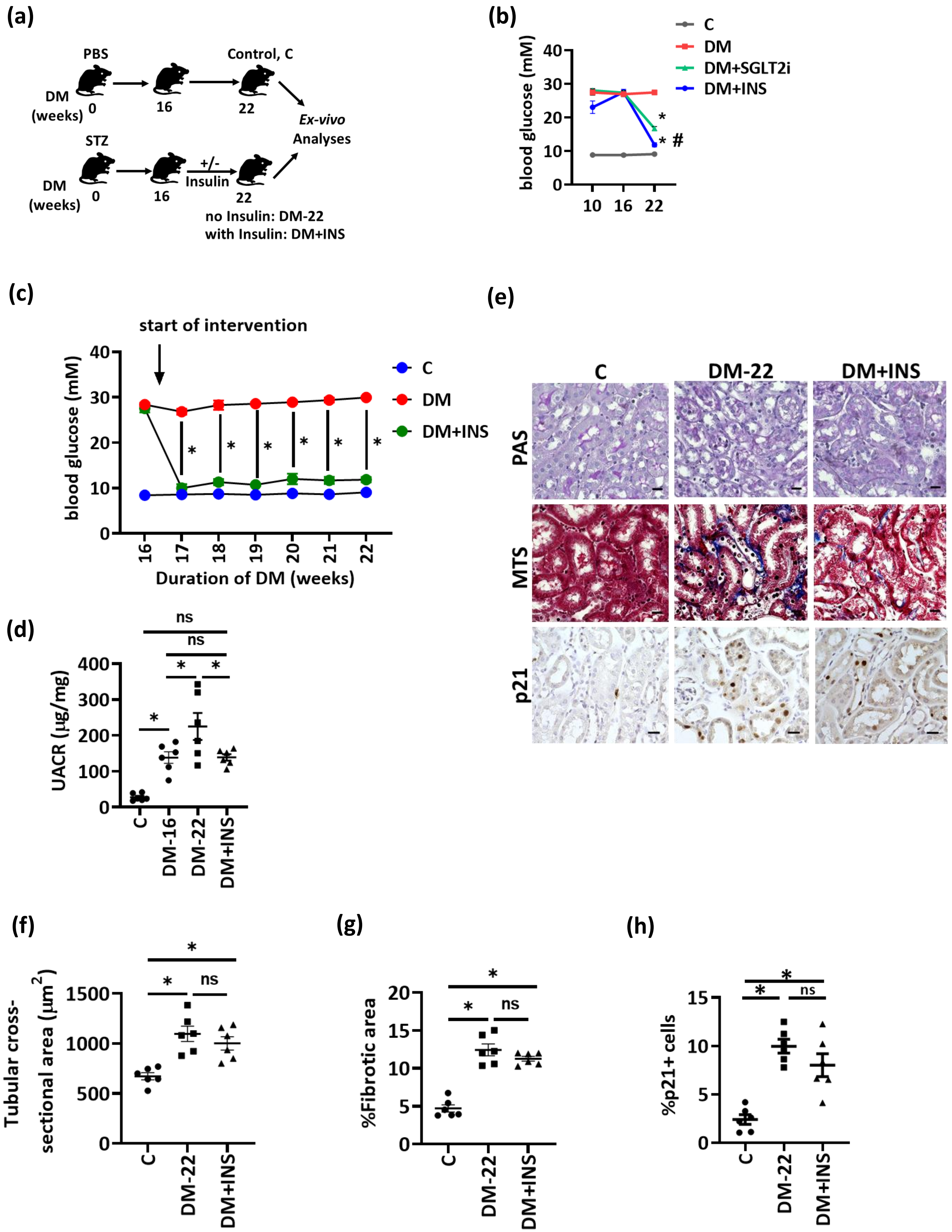

Supp. Fig. 5: Sustained p21 expression and impaired renal function despite reduced blood glucose levels with insulin

a) Experimental scheme showing non-diabetic control (C) or diabetic (STZ-model) mice without (DM) or with insulin treatment to reduce blood glucose levels (DM+INS). Mice were age matched and insulin treatment was started after 16 weeks of persistent hyperglycemia for further 6 weeks.

b) Average blood glucose levels in non-diabetic control (C) or diabetic mice without (DM) or with intervention to reduce blood glucose levels using SGLT2i (Dapagliflozin®; DM+SGLT2i) or insulin (DM+INS) after 10, 16, or 22 weeks of hyperglycemia. Interventions were started after 16 weeks of hyperglycemia. Line graphs reflecting mean±SEM of 6 mice per group; one-way ANOVA with Sidak's multiple comparison test, *$P<0.0001$ (compared to DM); # $P<0.001$ (compared to DM+SGLT2i). $P<0.0001$ (C vs DM), $P<0.0001$ (DM+INS vs DM), $P=0.0007$ (DM+INS vs DM+SGLT2i).

c) Line graph summarizing the weekly average blood glucose levels in the experimental groups (as described in a) from week 16 onwards. Intervention was started at 16 weeks of hyperglycemia. Line graphs reflecting mean±SEM of 6 mice per group; two-way ANOVA with Sidak's multiple comparison test, *$P<0.0001$.

d) Dot plot summarizing albuminuria (urinary albumin-creatinine ratio, µg albumin/mg creatinine; UACR) in experimental groups (as described in a). Dot plot reflecting mean±SEM of 6 mice per group; one-way ANOVA with Sidak's multiple comparison test, *$P<0.05$; ns: non-significant. $P=0.006$ (C vs DM-16), $P=0.006$ (C vs DM+INS), $P=0.04$ (DM-16 vs DM-22), $P>0.99$ (DM-16 vs DM+INS), $P=0.04$ (DM-22 vs DM+INS).

e-h) Exemplary histological images of periodic acid Schiff staining (PAS), interstitial fibrosis (Masson's trichrome stain, MTS), and p21 staining **(e)** and dot plots showing tubular cross-sectional area **(f)**, percentage fibrotic area **(g)** and percentage of cells staining positive for p21 **(h)** in experimental groups (as described in **a**). p21 is detected by HRP-DAB reaction, brown; hematoxylin nuclear counter stain, blue. Scale bars represent 20 µm. Dot plots reflecting mean±SEM of 6 mice per group; one-way ANOVA with Sidak's multiple comparison test, *$P<0.05$; ns: non-significant. **f)** $P=0.0006$ (C vs DM-22), $P=0.005$ (C vs DM+INS), $P=0.53$ (DM-22 vs DM+INS). **g)** $P<0.0001$ (C vs DM-22), $P<0.0001$ (C vs DM+INS), $P=0.32$ (DM-22 vs DM+INS). **h)** $P<0.0001$ (C vs DM-22), $P=0.0009$ (C vs DM+INS), $P=0.32$ (DM-22 vs DM+INS).

Supp. Fig. 6 (related to Fig. 2)

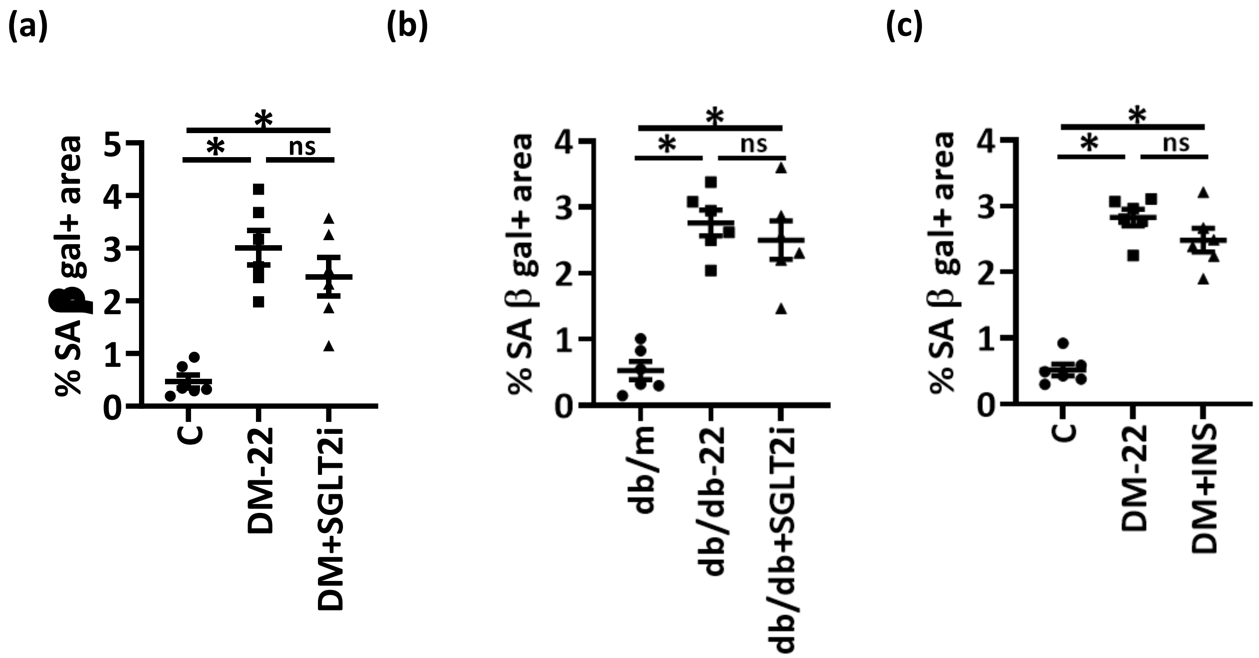

Supp. fig. 6: Sustained renal tubular senescence despite reduced blood glucose levels in experimental DKD

a-c) Dot plots showing percentage of SA-β-gal. positive area in vivo (STZ model, **a** and **c**; db/db model, **b**) despite reducing glucose levels (DM+SGLT2i, **a**; db/db+SGLT2i, **b**; or DM+INS, **c**) as compared to mice with persistently elevated glucose levels (DM-22, **a** and **c**; db/db-22, **b**) or normoglycemic controls (C, **a** and **c**; db/m, **b**). Dot plots reflecting mean±SEM of 6 mice per group; one-way ANOVA with Sidak's multiple comparison test, *P<0.05; ns: non-significant. **a)** P<0.0001 (C vs DM-22), P=0.0007 (C vs DM+SGLT2i), P=0.49 (DM-22 vs DM+SGLT2i). **b)** P<0.0001 (db/m vs db/db-22), P<0.0001(db/m vs db/db+SGLT2i), P=0.71 (db/db-22 vs db/db+SGLT2i). **c)** P<0.0001 (C vs DM-22), P<0.0001 (C vs DM+INS), P=0.26 (DM-22 vs DM+INS).

Supp. Fig. 7 (related to Fig. 2)

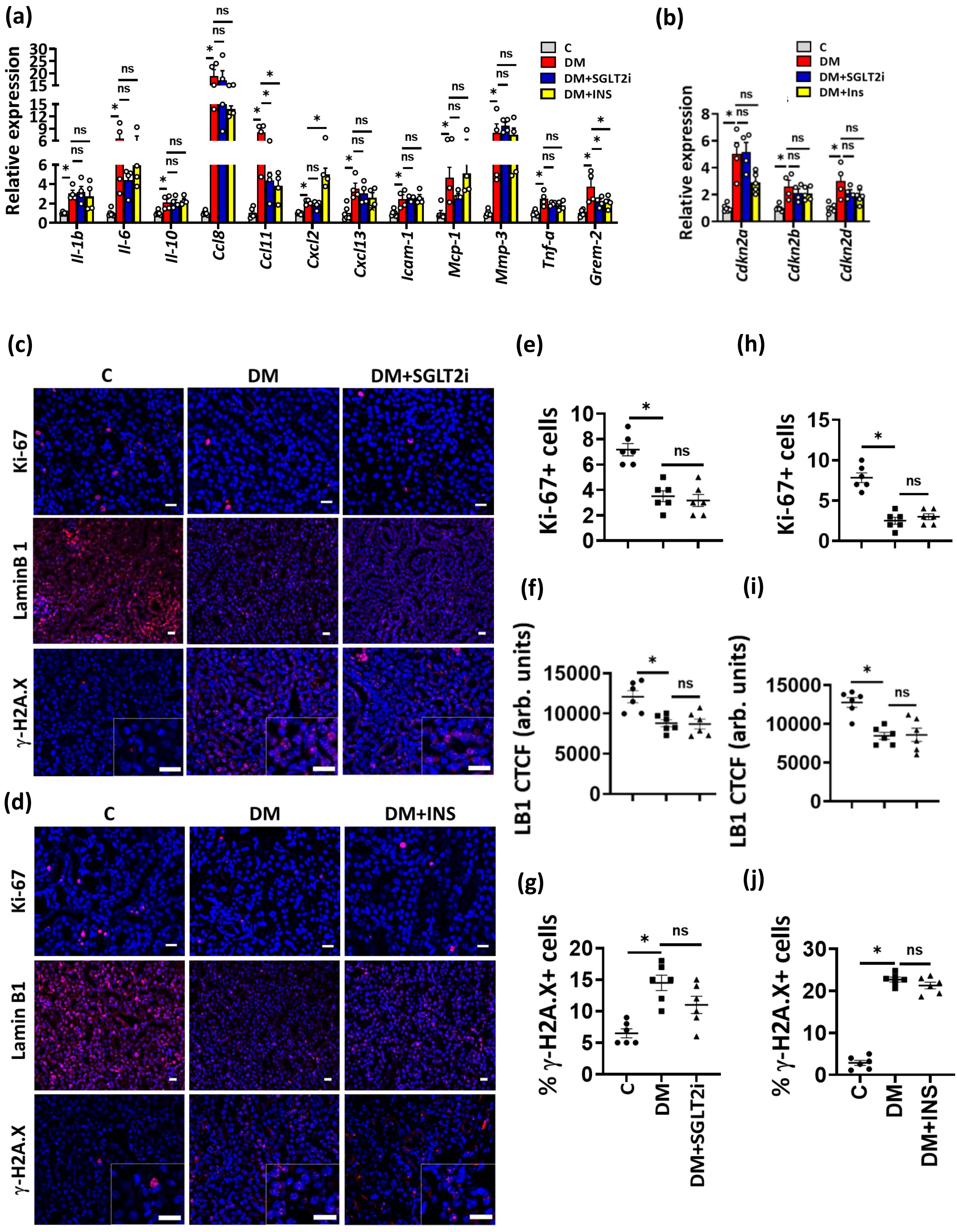

Supp. Fig. 7: Sustained renal tubular senescence in experimental DKD despite reduced blood glucose levels

a,b) Bar graphs summarizing expression of SASP genes **(a)** or cell cycle kinase inhibitors **(b)** in mouse kidneys (mRNA, qRT-PCR). Non-diabetic mice (C) were compared to diabetic mice (STZ model) without (DM) or with SGLT2i (DM+SGLT2i) or insulin (DM+INS) treatment to lower blood glucose levels. Bar graphs reflecting mean±SEM of 6 mice per group (C) or 4 mice per group (DM, DM+SGLT2i, DM+INS); kruskal wallis test with Dunn's multiple comparison test (IL-6, MCP-1, Mmp-3) or one-way ANOVA with Sidak's multiple comparison test (rest of the genes), *P<0.05; ns: non-significant. Specific p-values are as follows:

Il-1b) P=0.012 (C vs DM), P=0.99 (DM vs DM+SGLT2i), P=0.95 (DM vs DM+INS).
Il-6) P=0.017 (C vs DM), P>0.99 (DM vs DM+SGLT2i), P>0.99 (DM vs DM+INS).
Il-10) P=0.009 (C vs DM), P=0.99 (DM vs DM+SGLT2i), P=0.92 (DM vs DM+INS).
Ccl8) P<0.0001 (C vs DM), P=0.92 (DM vs DM+SGLT2i), P=0.33 (DM vs DM+INS).
Ccl11) P<0.0001 (C vs DM), P=0.01 (DM vs DM+SGLT2i), P=0.004 (DM vs DM+INS).
Cxcl2) P=0.04 (C vs DM), P=0.90 (DM vs DM+SGLT2i), P<0.0001 (DM vs DM+INS).
Cxcl13) P=0.0035 (C vs DM), P=0.87 (DM vs DM+SGLT2i), P=0.48 (DM vs DM+INS).
Icam-1) P=0.0003 (C vs DM), P=0.99 (DM vs DM+SGLT2i), P=0.98 (DM vs DM+INS).
Mcp-1) P=0.016 (C vs DM), P>0.99 (DM vs DM+SGLT2i), P>0.99 (DM vs DM+INS).
Mmp-3) P=0.003 (C vs DM), P=0.88 (DM vs DM+SGLT2i), P=0.81 (DM vs DM+INS).
Tnf-a) P=0.0004 (C vs DM), P=0.11 (DM vs DM+SGLT2i), P=0.09 (DM vs DM+INS).
Grem-2) P=0.0007 (C vs DM), P=0.04 (DM vs DM+SGLT2i), P=0.04 (DM vs DM+INS).
Cdkn2a) P=0.0001 (C vs DM), P=0.99 (DM vs DM+SGLT2i), P=0.03 (DM vs DM+INS).
Cdkn2b) P=0.001 (C vs DM), P=0.58 (DM vs DM+SGLT2i), P=0.45 (DM vs DM+INS).
Cdkn2d) P=0.001 (C vs DM), P=0.25 (DM vs DM+SGLT2i), P=0.059 (DM vs DM+INS).

c,d) Exemplary histological images of Ki-67, LaminB1, and γ-H2A.X staining in non-diabetic mice (C), diabetic mice (STZ model) without (DM) or with SGLT2i (DM+SGLT2i, c) or insulin (DM+INS, d) treatment for six weeks to lower blood glucose levels. Ki-67, LaminB1 (LB1) and γ-H2A.X are immunofluorescentlydetected, red; DAPI nuclear counterstain, blue; insets: larger magnification; all scale bars represent 20 µm.

(e-j): Dot plots showing number of cells staining positive for Ki-67 **(e, h)**, LaminB1 intensity (LB1; **f, i**) and percentage of cells staining positive for γ-H2A.X **(g, j)** in experimental groups (as described in **a**). Dot plots reflecting mean±SEMof 6 mice per group; one-way ANOVA with Sidak'smultiple comparison test, *P<0.05; ns: non-significant. CTCF: corrected total cell fluorescence. **e)** P<0.0001 (C vs DM), P=0.85 (DM vs DM+SGLT2i). **f)** P=0.0003 (C vs DM), P=0.99 (DM vs DM+SGLT2i). **g)** P=0.0003 (C vs DM-22), P=0.09 (DM vs DM+SGLT2i). **h)** P<0.0001 (C vs DM), P=0.71 (DM vs DM+INS). **i)** P=0.0008 (C vs DM), P=0.98 (DM vs DM+INS). j) P<0.0001 (C vs DM), P=0.28 (DM vs DM+INS).

Supp. Fig. 8 (related to Fig. 2)

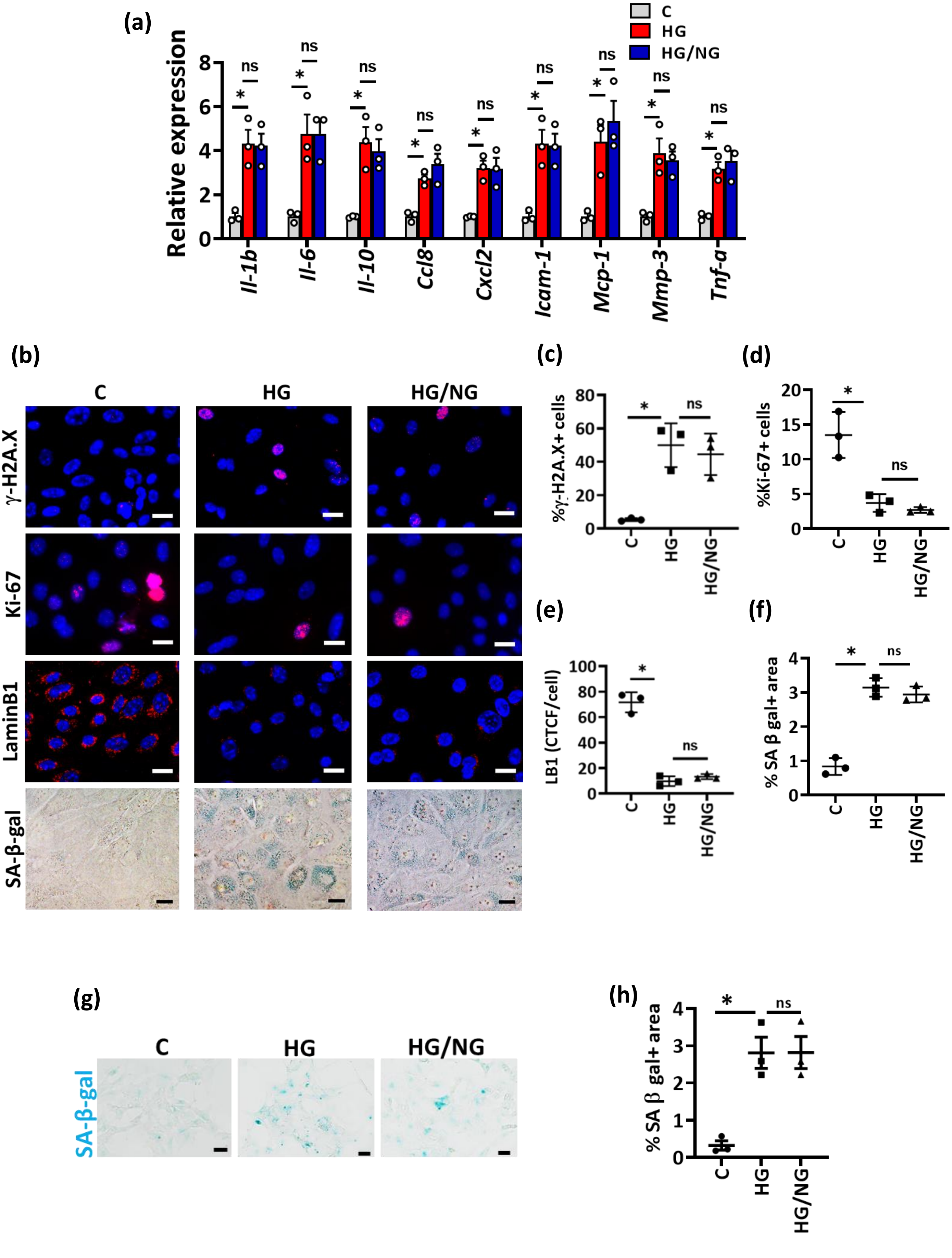

Supp. Fig. 8: Sustained senescence despite normalized glucose levels in murine primary tubular cells and HEK-293 cells

a)Bar graphs summarizing expression of SASP genes in mouse primary tubular cells (mRNA, qRT-PCR). Experimental conditions: control with continuously normal glucose (C, 5 mMglucose), continuously high glucose (HG, 25 mM, 48 h), and high glucose for 24 h followed by normal glucose (NG, 5 mM glucose) for 24 h. Bar graphs reflecting mean±SEM of 3 independent experiments; one-way ANOVA with Sidak's multiple comparison test, *P<0.05; ns: non-significant. Specific p-values are as follows:

Il-1b) P=0.001 (C vs HG), P=0.99 (HG vs HG/NG).

Il-6) P=0.004 (C vs HG), P>0.99 (HG vs HG/NG).

Il-10) P=0.003 (C vs HG), P=0.91 (HG vs HG/NG).

Ccl8) P=0.009 (C vs HG), P=0.40 (HG vs HG/NG).

Cxcl2) P=0.004 (C vs HG), P=0.99 (HG vs HG/NG).

Icam-1) P=0.001 (C vs HG), P=0.99 (HG vs HG/NG).

Mcp-1) P=0.011 (C vs HG), P=0.66 (HG vs HG/NG).

Mmp-3) P=0.002 (C vs HG), P=0.92 (HG vs HG/NG).

Tnf-a) P=0.002 (C vs HG), P=0.79 (HG vs HG/NG).

b-f) Exemplary images **(b)** and dot blot summarizing results **(c-f)** of γ-H2A.X, Ki-67, LaminB1 and SA-β-gal. (senescence associated β-galactosidase, blue) staining in experimental groups (as described in **a**). γ-H2A.X, Ki-67, and Lamin B1 (LB1) are immunofluorescently detected, red; DAPI nuclear counterstain, blue. Scale bars represent 20 µm. Dot plots reflecting mean±SEM of 3 independent experiments; one-way ANOVA with Sidak's multiple comparison test, *P<0.05; ns: non-significant. CTCF: corrected total cell fluorescence. **c)** P=0.001 (C vs HG), P=0.86 (HG vs HG/NG). **d)** P=0.0005 (C vs HG), P=0.88 (HG vs HG/NG). **e)** P=0.0006 (C vs HG), P=0.97 (HG vs HG/NG). **f)** P<0.0001 (C vs HG), P=0.77 (HG vs HG/NG).

g,h) Exemplary images of and SA-β-gal. (senescence associated β-galactosidase staining, blue, **g**) and dot plot showing percentage of SA-β-gal. positive area **(h)** in HEK-293 cells (experimental conditions as described in **a**). Dot plot reflecting mean±SEM of 3 independent experiments; one-way ANOVA with Sidak's multiple comparison test, *P<0.05; ns: non-significant. P=0.005 (C vs HG), P=0.99 (HG vs HG/NG).

Supp. Fig. 9 (related to Fig. 3)

(a)

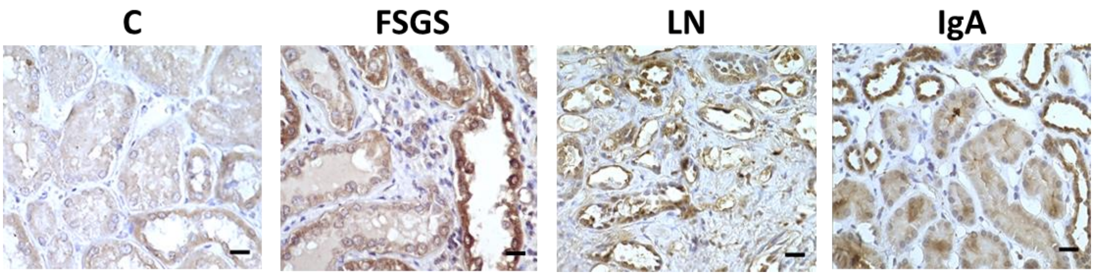

(b)

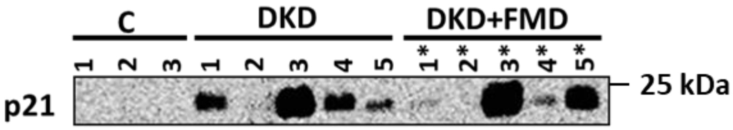

(c)

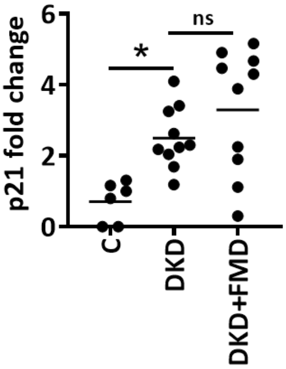

(d)

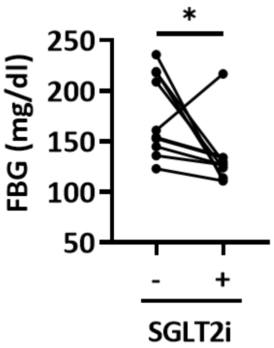

Supp. Fig. 9: Sustained p21 in patients with DKD and with other CKD

a) Exemplary histological images of human kidney sections (n=2 per group) stained for p21 obtained from controls (C), patients with Focal segmental glomerulosclerosis (FSGS), IgA nephropathy (IgA) and patients with Lupus nephritis (LN); p21 is detected by HRP-DAB reaction, brown; hematoxylin nuclear counter stain, blue. Scale bar represent 20 µm.

b, c) Exemplary immunoblot **(b)** and dot plot **(c)** summarizing results of urinary p21 in diabetic patients with known DKD (microalbuminuria) before (DKD; n=10) and after fasting mimicking diet (DKD+FMD; n=10) compared to controls (C; n=6). Dot plot reflecting mean±SEM; one-way ANOVA with Sidak's multiple comparison test, *P<0.05; ns: non-significant. P=0.02 (C vs DKD), P=0.308 (DKD vs DKD+FMD).

d) Line-graph illustrating individual changes of fasting blood glucose (FBG; mg/dl) in DKD patients (n=10) before and after SGLT2i treatment. Paired t-test, *P=0.03.

Supp. Fig. 10 (related to Fig. 4)

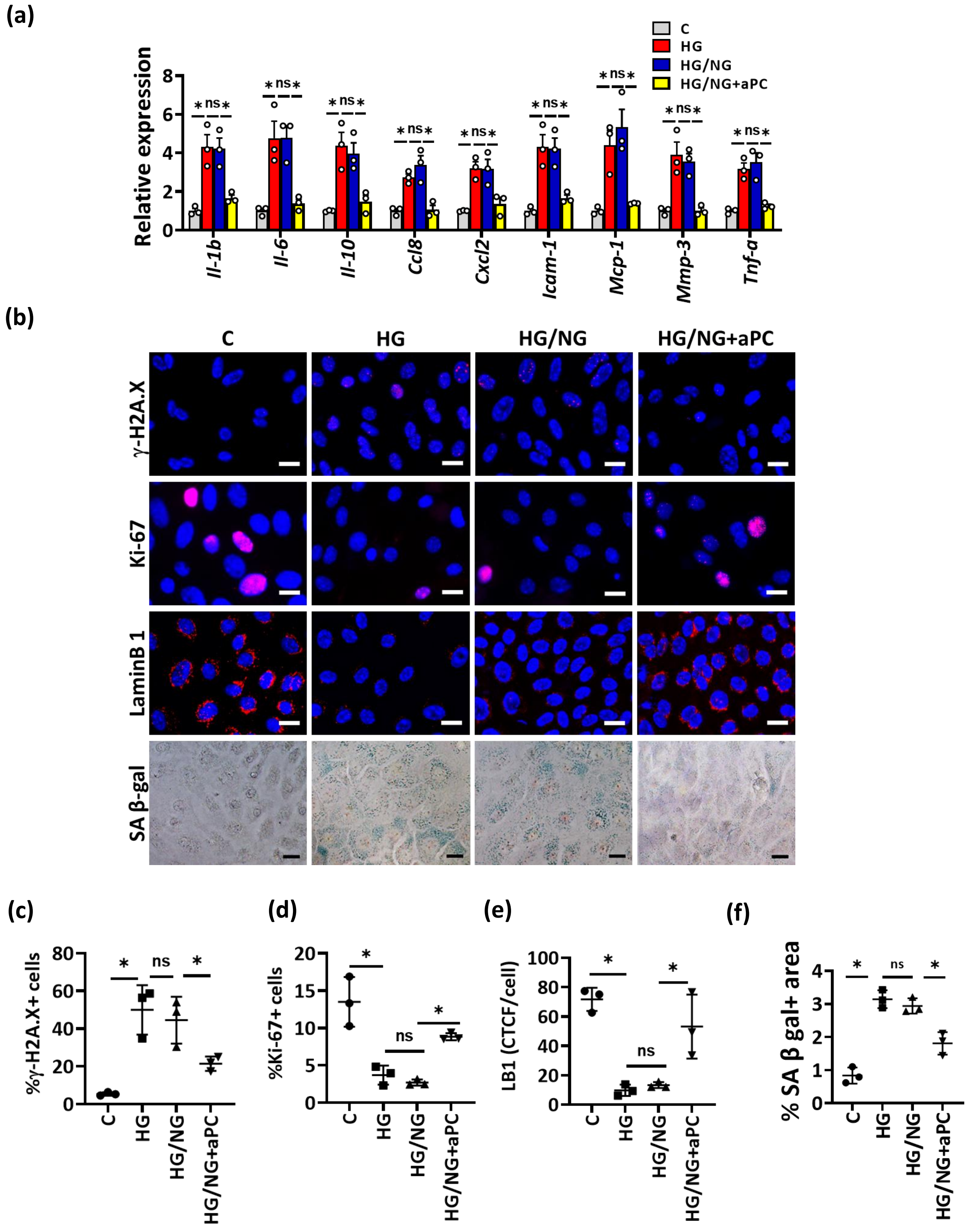

Supp. Fig. 10: aPC reverses epigenetically sustained renal tubular senescence *in vitro*.

a) Bar graphs summarizing expression of SASP genes in mouse primary tubular cells (mRNA, qRT-PCR). Experimental conditions (performed together with those shown in suppfig. 8): control with continuously normal glucose (C, 5 mM glucose), continuously high glucose (HG, 25 mM, 48 h), high glucose for 24 h followed by normal glucose (NG, 5 mM glucose) for 24 h without aPC-exposure (HG/NG), or with aPC(20 nM) upon returning cells to normal glucose (HG/NG+aPC). Bar graphs reflecting mean±SEM of 3 independent experiments; one-way ANOVA with Sidak's multiple comparison test, *P<0.05; ns: non-significant. Specific p-values are as follows:

Il-1b) P=0.001 (C vs HG), P=0.99 (HG vs HG/NG), P=0.009 (HG/NG vs HG/NG+aPC).

Il-6) P=0.004 (C vs HG), P>0.99 (HG vs HG/NG),P=0.008 (HG/NG vs HG/NG+aPC).

Il-10) P=0.003 (C vs HG), P=0.91 (HG vs HG/NG),P=0.018 (HG/NG vs HG/NG+aPC).

Ccl8) P=0.009 (C vs HG), P=0.40 (HG vs HG/NG),P=0.001 (HG/NG vs HG/NG+aPC).

Cxcl2) P=0.004 (C vs HG), P=0.99 (HG vs HG/NG),P=0.014 (HG/NG vs HG/NG+aPC).

Icam-1) P=0.001 (C vs HG), P=0.99 (HG vs HG/NG), P=0.001 (HG/NG vs HG/NG+aPC).

Mcp-1) P=0.011 (C vs HG), P=0.66 (HG vs HG/NG), P=0.004 (HG/NG vs HG/NG+aPC).

Mmp-3) P=0.002 (C vs HG), P=0.92 (HG vs HG/NG), P=0.006 (HG/NG vs HG/NG+aPC).

Tnf-a) P=0.002 (C vs HG), P=0.79 (HG vs HG/NG), P=0.001 (HG/NG vs HG/NG+aPC).

b) Exemplary images of γ-H2A.X , Ki-67, LaminB1 and SA-β-gal. (senescence associated β-galactosidase, blue) staining in experimental groups (as described in a). γ-H2A.X, Ki-67, and Lamin B1 are immunofluorescently detected, red; DAPI nuclear counterstain, blue. Scale bars represent 20 µm.

c-f) Dot plots showing percentage of cells staining positive for γ-H2A.X **(c)**, percentage of cells staining positive for Ki-67 **(d)**, LaminB1 intensity (LB1; **e**) and percentage of SA-β-gal. positive area **(f)** in experimental groups (as described in a).Dot plots reflecting mean±SEM of 3 independent experiments; one-way ANOVA with Sidak's multiple comparison test, *P<0.05; ns: non-significant. CTCF: corrected total cell fluorescence. **c)** P=0.001 (C vs HG), P=0.86 (HG vs HG/NG), P=0.004 (HG/NG vs HG/NG+aPC). **d)** P=0.0005 (C vs HG), P=0.88 (HG vs HG/NG), P=0.0009 (HG/NG vs HG/NG+aPC). **e)** P=0.0006 (C vs HG), P=0.97 (HG vs HG/NG), P=0.0009 (HG/NG vs HG/NG+aPC). **f)** P<0.0001 (C vs HG), P=0.77 (HG vs HG/NG), P=0.003 (HG/NG vs HG/NG+aPC).

Supp. Fig. 11 (related to Fig. 4)

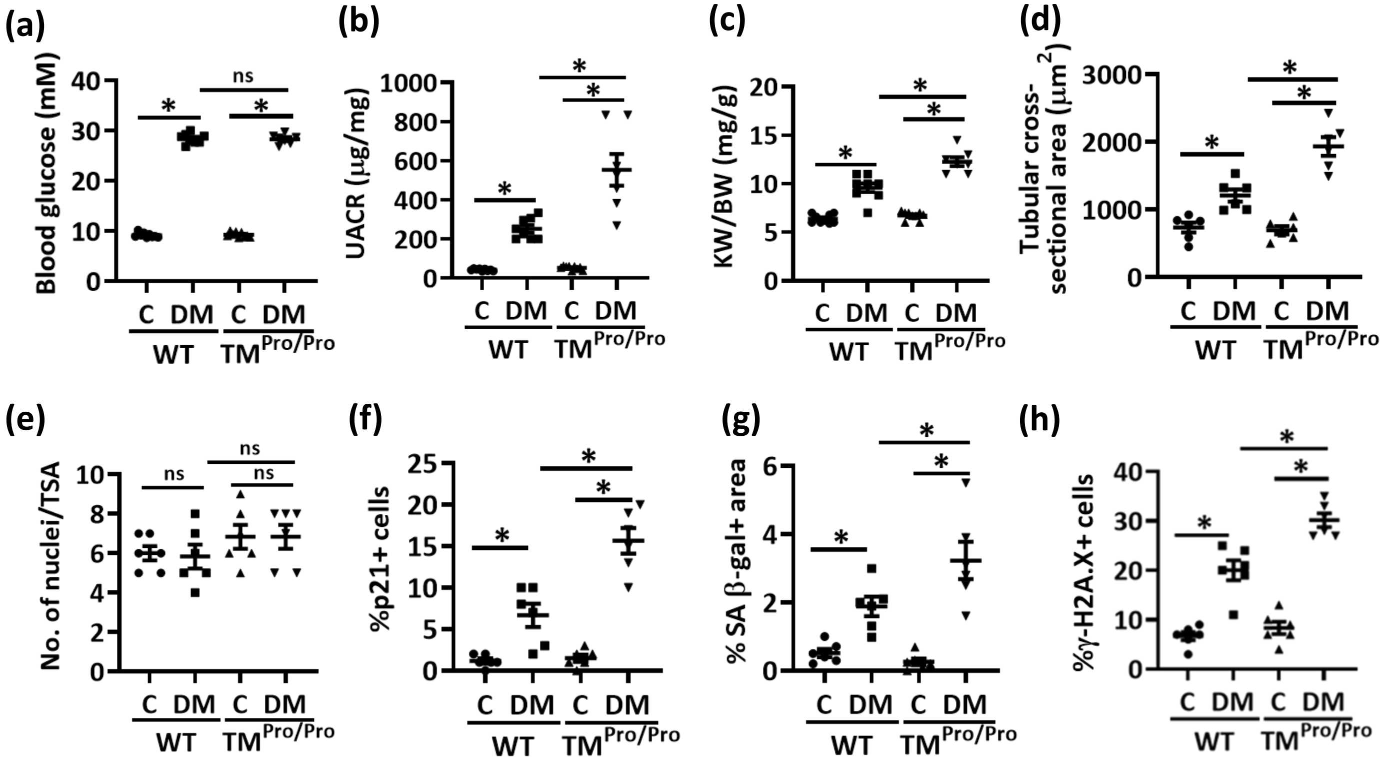

Supp. Fig. 11: Loss of thrombomodulin-dependent protein C activation enhances p21 expression and tubular senescence

a-c) Dot plots summarizing average blood glucose levels **(a)**, albuminuria (urinary albumin-creatinine ratio, µg albumin/mg creatinine; UACR, **b)** and adjusted kidney weight (mg kidney weight /g body weight; KW/BW, **c)** in non-diabetic (C) or diabetic (DM) wild-type (Wt) and $TM^{Pro/Pro}$ mice. Dot plots reflecting mean±SEM of 9 (WT-C), 8 (WT-DM), 9 ($TM^{Pro/Pro}$-C) and 7 ($TM^{Pro/Pro}$-DM)mice per group; two-way ANOVA with Sidak's multiple comparison test, *P<0.05; ns: non-significant. **a)** P<0.0001(WT-C vs WT-DM), P<0.0001($TM^{Pro/Pro}$-C vs $TM^{Pro/Pro}$-DM), P>0.99 (WT-DM vs $TM^{Pro/Pro}$-DM). **b)** P=0.001 (WT-C vs WT-DM), P<0.0001 ($TM^{Pro/Pro}$-C vs $TM^{Pro/Pro}$-DM), P<0.0001 (WT-DM vs $TM^{Pro/Pro}$-DM). **c)** P<0.0001 (WT-C vs WT-DM), P<0.0001 ($TM^{Pro/Pro}$-C vs $TM^{Pro/Pro}$-DM), P<0.0001 (WT-DM vs $TM^{Pro/Pro}$-DM).

d-h) Dot plots summarizing tubular hypertrophy (tubular cross-sectional area, TSA, d; and number of nuclei per TSA, e) and tubular cell senescence (p21 positive cells, f; SA-β-gal positive area, g; and γ-H2A.X positive cells, h) in experimental groups (as described in a). The number of nuclei per tubular cross-sectional area (TSA) is not different among experimental groups (e). Dot plots reflecting mean±SEM of 6 mice per group. two-way ANOVA with Sidak's multiple comparison test; *P<0.05; ns: non-significant.

d) P=0.01 (WT-C vs WT-DM), P<0.0001 ($TM^{Pro/Pro}$-C vs $TM^{Pro/Pro}$-DM), P=0.0002 (WT-DM vs $TM^{Pro/Pro}$-DM).
e) P>0.99 (WT-C vs WT-DM), >0.99 ($TM^{Pro/Pro}$-C vs $TM^{Pro/Pro}$-DM), P=0.76 (WT-DM vs $TM^{Pro/Pro}$-DM).
f) P=0.008 (WT-C vs WT-DM), P<0.0001 (TMPro/Pro-C vs $TM^{Pro/Pro}$-DM), P<0.0001 (WT-DM vs $TM^{Pro/Pro}$-DM). **g)** P=0.03 (WT-C vs WT-DM), P<0.0001 ($TM^{Pro/Pro}$-C vs $TM^{Pro/Pro}$-DM), P=0.03 (WT-DM vs $TM^{Pro/Pro}$-DM). **h)** P<0.0001 (WT-C vs WT-DM), P<0.0001 ($TM^{Pro/Pro}$ -C vs $TM^{Pro/Pro}$ -DM), P=0.0004 (WT-DM vs $TM^{Pro/Pro}$-DM).

Supp. Fig. 12 (related to Fig. 4)

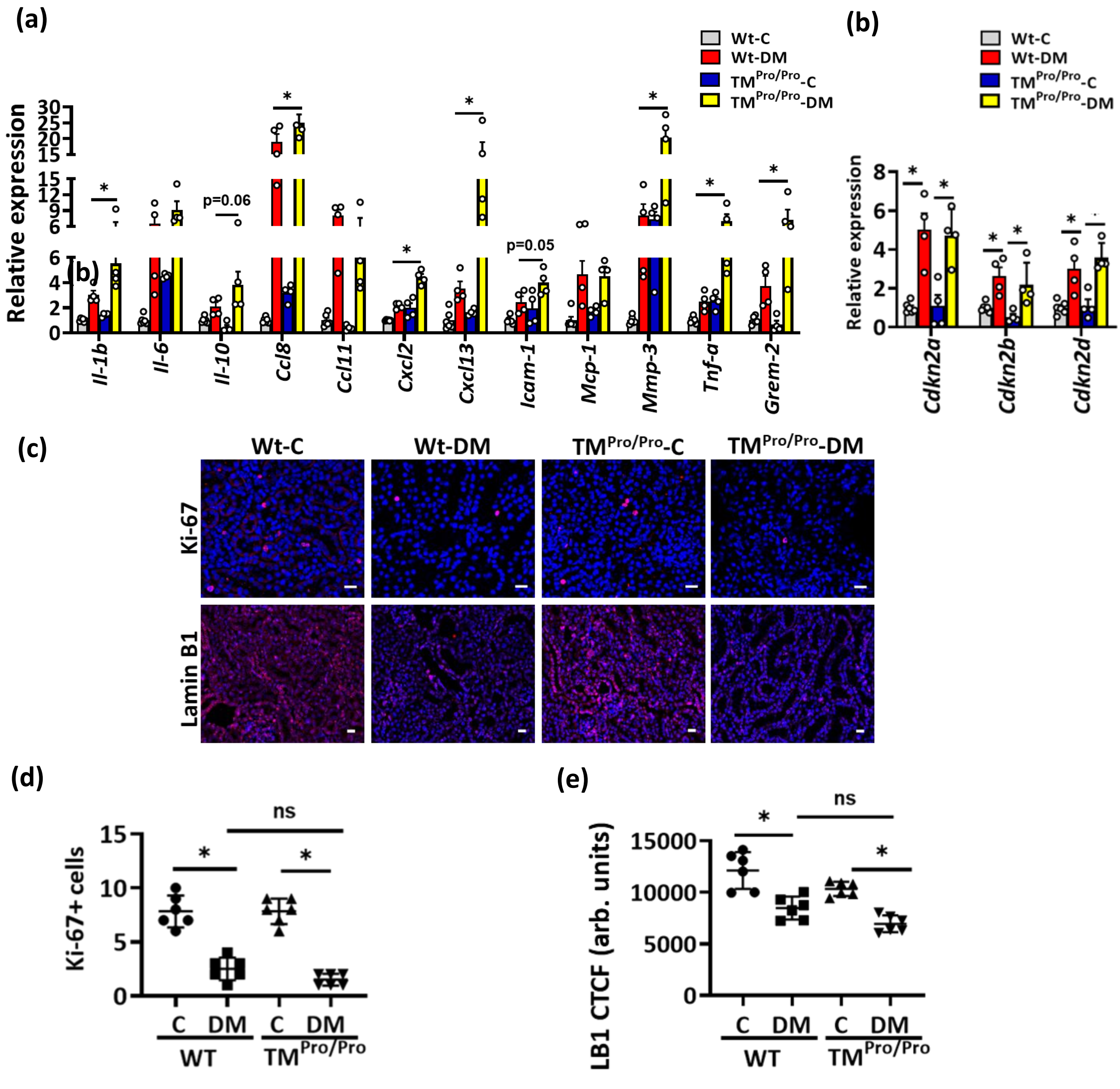

Supp. Fig. 12: Loss of thrombomodulin-dependent protein C activation enhances tubular senescence

a, b) Bar graphs summarizing expression of SASP genes **(a)** or cell cycle kinase inhibitors **(b)** in mouse kidneys (mRNA, qRT-PCR). Non-diabetic wild-type mice (Wt-C; n=6) or $TM^{Pro/Pro}$ mice ($TM^{Pro/Pro}$ -C; n=4) were compared to diabetic wild-type mice (STZ model; Wt-DM; n=4) or diabetic $TM^{Pro/Pro}$ mice (STZ model; $TM^{Pro/Pro}$ -DM; n=4). Bar graphs reflecting mean±SEM; two-way ANOVA comparing only column effect **(a)** or comparing only row effect **(b)** with Sidak's multiple comparison test, *P<0.05; ns: non-significant. Specific p-values are as follows:

Il-1b) P=0.02.

Il-6) P=0.22.

Il-10) P=0.06.

Ccl8) P=0.004.

Ccl11) P=0.25.

Cxcl2) P<0.0001.

Cxcl13) P=0.001.

Icam-1) P=0.05.

Mcp-1) P=0.98.

Mmp-3) P=0.001.

Tnf-a) P=0.003.

Grem-2) P=0.04.

Cdkn2a) P=0.002 (WT-C vs WT-DM), P=0.001 ($TM^{Pro/Pro}$-C vs $TM^{Pro/Pro}$-DM).

Cdkn2b) P=0.006 (WT-C vs WT-DM), P=0.01 ($TM^{Pro/Pro}$-C vs $TM^{Pro/Pro}$-DM).

Cdkn2d) P=0.002 (WT-C vs WT-DM), P=0.0008 ($TM^{Pro/Pro}$-C vs $TM^{Pro/Pro}$-DM).

c-e) Exemplary histological images **(c)** and dot blots summarizing results **(d,e)** of Ki-67 and LaminB1 staining in experimental groups (as describes in **a**). Ki-67 and Lamin B1 (LB1) are immunofluorescently detected, red; DAPI nuclear counterstain, blue. Scale bars represent 20 µm. Dot plots reflecting mean±SEM of 6 mice per group; two-way ANOVA with Sidak's multiple comparison test, *P<0.05; ns: non-significant. CTCF: corrected total cell fluorescence. **d)** P<0.0001 (WT-C vs WT-DM), P<0.0001 ($TM^{Pro/Pro}$-C vs $TM^{Pro/Pro}$-DM), P=0.25 (WT-DM vs $TM^{Pro/Pro}$ -DM). **e)** P<0.0001 (WT-C vs WT-DM), P=0.0001 ($TM^{Pro/Pro}$ -C vs $TM^{Pro/Pro}$ -DM), P=0.07 (WT-DM vs $TM^{Pro/Pro}$ -DM).

Supp. Fig. 13 (related to Fig. 5)

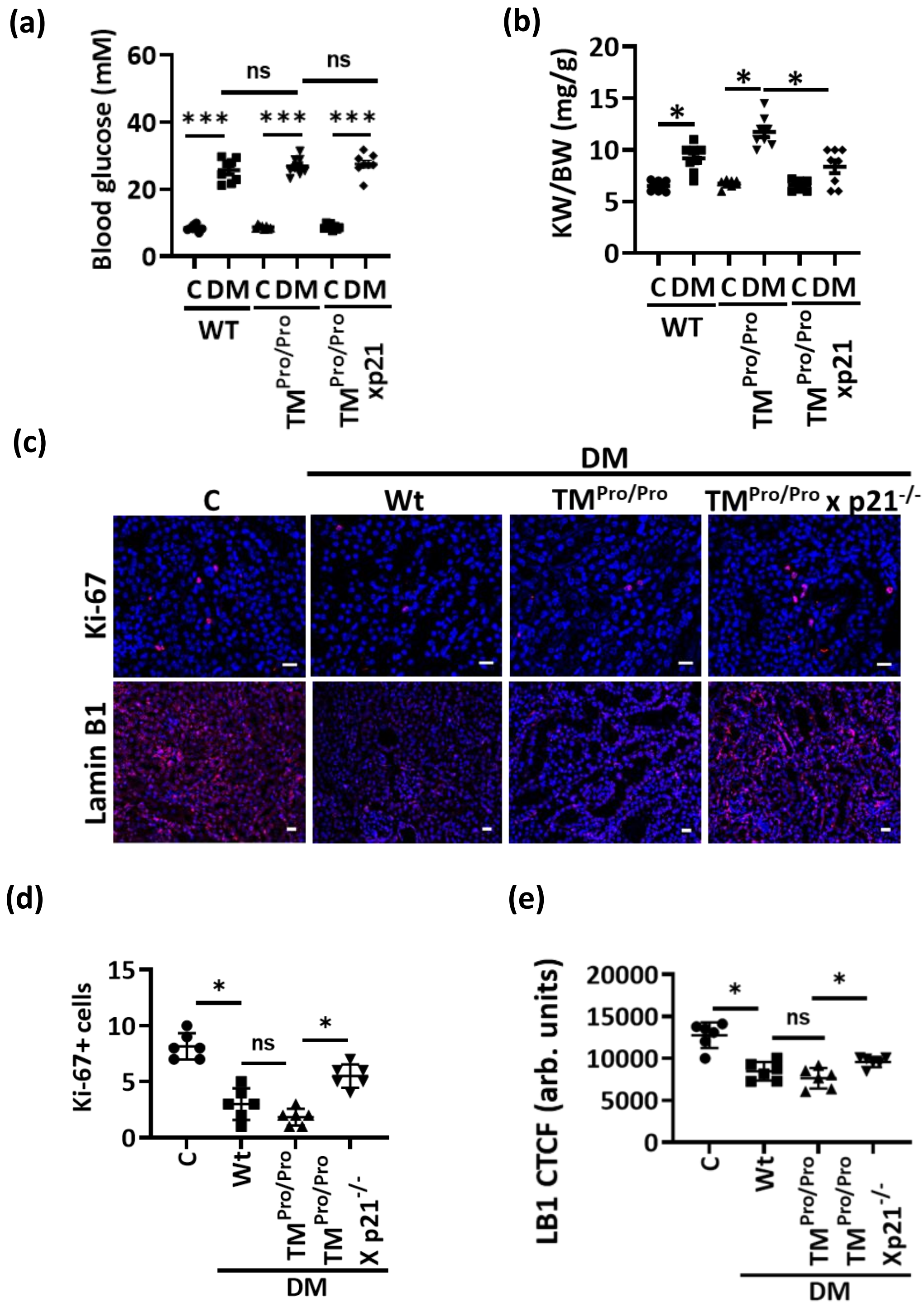

Supp. Fig. 13: p21 promotes renal tubular senescence in aPC-deficient mice

a,b) Dot plots summarizing average blood glucose levels **(a)** and adjusted kidney weight (mg kidney weight /g body weight; KW/BW, **b)** in non-diabetic (C) or diabetic (DM) wild-type (Wt), $TM^{Pro/Pro}$, or $TM^{Pro/Pro}$ x $p21^{-/-}$mice. Dot plots reflecting mean±SEM of 8 mice per group; two-way ANOVA with Sidak's multiple comparison test, *P<0.05; ***P<0.001; ns: non-significant. **a)** P<0.0001 (WT-C vs WT-DM), P<0.0001 ($TM^{Pro/Pro}$-C vs $TM^{Pro/Pro}$-DM), P<0.0001($TM^{Pro/Pro}$ x $p21^{-/-}$-Cvs $TMP^{ro/Pro}$ x $p21^{-/-}$-DM), P=0.8 (WT-DM vs $TM^{Pro/Pro}$-DM), P=>0.99 ($TM^{Pro/Pro}$-DM vs $TM^{Pro/Pro}$ x $p21^{-/-}$-DM). **b)** P=0.0003 (WT-C vs WT-DM), P<0.0001 ($TM^{Pro/Pro}$-C vs $TM^{Pro/Pro}$-DM), P<0.0001 ($TM^{Pro/Pro}$-DM vs $TM^{Pro/Pro}$ x $p21^{-/-}$-DM).

c-e) Exemplary histological images **(c)** and dot plots summarizing results **(d,e)** of Ki-67 and LaminB1 staining in experimental groups (as described in **a**). Ki-67 and LaminB1 (LB1) are immunofluorescently detected, red; DAPI nuclear counterstain, blue. Scale bars represent 20 µm. Dot plots reflecting mean±SEM of 6 mice per group; one-way ANOVA with Sidak's multiple comparison test, *P<0.05; ns: non-significant. CTCF: corrected total cell fluorescence. **d)** P<0.0001 (WT-C vs WT-DM), P=0.23 (WT-DM vs $TM^{Pro/Pro}$-DM), P<0.0001 ($TM^{Pro/Pro}$-DM vs $TM^{Pro/Pro}$ x $p21^{-/-}$-DM). **e)** P<0.0001 (WT-C vs WT-DM), P=0.55 (WT-DM vs $TM^{Pro/Pro}$-DM), P=0.02 ($TM^{Pro/Pro}$-DM vs $TM^{Pro/Pro}$ x $p21^{-/-}$-DM).

Supp. Fig. 14 (related to Fig. 5)

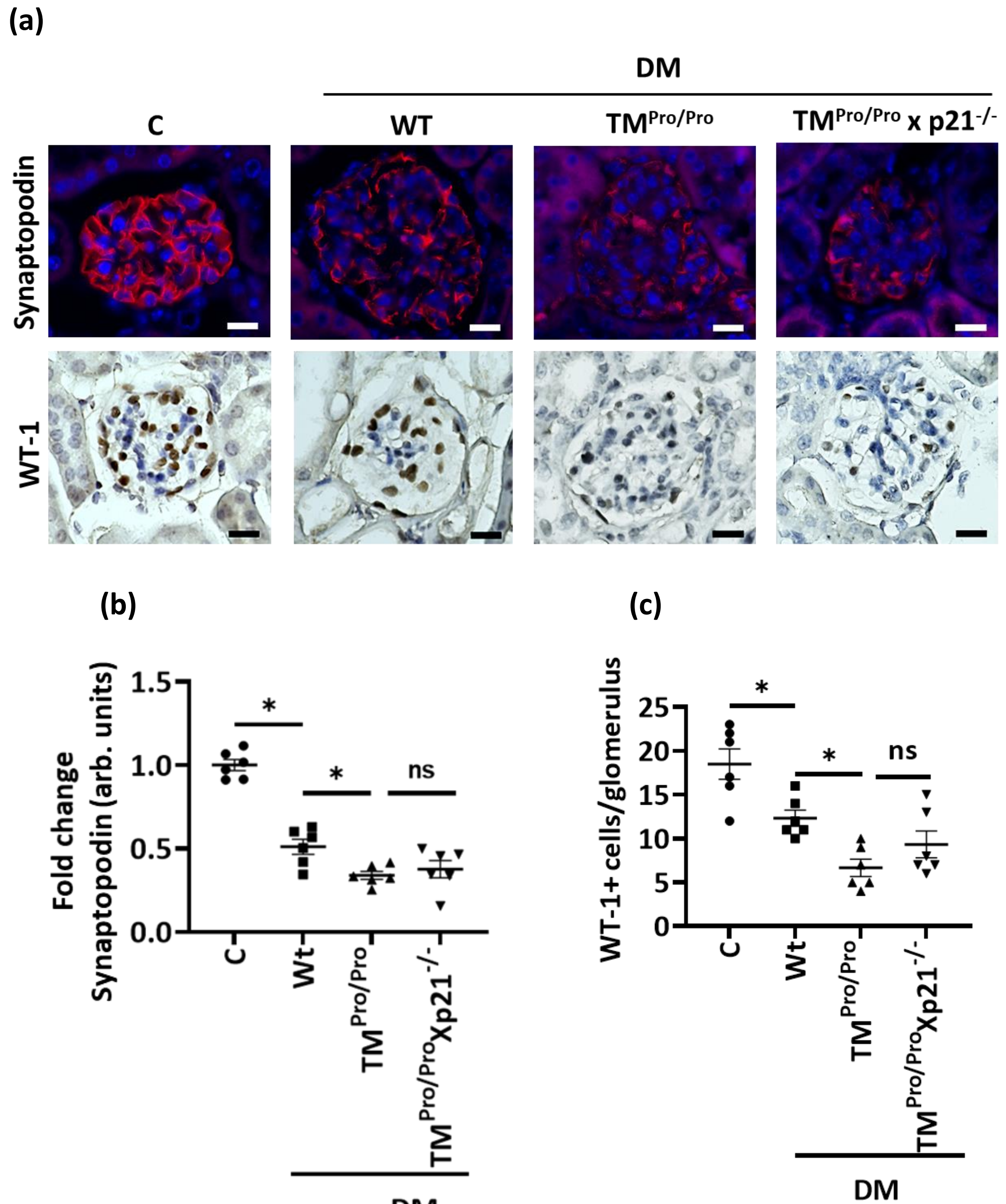

Supp. Fig. 14: p21 loss does not rescue glomerular damage in diabetic aPC-deficient mice

a) Exemplary histological images (a) and dot plots summarizing results (b,c) of synaptopodin and Wilms' tumor 1 (WT-1) staining in in non-diabetic (C) or diabetic (DM) wild-type (Wt), $TM^{Pro/Pro}$, or $TM^{Pro/Pro}$ x $p21^{-/-}$ mice. Synaptopodin is immunofluorescently detected, red; DAPI nuclear counterstain, blue. WT-1 is detected by HRP-DAB reaction, brown; hematoxylin nuclear counter stain, blue. Scale bar represent 20 µm. Dot plots reflecting mean±SEM of 6 mice per group; one-way ANOVA with Sidak's multiple comparison test, **P*<0.05; ns: non-significant. **b)** P<0.0001 (WT-C vs WT-DM), P=0.01 (WT-DM vs $TM^{Pro/Pro}$-DM), P=0.88 ($TM^{Pro/Pro}$-DM vs $TM^{Pro/Pro}$ x $p21^{-/-}$-DM). **c)** P=0.01 (WT-C vs WT-DM), P=0.02 (WT-DM vs $TM^{Pro/Pro}$-DM), P=0.43 ($TM^{Pro/Pro}$-DM vs $TM^{Pro/Pro}$ x $p21^{-/-}$-DM).

Supp. Fig. 15 (related to Fig. 6)

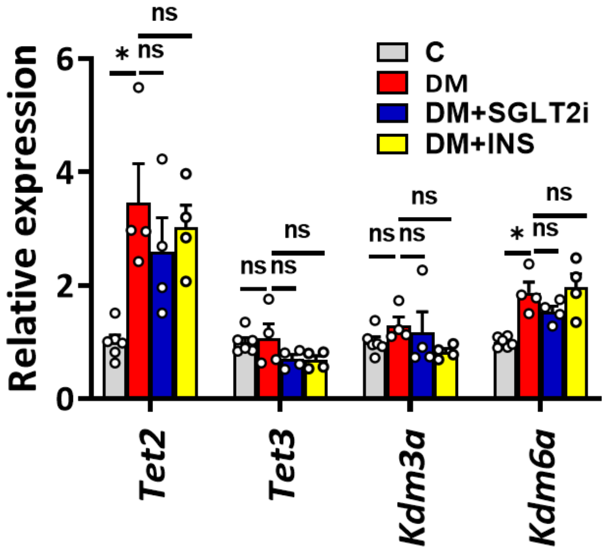

Supp. Fig. 15: Expression of demethylation regulators upon glucose lowering

Bar graph summarizing gene expression of demethylases (*Tet2, Tet3, Kdm3a* and *Kdm6a*) in mouse kidneys (mRNA, qRT-PCR). Non-diabetic mice (C; n=6) were compared to diabetic mice (STZ model; DM; n=4) or diabetic mice treated for six weeks with SGLT2i (DM+SGLT2i; n=4) or insulin (DM+INS; n=4). Bar graphs reflecting mean±SEM; one-way ANOVA with Sidak's multiple comparison test, *P<0.05; ns: non-significant.

***Tet2*)** P=0.003 (C vs DM), P=0.51 (DM vs DM+SGLT2i), P=0.88 (DM vs DM+INS).

***Tet3*)** P=0.98 (C vs DM), P=0.26 (DM vs DM+SGLT2i), P=0.22 (DM vs DM+INS).

***Kdm3a*)** P=0.60 (C vs DM), P=0.95 (DM vs DM+SGLT2i), P=0.30 (DM vs DM+INS).

***Kdm6a*)** P=0.001 (C vs DM), P=0.34 (DM vs DM+SGLT2i), P=0.96 (DM vs DM+INS).

Supp. Fig. 16 (related to Fig. 6)

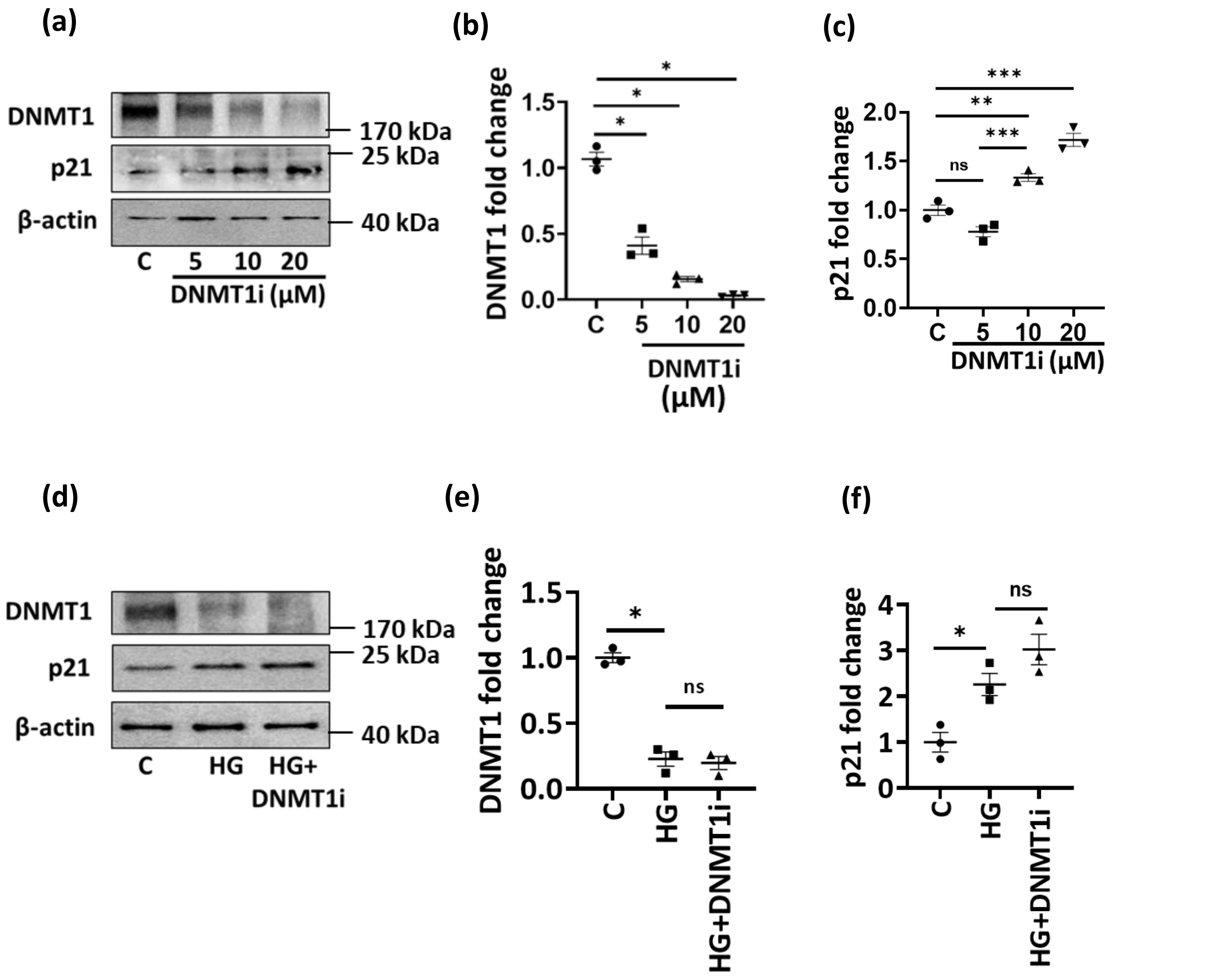

Supp. Fig. 16: Regulation of p21 expression by DNMT1 *in vitro*

a-c) Exemplary immunoblots of DNMT1 and p21 (a; β-actin: loading control) in BUMPT cells and dot plots summarizing results (c,d). Experimental conditions: control (C), DNMT1 vivo morpholino (5, 10 or 20 µM, 24h, DNMT1i). Dot plots reflecting mean±SEM of 3 independent experiments; one-way ANOVA with Sidak's multiple comparison test, **P*<0.05; ns: non-significant. **b)** P<0.0001 (C vs 5), P<0.0001 (C vs 10), P<0.0001 (C vs 20). **c)** P=0.07 (C vs 5), P=0.009 (C vs 10), P<0.0001 (C vs 20), P=0.0003 (5 vs 10).

d-f) Exemplary immunoblots of DNMT1 and p21 (d; β-actin: loading control) in BUMPT cells and dot plots summarizing results (e, f). Experimental conditions: control with normal glucose (C, 5 mM glucose), high glucose (HG, 25 mM, 24h), and DNMT1 vivo morpholino (10 µM, 24h) followed by high glucose (25 mM, 24h, HG+DNMT1i). Dot plots reflecting mean±SEM of 3 independent experiments; one-way ANOVA with Sidak's multiple comparison test, **P*<0.05; ns: non-significant. **e)** P<0.0001 (C vs HG), P=0.89 (HG vs HG+DNMT1i). **f)** P=0.03 (C vs HG), P=0.17 (HG vs HG+DNMT1i).

Supp. Fig. 17 (related to Fig. 7)

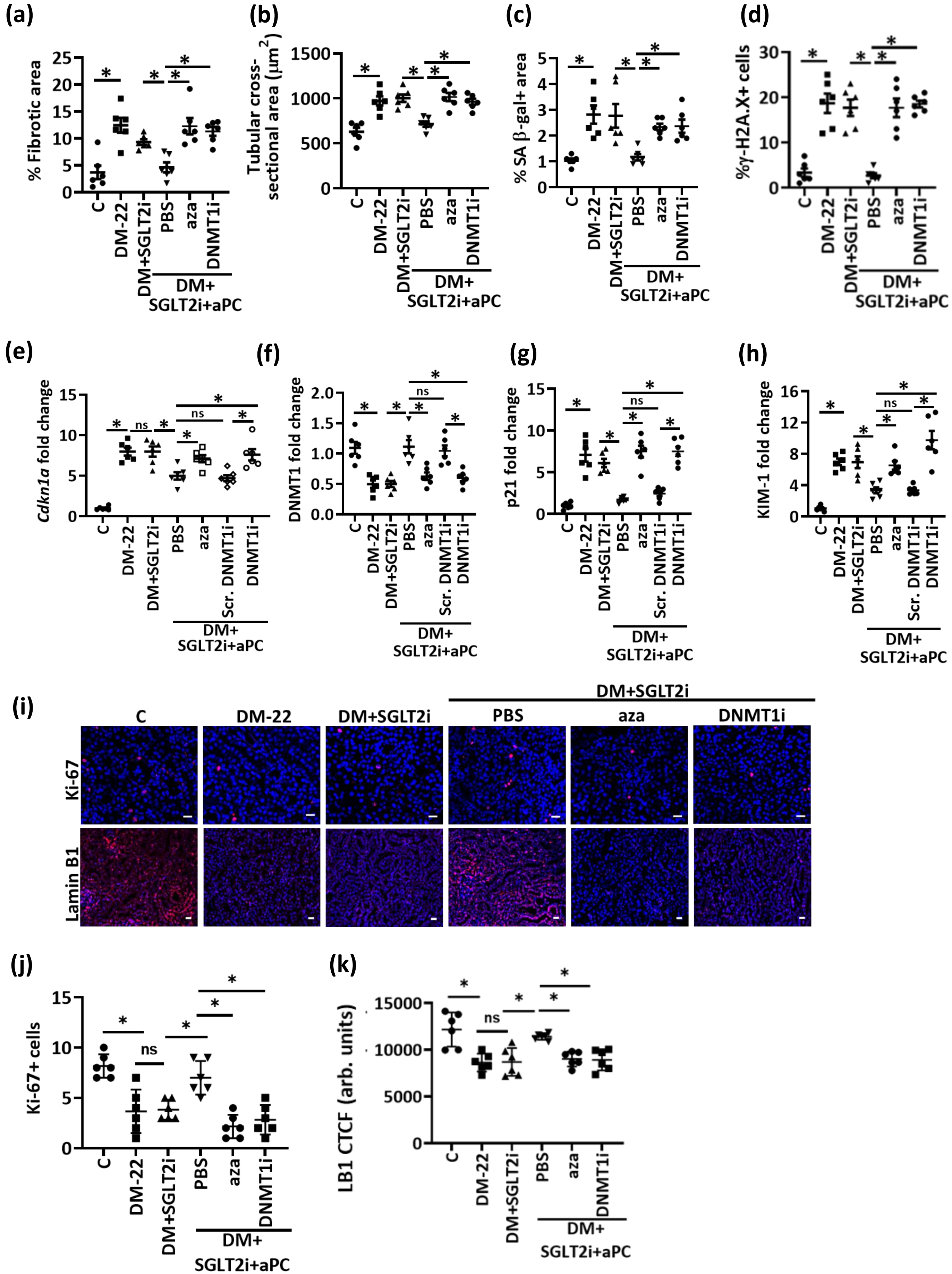

Supp. Fig. 17: aPC reverses epigenetically sustained renal p21 expression and tubular senescence in experimental DKD

a-h) Dot plots summarizing tubulointerstitital damage (fibrotic area, a; tubular cross-sectional area, b), tubular cell senescence (SA-β-gal positive area, **c**; γ-H2A.X positive cells, **d**) and renal p21 (*Cdkn1a*) mRNA **(e)** as well as KIM-1 **(f)**, p21 **(g)** and DNMT1 **(h)** protein expression in non-diabetic (C) or diabetic (DM-22) mice. Subgroups of diabetic mice were treated with SGLT2i (DM+SGLT2i), SGLT2i with aPC and PBS (DM+SGLT2i+aPC+PBS), SGLT2i with aPC and 5-aza-2'-deoxycytidine (DM+SGLT2i+aPC+aza), SGLT2i with aPC and DNMT1 vivo morpholino (DM+SGLT2i+aPC+DMNTi), or SGLT2i with aPC and scrambled vivo morpholino (DM+ SGLT2i+aPC+Scr.DMNTi). Dot plots reflecting mean±SEM of 6 mice per group; one-way ANOVA with Sidak's multiple comparison test, **P*<0.05; ns: non-significant. Specific p-values are as follows:

a) P<0.0001 (C vs DM-22), P=0.16 (SGLT2i vs aPC), P=0.0001 (aPC vs aza), P=0.0008 (aPC vs DNMT1i).
b) P<0.0001 (C vs DM-22), P=0.0001 (SGLT2i vs aPC), P<0.0001 (aPC vs aza), P=0.0009 (aPC vs DNMT1i).
c) P=0.0002 (C vs DM-22), P=0.0009 (SGLT2i vs aPC), P=0.01 (aPC vs aza), P=0.01 (aPC vs DNMT1i).
d) P<0.0001 (C vs DM-22), P<0.0001 (SGLT2i vs aPC), P<0.0001 (aPC vs aza), P<0.0001 (aPC vs DNMT1i).
e) P<0.0001 (C vs DM-22), P>0.99 (DM-22 vs SGLT2i), P=0.0008 (SGLT2i vs aPC), P=0.02 (aPC vs aza), P=0.004 (aPC vs DNMT1i), P=0.99 (aPC vs Scr. DNMT1i), P=0.001 (Scr. DNMT1i vs DNMT1i).
f) P<0.0001 (C vs DM-22), P<0.0001 (SGLT2i vs aPC), P=0.0005 (aPC vs aza), P=0.0002 (aPC vs DNMT1i), P=0.99 (aPC vs Scr. DNMT1i), P=0.001 (Scr. DNMT1i vs DNMT1i).
g) P<0.0001 (C vs DM-22), P<0.0001 (SGLT2i vs aPC), P<0.0001 (aPC vs aza), P<0.0001 (aPC vs DNMT1i), P=0.99 (aPC vs Scr. DNMT1i), P<0.0001 (Scr. DNMT1i vs DNMT1i).
h) P<0.0001 (C vs DM-22), P=0.001 (SGLT2i vs aPC), P=0.005 (aPC vs aza), P<0.0001 (aPC vs DNMT1i), P>0.99 (aPC vs Scr. DNMT1i), P<0.0001 (Scr. DNMT1i vs DNMT1i).

i-k) Exemplary histological images **(i)** and dot blots summarizing results **(j,k)** of Ki-67 and Lamin B1 staining in experimental groups (as described in **a**). Ki-67 and Lamin B1 (LB1) are immunofluorescently detected, red; DAPI nuclear counterstain, blue. Scale bars represent 20 µm. Dot plots reflecting mean±SEM of 6 mice per group; one-way ANOVA with Sidak's multiple comparison test, *P<0.05; ns: non-significant. CTCF: corrected total cell fluorescence. Specific p-values are as follows:

j) P<0.0001 (C vs DM-22), P>0.99 (DM-22 vs SGLT2i), P=0.004 (SGLT2i vs aPC), P<0.0001 (aPC vs aza), P=0.0002 (aPC vs DNMT1i).

k) P<0.0001 (C vs DM-22), P>0.99 (DM-22 vs SGLT2i), P=0.002 (SGLT2i vs aPC), P=0.008 (aPC vs aza), P=0.005 (aPC vs DNMT1i).

Supp. Fig. 18 (related to Fig. 8)

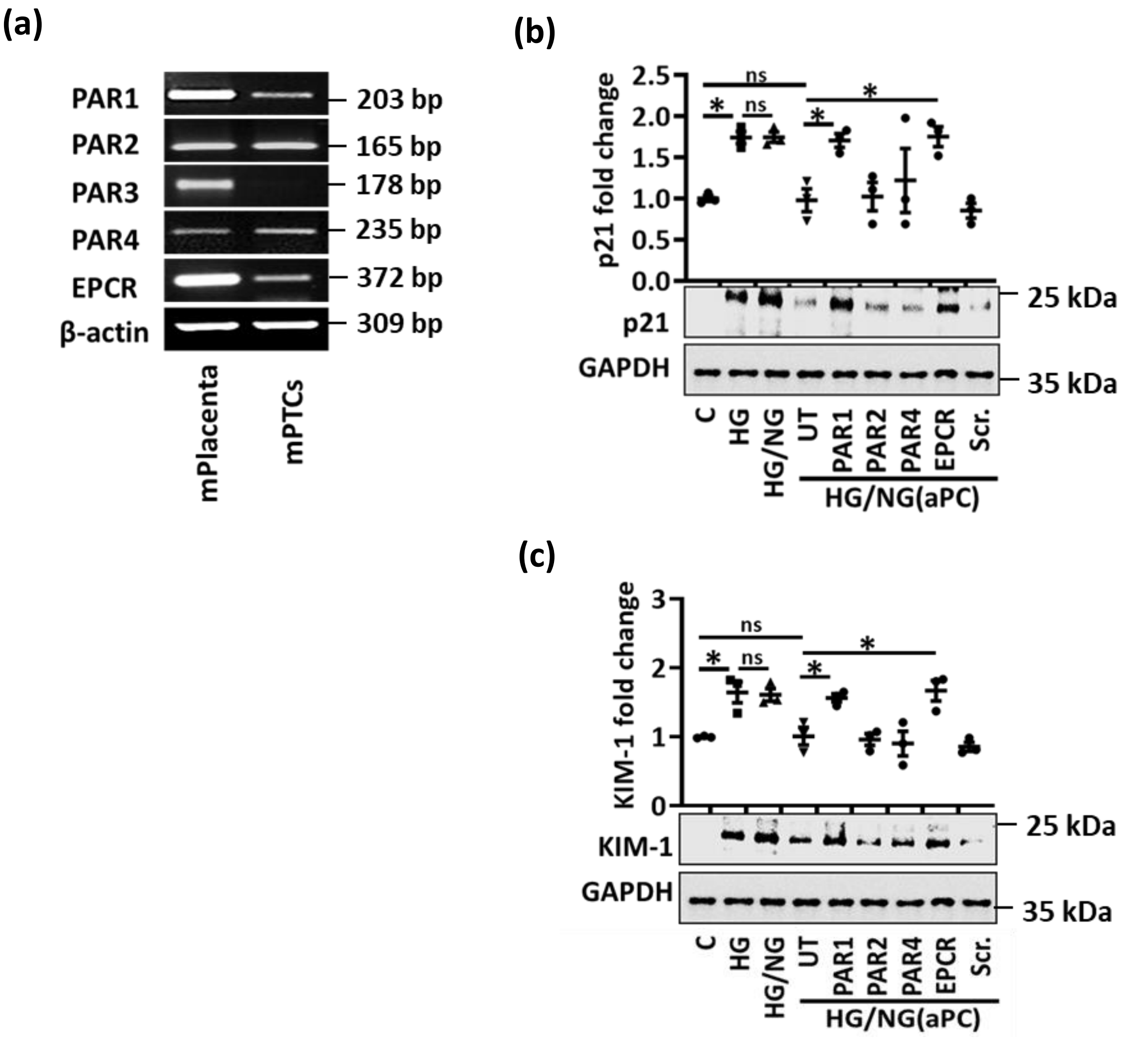

Supp. Fig. 18: aPC suppresses glucose induced tubular cell p21 expression via PAR1 and EPCR

a) Expression of PARs and EPCR in mouse proximal tubular cells (PTC; a; representative images of semiquantitative RT-PCR (n=3; mouse placenta as positive control).

b,c) aPC reverses sustained p21 **(b)** and KIM1 **(c)** expression in PTCs (HG/NG(aPC)). This effect is abolished upon PAR1 or EPCR knock down. Experimental conditions: control with continuously normal glucose (C, 5 mM glucose), continuously high glucose (HG, 25 mM, 48 h), or high glucose for 24 h followed by normal glucose (NG, 5 mM glucose) for 24 h without (HG/NG) or with aPC (20 nM) upon returning cells to normal glucose (HG/NG(aPC)). PAR1: PAR1 knock down; PAR2: PAR2 knock down; PAR4: PAR4 knock down; EPCR: EPCR knock down; Scr: non-specific, scrambled knock down construct. Exemplary immunoblots (bottom) and dot plots reflecting results (top). Dot plots reflecting mean±SEM of 3 independent experiments **(b,c)**; one-way ANOVA with Sidak's multiple comparison test, *$P<0.05$; ns: non-significant. **b)** P=0.03 (C vs HG), P>0.99 (HG vs HG/NG), P>0.99 (C vs UT), P=0.04 (UT vs PAR1), P>0.99 (UT vs PAR2), P=0.95 (UT vs PAR4), P=0.02 (UT vs EPCR), P=0.99 (UT vs Scr.). **c)** P=0.006 (C vs HG), P>0.99 (HG vs HG/NG), P>0.99 (C vs UT), P=0.02 (UT vs PAR1), P>0.99 (UT vs PAR2), P=0.99 (UT vs PAR4), P=0.005 (UT vs EPCR), P=0.97 (UT vs Scr.).

Supp. Fig. 19 (related to Fig. 8)

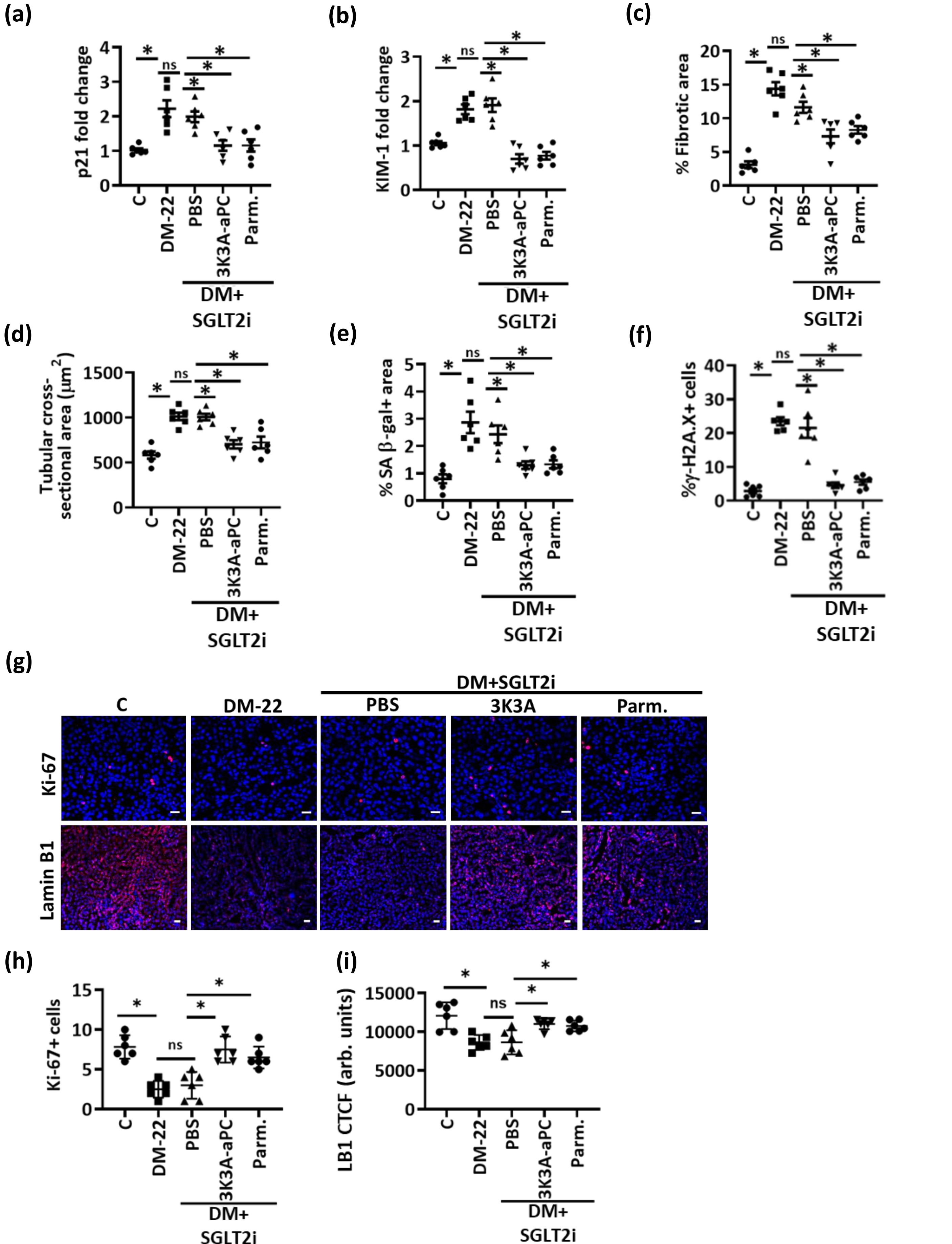

Supp. Fig. 19: aPC reverses epigenetically sustained renal p21 expression and tubular senescence independent of its anticoagulant function

a,b) Dot plots summarizing renal p21 **(a)** and KIM-1 **(b)** protein expression in non-diabetic (C) or diabetic (DM-22) mice. Subgroups of diabetic mice were treated with SGLT2i (DM+SGLT2i+PBS), SGLT2i with 3K3A-aPC (DM+SGLT2i+aPC+3K3A-aPC) or SGLT2i with parmodulin-2 (DM+SGLT2i+Parm.). Dot plots reflecting mean±SEM of 6 mice per group. one-way ANOVA with Sidak's multiple comparison test; **P*<0.05; ns: non-significant. **a)** P=0.0001 (C vs DM-22), P=0.8 (DM-22 vs PBS), P=0.006 (PBS vs 3K3A-aPC), P=0.006 (PBS vs Parm.). **b)** P=0.0001 (C vs DM-22), P=0.95 (DM-22 vs PBS), P<0.0001 (PBS vs 3K3A-aPC), P<0.0001 (PBS vs Parm.).

c-f) Dot plots summarizing tubulointerstitital damage (fibrotic area, **c**; tubular cross-sectional area, **d**) and tubular cell senescence (SA-β-gal positive area, **e**; γ-H2A.X positive cells, **f**) in experimental groups (as described in a). Dot plots reflecting mean±SEM of 6 mice per group. one-way ANOVA with Sidak's multiple comparison test; **P*<0.05; ns: non- significant. **c)** P<0.0001 (C vs DM-22), P=0.08 (DM-22 vs PBS), P=0.003 (PBS vs 3K3A-aPC), P=0.002 (PBS vs Parm.). **d)** P<0.0001 (C vs DM-22), P>0.99 (DM-22 vs PBS), P=0.0004 (PBS vs 3K3A-aPC), P=0.0009 (PBS vs Parm.). **e)** P<0.0001 (C vs DM-22), P=0.66 (DM-22 vs PBS), P=0.01 (PBS vs 3K3A-aPC), P=0.02 (PBS vs Parm.). **f)** P<0.0001 (C vs DM-22), P=0.83 (DM-22 vs PBS), P<0.0001 (PBS vs 3K3A-aPC), P<0.0001 (PBS vs Parm.).

g-i) Exemplary histological images **(g)** and dot plots summarizing results **(h,i)** of Ki-67 and Lamin B1 staining in experimental groups (as described in a). Ki-67 and Lamin B1 (LB1) are immunofluorescently detected, red; DAPI nuclear counterstain, blue. Scale bars represent 20 µm. Dot plots reflecting mean±SEM of at least 6 mice per group; one-way ANOVA with Sidak's multiple comparison test, **P*<0.05; ns: non-significant. CTCF: corrected total cell fluorescence. **h)** P<0.0001 (C vs DM-22), P=0.96 (DM-22 vs PBS), P<0.0001 (PBS vs 3K3A-aPC), P=0.001 (PBS vs Parm.). **i)** P=0.0002 (C vs DM-22), P>0.99 (DM-22 vs PBS), P=0.009 (PBS vs 3K3A-aPC), P=0.02 (PBS vs Parm.).

Uncropped blots/gels

S4a

p21

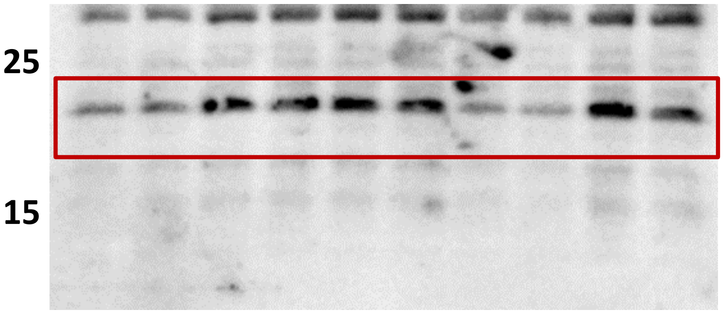

β-actin

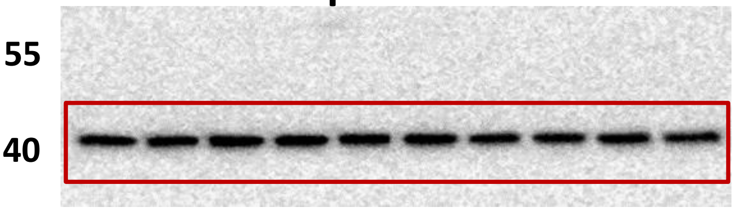

Uncropped blots/gels

S9a

p21

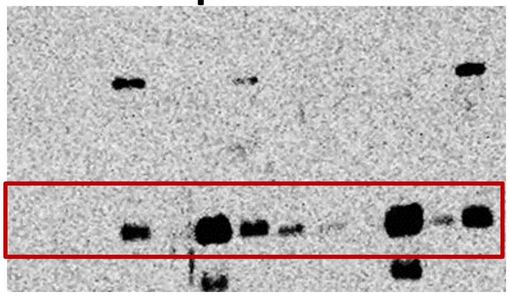

Uncropped blots/gels

S16a

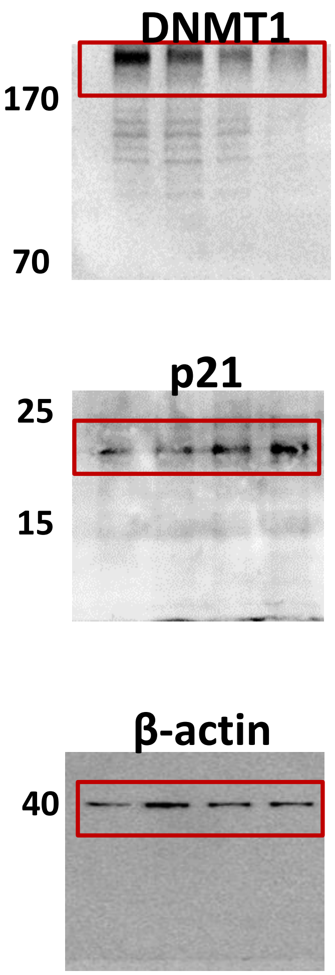

S16d

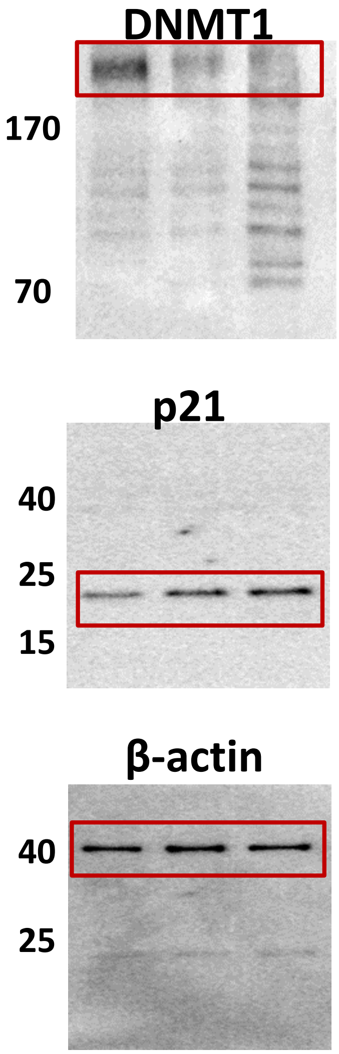

Uncropped blots/gels

S18a

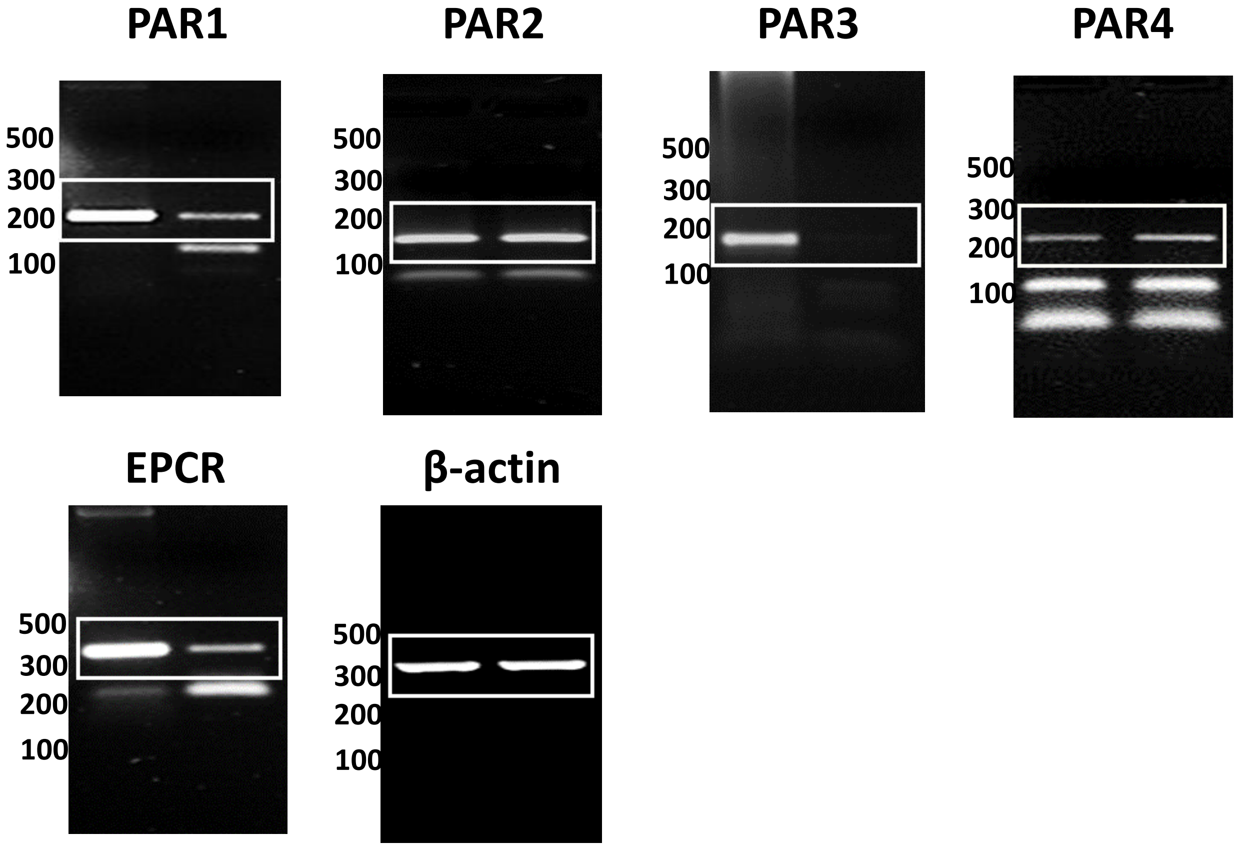

Uncropped blots/gels

S18b

p21

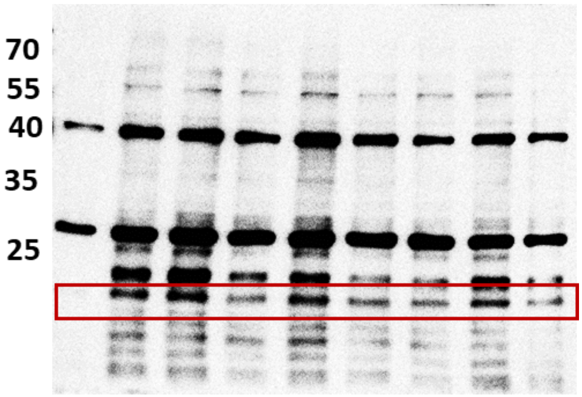

GAPDH

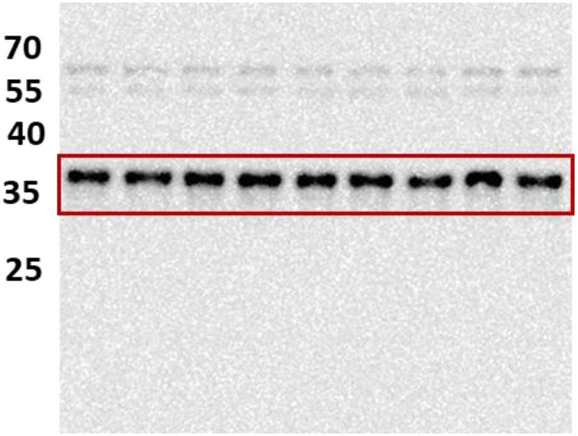

S18c

KIM-1

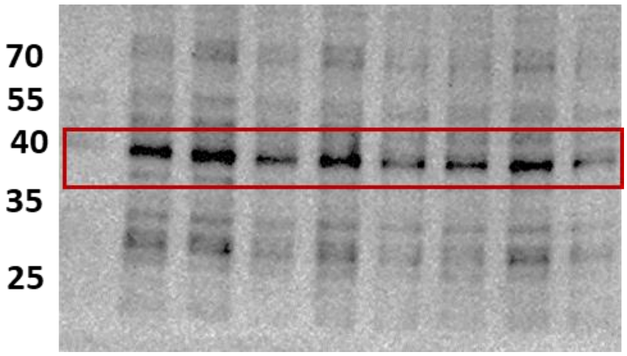

GAPDH

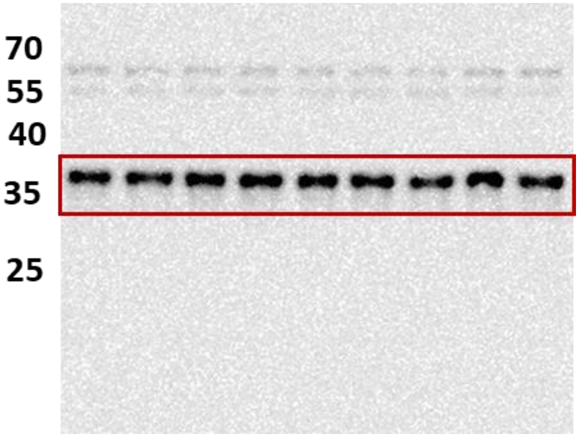

Supplementary References:

1 Isermann, B. *et al.* Activated protein C protects against diabetic nephropathy by inhibiting endothelial and podocyte apoptosis. *Nature medicine* **13**, 1349-1358, doi:10.1038/nm1667 (2007).

2 Bock, F. *et al.* Activated protein C ameliorates diabetic nephropathy by epigenetically inhibiting the redox enzyme p66Shc. *Proceedings of the National Academy of Sciences of the United States of America* **110**, 648-653, doi:10.1073/pnas.1218667110 (2013).

3 Paneni, F. *et al.* Gene silencing of the mitochondrial adaptor p66(Shc) suppresses vascular hyperglycemic memory in diabetes. *Circulation research* **111**, 278-289, doi:10.1161/CIRCRESAHA.112.266593 (2012).

4 Marquardt, A. *et al.* Farnesoid X Receptor Agonism Protects against Diabetic Tubulopathy: Potential Add-On Therapy for Diabetic Nephropathy. *Journal of the American Society of Nephrology : JASN* **28**, 3182–3189, doi:10.1681/asn.2016101123 (2017).

5 Dong, W. *et al.* Activated Protein C Ameliorates Renal Ischemia-Reperfusion Injury by Restricting Y-Box Binding Protein-1 Ubiquitination. *Journal of the American Society of Nephrology : JASN* **26**, 2789-2799, doi:10.1681/ASN.2014080846 (2015).

6 Shahzad, K. *et al.* Activated protein C reverses epigenetically sustained p66(Shc) expression in plaque-associated macrophages in diabetes. *Communications biology* **1**, 104, doi:10.1038/s42003-018-0108-5 (2018).

7 Pillebout, E. *et al.* JunD protects against chronic kidney disease by regulating paracrine mitogens. *The Journal of clinical investigation* **112**, 843-852, doi:10.1172/JCI17647 (2003).

8 Madhusudhan, T. *et al.* Signal integration at the PI3K-p85-XBP1 hub endows coagulation protease activated protein C with insulin-like function. *Blood* **130**, 1445–1455, doi:10.1182/blood-2017-02-767921 (2017).

9 McCloy, R. A. *et al.* Partial inhibition of Cdk1 in G 2 phase overrides the SAC and decouples mitotic events. *Cell cycle* **13**, 1400-1412, doi:10.4161/cc.28401 (2014).

10 Bott, S. R., Arya, M., Kirby, R. S. & Williamson, M. p21WAF1/CIP1 gene is inactivated in metastatic prostatic cancer cell lines by promoter methylation. *Prostate cancer and prostatic diseases* **8**, 321-326, doi:10.1038/sj.pcan.4500822 (2005).
