# Supplementary material for: Reversal of the renal hyperglycemic memory in diabetic kidney disease by targeting sustained tubular p21 expression
Source: Nat Commun. 2022 Aug 27;13:5062. doi: 10.1038/s41467-022-32477-9 (PMC9420151; doi:10.1038/s41467-022-32477-9)
Supplement: Supplementary file 2 — Reporting Summary [file 41467_2022_32477_MOESM2_ESM.pdf]

nature portfolio

Corresponding author(s): Berend Isermann

Last updated by author(s): Jul 7, 2022

Reporting Summary

Nature Portfolio wishes to improve the reproducibility of the work that we publish. This form provides structure for consistency and transparency in reporting. For further information on Nature Portfolio policies, see our Editorial Policies and the Editorial Policy Checklist.

Statistics

For all statistical analyses, confirm that the following items are present in the figure legend, table legend, main text, or Methods section.

n/a	Confirmed	
☐	☒	The exact sample size (*n*) for each experimental group/condition, given as a discrete number and unit of measurement
☐	☒	A statement on whether measurements were taken from distinct samples or whether the same sample was measured repeatedly
☐	☒	The statistical test(s) used AND whether they are one- or two-sided<br>*Only common tests should be described solely by name; describe more complex techniques in the Methods section.*
☒	☐	A description of all covariates tested
☐	☒	A description of any assumptions or corrections, such as tests of normality and adjustment for multiple comparisons
☐	☒	A full description of the statistical parameters including central tendency (e.g. means) or other basic estimates (e.g. regression coefficient) AND variation (e.g. standard deviation) or associated estimates of uncertainty (e.g. confidence intervals)
☐	☒	For null hypothesis testing, the test statistic (e.g. F, t, r) with confidence intervals, effect sizes, degrees of freedom and P value noted<br>*Give P values as exact values whenever suitable.*
☒	☐	For Bayesian analysis, information on the choice of priors and Markov chain Monte Carlo settings
☒	☐	For hierarchical and complex designs, identification of the appropriate level for tests and full reporting of outcomes
☐	☒	Estimates of effect sizes (e.g. Cohen's d, Pearson's r), indicating how they were calculated

Our web collection on statistics for biologists contains articles on many of the points above.

Software and code

Policy information about availability of computer code

Data collection	Fluorescence and absorbance measurement: Gen5 software<br>Western Blotting and PCR-gels: cseries capture software, Azure c400.<br>Microscopy: Olympus cellSens Dimension 1.5 Image software, Leica Application Suite X (LAS X) software 8, Leica.<br>qRT-PCR: CFX Maestro Software 2.3
Data analysis	Statistical analysis was performed using GraphPad Prism 9 software.<br>Images were analyzed using NIH Image J v1.48<br>Functional annotation was performed using Pantherdb version 16.0 (http://www.pantherdb.org/)<br>Sequencing data was analyzed using Trim Galore! wrapper, R packages: Rsubread, bioMaRt, edgeR, Limma.

For manuscripts utilizing custom algorithms or software that are central to the research but not yet described in published literature, software must be made available to editors and reviewers. We strongly encourage code deposition in a community repository (e.g. GitHub). See the Nature Portfolio guidelines for submitting code & software for further information.

Data

Policy information about availability of data

All manuscripts must include a data availability statement. This statement should provide the following information, where applicable:

- Accession codes, unique identifiers, or web links for publicly available datasets
- A description of any restrictions on data availability
- For clinical datasets or third party data, please ensure that the statement adheres to our policy

The data availability statement is given in the manuscript.

Field-specific reporting

Please select the one below that is the best fit for your research. If you are not sure, read the appropriate sections before making your selection.

☒ Life sciences ☐ Behavioural & social sciences ☐ Ecological, evolutionary & environmental sciences

For a reference copy of the document with all sections, see nature.com/documents/nr-reporting-summary-flat.pdf

Life sciences study design

All studies must disclose on these points even when the disclosure is negative.

Sample size	In a sub group of experiments sample sizes were chosen based on previous experiences (PMIDs: 32709711, 28687614, 25075770, 17982464): WT-diabetic, control mice<br>TMPro-diabetic, control mice.<br>In all others, an estimate was made based on effect size and power of the study using software G-Power (v3.1.9.7).
Data exclusions	Data points were not excluded.
Replication	The findings were replicated using multiple cohorts (LIFE-cohort, Outpatient clinics, FMD study, HEIST-DiC study), repeat experiments and using technical replicates. Three technical replicates were for example used to confirm ELISA measurements of urinary p21. In case of in vitro experiments, three independent repeat experiments were performed. In case of in vivo experiments, a sample size was calculated (as descrived above) to ensure biological replicates.
Randomization	For in vivo experiments, mice were purchased or obtained from the routine breeding colony and randomly distrubuted to groups. Care was taken that each group contained age and sex-matched mice. For human samples, samples were matched for key clinical features as shown in supplemetnary tables. In some analyses, patients samples were obtained from clinical routine in a sequential order and control samples were matched.<br>For in vitro experiments, cells were routinely passaged and randomly allocated for different treatment groups.
Blinding	The experimentator was blinded while performing the ex-vivo analysis of the mice. However, blinding was not performed while performing in vivo interventions. This was done to ensure that the correct intervention was applied to each group as allocated. This was also important to ensure animal ethical guidelines and scoring based on the interventions.<br><br>For experiments other than those involving mice work, the experimentator was blinded to group allocation during data collection and analysis.

Reporting for specific materials, systems and methods

We require information from authors about some types of materials, experimental systems and methods used in many studies. Here, indicate whether each material, system or method listed is relevant to your study. If you are not sure if a list item applies to your research, read the appropriate section before selecting a response.

Materials & experimental systems

n/a	Involved in the study
☐	☒ Antibodies
☐	☒ Eukaryotic cell lines
☒	☐ Palaeontology and archaeology
☐	☒ Animals and other organisms
☐	☒ Human research participants
☐	☒ Clinical data
☒	☐ Dual use research of concern

Methods

n/a	Involved in the study
☒	☐ ChIP-seq
☒	☐ Flow cytometry
☒	☐ MRI-based neuroimaging

Antibodies

Antibodies used	The source and company of the antibodies used in the study are mentioned in the methods section. Other details are available upon request.<br>Antibody Company Cat. no. Clone Dilution<br>rabbit anti-p21 Santa Cruz Biotechnology sc-397 C-19 1:400<br>rabbit anti-megalin Santa Cruz Biotechnology sc-25470 H-245 1:100<br>mouse-anti synaptopodin Santa Cruz Biotechnology sc-515842 D-9 1:400<br>rabbit anti-DNMT1 Cell Signaling Technology 5032 D63A6 1:1000<br>rabbit anti-human p21 Cell Signaling Technology 2947 12D1 1:1000<br>rabbit anti-γ-H2A.X Cell Signaling Technology 9718 20E3 1:1000<br>rabbit anti-Ki-67 Cell Signaling Technology 9129 D3B5 1:350<br>rabbit anti-Lamin B1 Cell Signaling Technology 17416 E6M5T 1:500

	rabbit anti-β-actin Cell Signaling Technology 4970 13E5 1:2000<br>anti-rabbit IgG, HRP-linked Cell Signaling Technology 7074 NA 1:4000<br>rabbit anti-KIM-1 Abcam ab47635 NA 1:2000<br>rabbit anti-mouse p21 Abcam ab188224 EPR18021 1:800<br>rabbit anti-GAPDH Sigma-Aldrich G9545 NA 1:25000<br>goat anti-mouse nephrin R&D systems AF3159 NA 1:300<br>anti-mouse/rabbit IgG polymer, HRP-linked R&D systems VC002-025 NA direct<br>rabbit anti-WT-1 Abnova MAB20854 CEH-23 1:350<br>anti-rabbit IgG, texas red linked Vector Laboratories TI-1000 NA 1:400<br>anti-goat IgG, fluorescein linked Vector Laboratories FI-5000 NA 1:400
Validation	The antibodies used within the study were validated by the company. Wherever possible, antibodies were internally validated using knockout tissues.

Eukaryotic cell lines

Policy information about cell lines

Cell line source(s)	Cell lines used within the study were obtained from ATCC or from collaborators.<br>HEK-293: ATCC, Cat No: CRL-1573<br>BUMPT cells: Dr. Wilfred Lieberthal (Boston University), USA.<br>MES13: ATCC, , Cat No: CRL-1927<br>Mouse glomerular endothelial cells: Cell Biologics, Cat No.: C57-6014G<br>Immortalized mouse podocytes: Prof. Jochen Reiser, USA.
Authentication	Cell lines were validated for species using species specific primers. Cell type specific markers were tested using RT-PCR.
Mycoplasma contamination	Cell lines were routinely tested negative for mycoplasma.
Commonly misidentified lines<br>(See ICLAC register)	No commonly misidentified cell lines were used.

Animals and other organisms

Policy information about studies involving animals; ARRIVE guidelines recommended for reporting animal research

Laboratory animals	Strain: C57BL/6, C57BLKsJ-db/+ (db/m) and C57BL/KSJ-db (db/db)<br>Age: starting 8 weeks of age<br>Mice of both sexes were included in the study and the groups were matched for age and sex.<br>Housing conditions: Temperature 21 °C ± 2, RH 55 % ± 15, 10 air changes, 12 h/12 h day/night, Barrier housing in open cages for breeding and husbandry purposes, as well as experimental areas.
Wild animals	No wild animals were used.
Field-collected samples	No field collected samples were used.
Ethics oversight	All animal experiments were conducted following standards and procedures approved by the local Animal Care and Use Committee (Landesverwaltungsamt Halle and Leipzig, Germany).

Note that full information on the approval of the study protocol must also be provided in the manuscript.

Human research participants

Policy information about studies involving human research participants

Population characteristics	Population characteristics are mentioned within supplementary tables S1-S5 together with clinical parameters tested.
Recruitment	Urine samples were randomly taken from the rest-material of the diagnostics routine lab at university hospital Leipzig. Urine samples were also obtained from the LIFE-biobank which contained samples from the LIFE-Adult study. For the FMD study, patients were selected from the diabetic outpatient clinic at the university hospital of Heidelberg and included in the study based on the inclusion criteria.
Ethics oversight	Human urine samples obtained from the local outpatient clinic were obtained based on informed consent (Ethic vote no: 082-10-19042010, University of Leipzig).<br>LIFE-ADULT cohort (Ethic vote no: 263-2009-14122009 and 201/17-ek, University of Leipzig).<br>Urine samples from the FMD study (DRKS00014287) and HEIST-DiC study were obtained after ethical approval (Ethic vote no: 383/2016, 204/2004 and 682/2016, Ruprecht-Karls-University of Heidelberg) and informed consent.

Note that full information on the approval of the study protocol must also be provided in the manuscript.

Clinical data

Policy information about clinical studies

All manuscripts should comply with the ICMJE guidelines for publication of clinical research and a completed CONSORT checklist must be included with all submissions.

Clinical trial registration	DRKS00014287 (German Clinical Trials Register)
Study protocol	https://www.drks.de/drks_web/
Data collection	Albuminuria was measured at baseline, after 3 months and after 6 months using a quantitative method for the albumin concentration in urine. These are described in manuscript with PMID: 5661214.<br>For the current manuscript, only p21 was measured and correlated with already available data. p21 was measured using a commerical ELISA under standard laboratory conditions.
Outcomes	Primary endpoint of the trial was diabetic nephropathy. This was evaluted by the change of albuminuria. This was not a part of the current manuscript and are described in a separate publication. (PMID: 35661214).<br>Composite endpoint score of diabetic late complications were secondary outcomes. Prespecified exploratory endpoints comprised change in homeostatic model assessment for insulin resistance (HOMA-IR) as marker for insulin resistance, change in plasma methylglyoxal-derived hydroimidazolone 1 (MG-H1), plasma methylglyoxal (MG), glyoxalase-1 (Glo-1) activity, and expression of phosphorylated Glo-1 (pGlo-1) in white blood cells (WBC), hydroxyacetone in red blood cells as markers of dicarbonyl detoxification, change in plasma acylcarnitine (AC) profile as a marker of fatty acid oxidation, change in phosphorylated histone H2AX (γH2Ax) expression in WBC as marker of DNA damage/repair, and change in soluble urokinase plasminogen activator receptor (suPAR), as a marker of kidney injury and senescence. These outcomes were not a part of this study and are described in a separate publication (PMID: 35661214).<br>For the current study only p21 was measured and correlated with previously available data.
