# Supplementary material for: Reversal of the renal hyperglycemic memory in diabetic kidney disease by targeting sustained tubular p21 expression
Source: Nat Commun. 2022 Aug 27;13:5062. doi: 10.1038/s41467-022-32477-9 (PMC9420151; doi:10.1038/s41467-022-32477-9)
Supplement: Supplementary file 3 — Source data [file 41467_2022_32477_MOESM3_ESM.zip › Source data/Source data-uncropped blots and gels.pptx]

## Slide 1
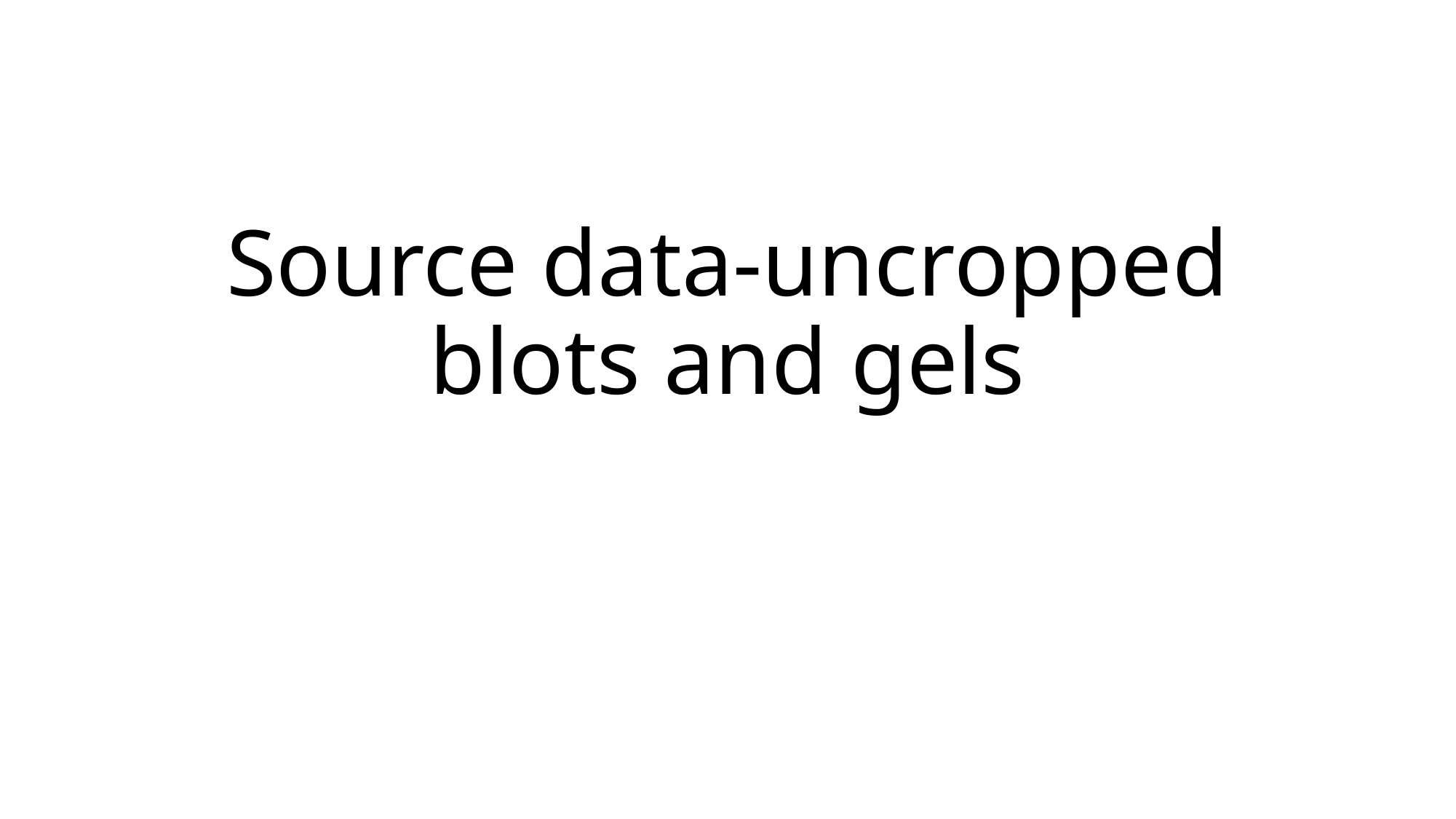

# Source data-uncropped blots and gels

## Slide 2
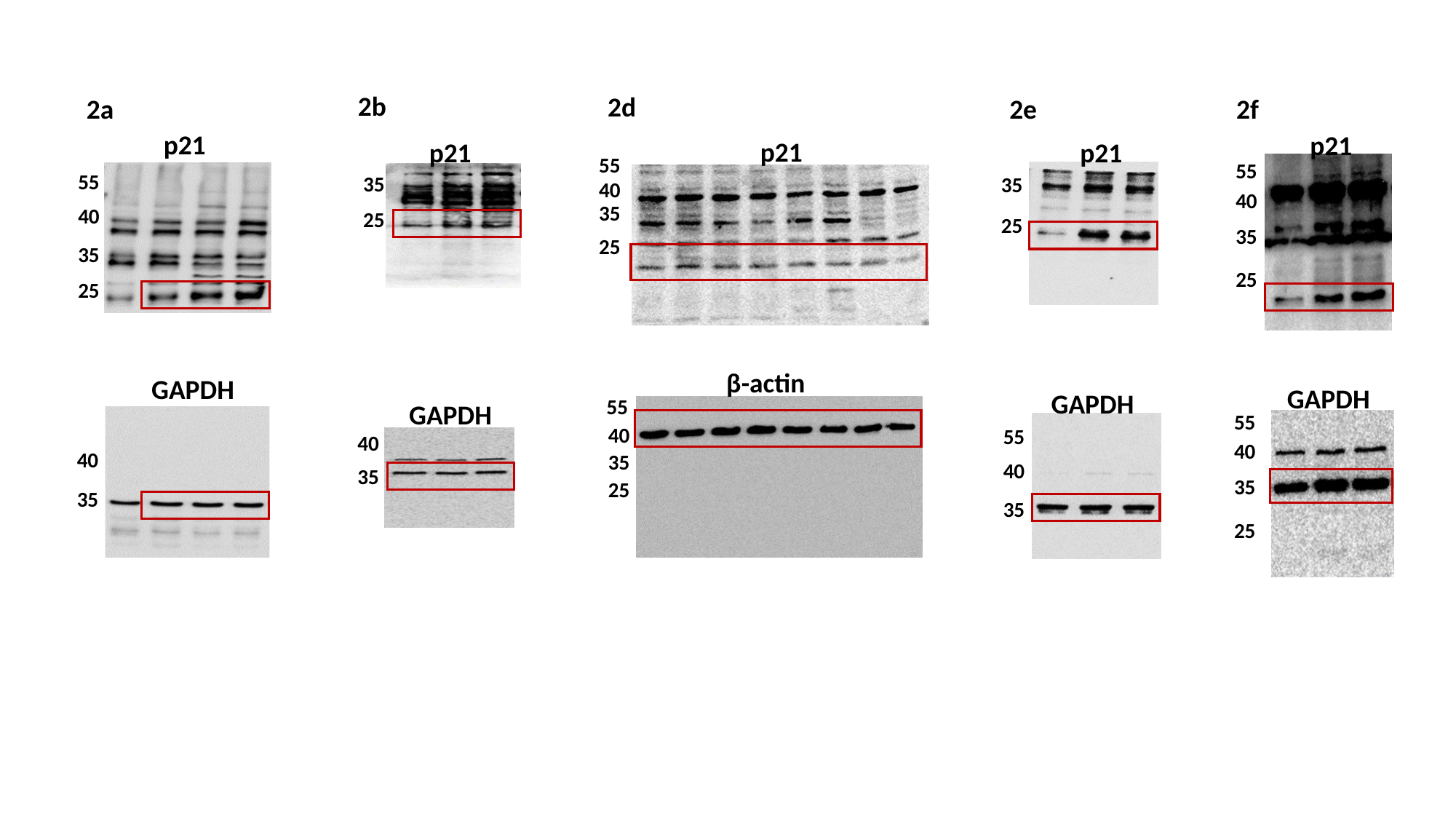

2b
2d
2a
2f
2e
p21
p21
p21
p21
p21
55
40
35
25
55
40
35
25
35
25
55
40
35
25
35
25
β-actin
GAPDH
GAPDH
GAPDH
55
40
35
25
GAPDH
55
40
35
25
40
35
55
40
35
40
35

## Slide 3
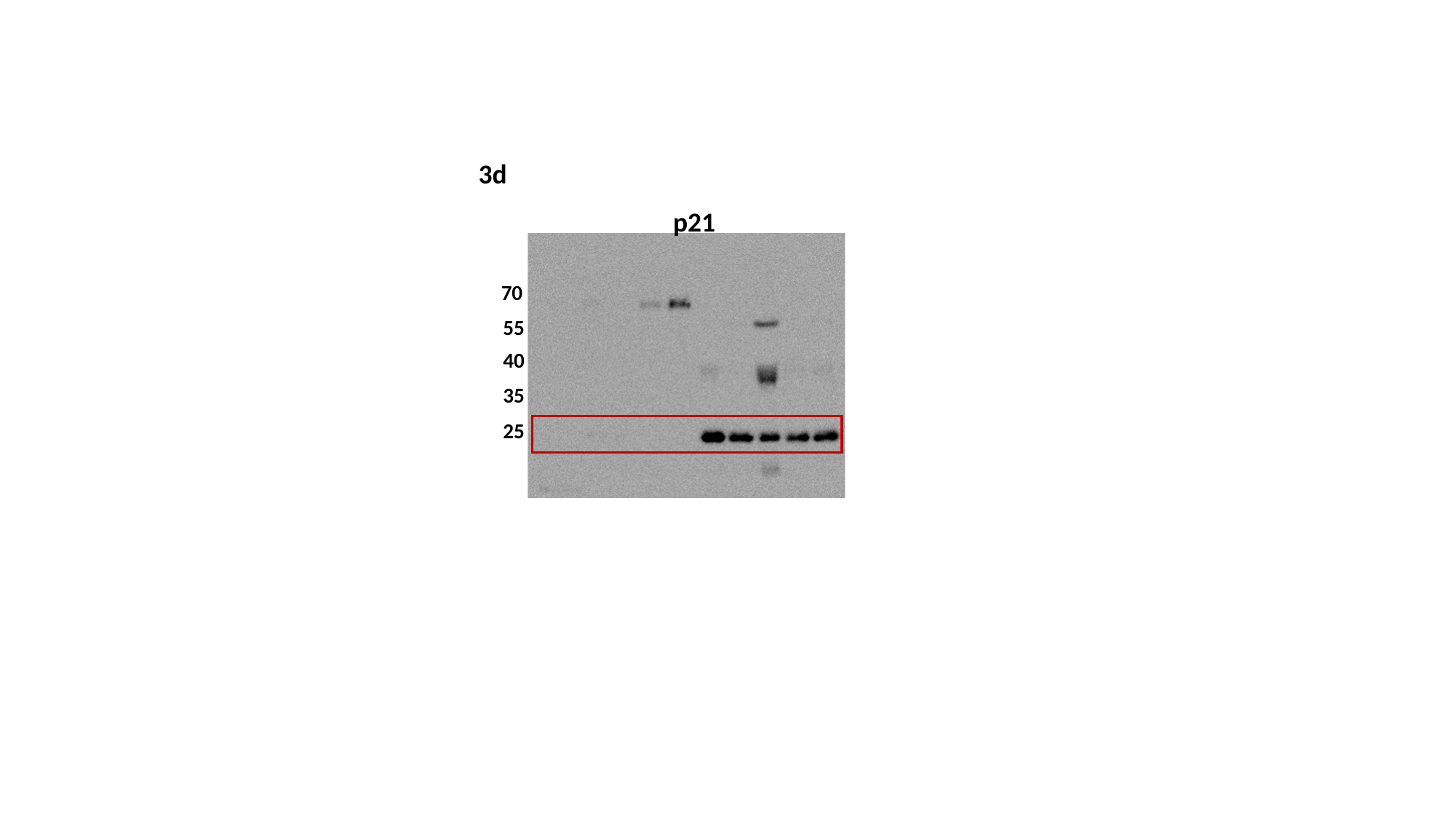

3d
p21
70
55
40
35
25

## Slide 4
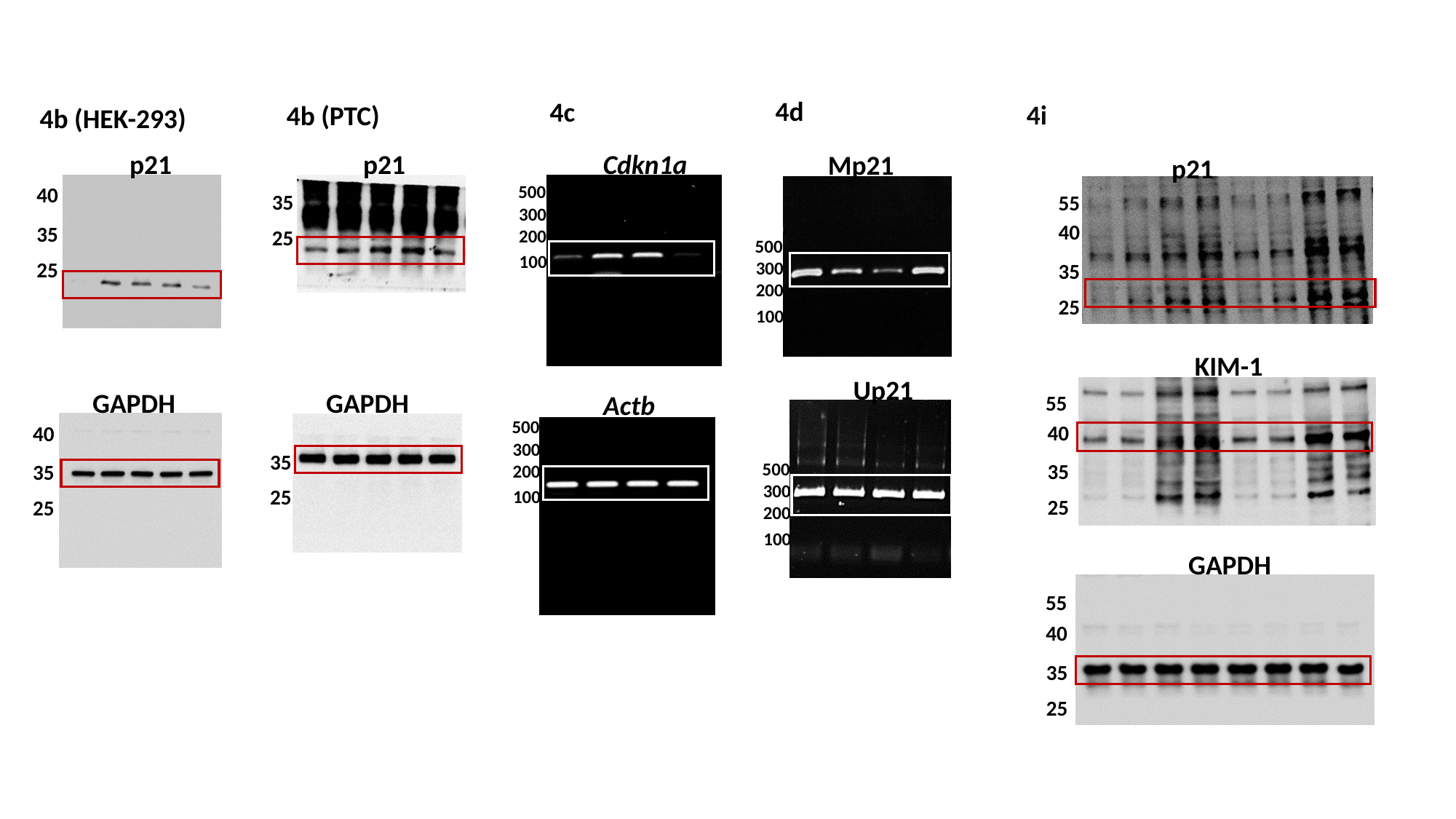

4d
4c
4i
4b (PTC)
4b (HEK-293)
p21
p21
Cdkn1a
Mp21
p21
500
40
35
25
35
25
55
40
35
25
300
200
500
100
300
200
100
KIM-1
Up21
55
40
35
25
GAPDH
GAPDH
Actb
500
40
35
25
35
25
300
500
200
300
100
200
100
GAPDH
55
40
35
25

## Slide 5
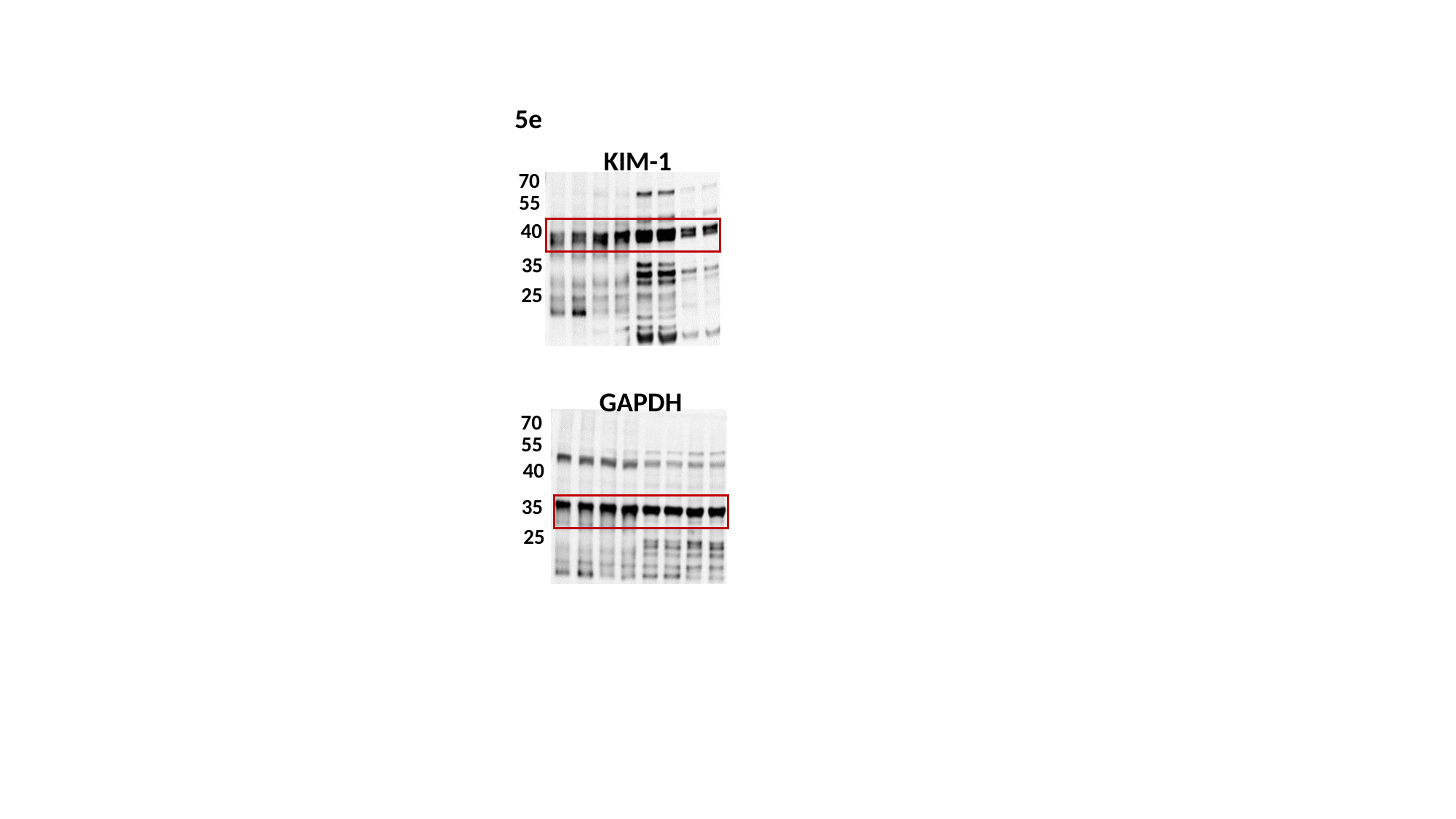

5e
KIM-1
70
55
40
35
25
GAPDH
70
55
40
35
25

## Slide 6
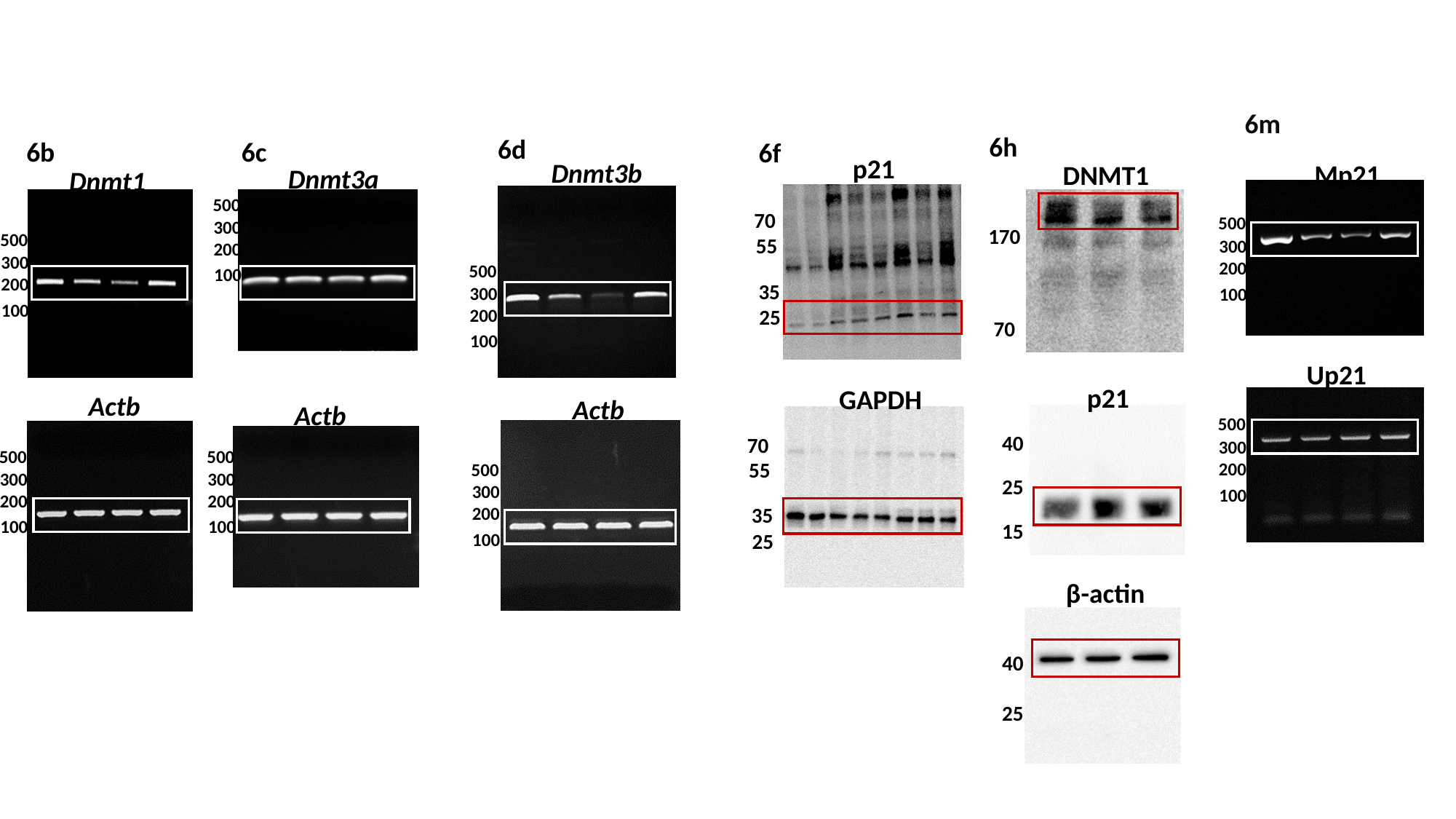

6m
6h
6d
6b
6c
6f
p21
Dnmt3b
Mp21
DNMT1
Dnmt3a
Dnmt1
70
55
35
25
500
170
70
500
300
500
300
200
300
200
500
100
200
300
100
100
200
100
Up21
p21
GAPDH
Actb
Actb
Actb
40
25
15
70
55
35
25
500
300
500
500
200
500
300
300
300
100
200
200
200
100
100
100
β-actin
40
25

## Slide 7
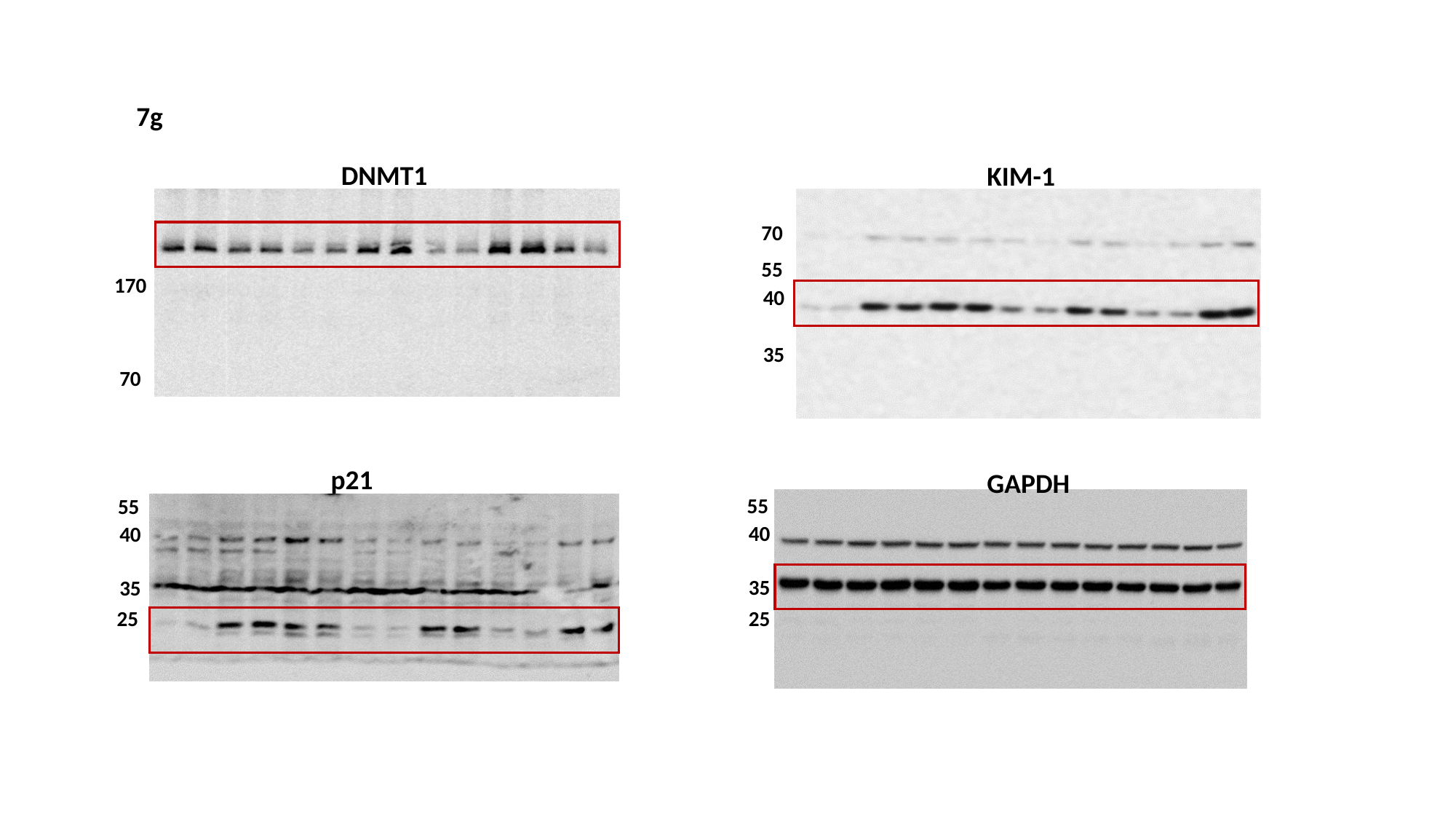

7g
DNMT1
KIM-1
170
70
70
55
40
35
p21
GAPDH
55
40
35
25
55
40
35
25

## Slide 8
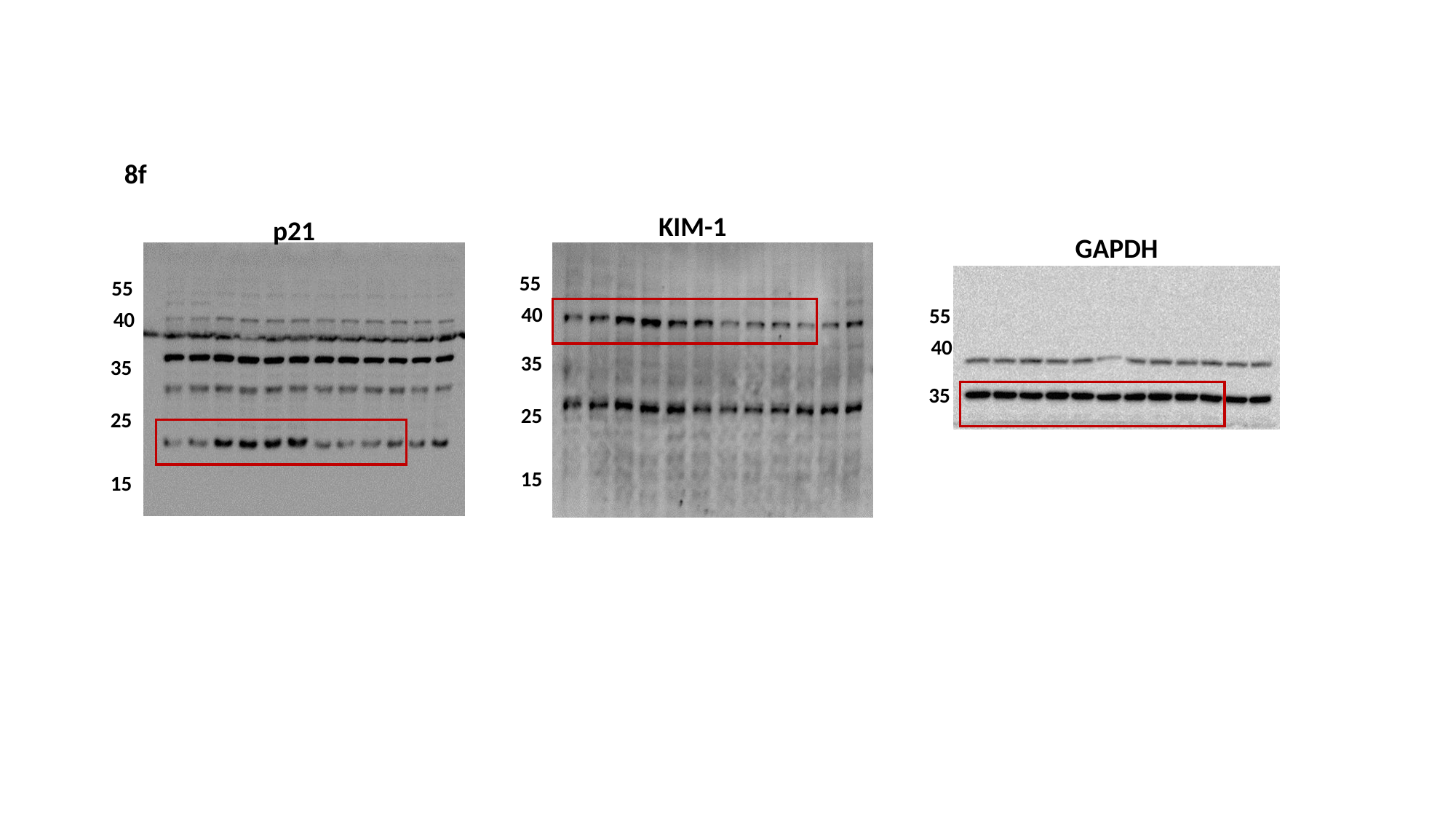

8f
KIM-1
p21
GAPDH
55
40
35
25
15
55
55
40
35
40
35
25
15
